# The Binding of *Plasmodium falciparum* Adhesins and Erythrocyte Invasion Proteins to Aldolase Is Enhanced by Phosphorylation

**DOI:** 10.1371/journal.pone.0161850

**Published:** 2016-09-08

**Authors:** Suraya A. Diaz, Stephen R. Martin, Steven A. Howell, Munira Grainger, Robert W. Moon, Judith L. Green, Anthony A. Holder

**Affiliations:** 1 Malaria Parasitology Laboratory, The Francis Crick Institute, Mill Hill Laboratory, The Ridgeway, Mill Hill, London, NW7 1AA, United Kingdom; 2 Structural Biology Science Technology Platform The Francis Crick Institute, Mill Hill Laboratory, The Ridgeway, Mill Hill, London, NW7 1AA, United Kingdom; 3 Mass Spectrometry Science Technology Platform, The Francis Crick Institute, Mill Hill Laboratory, The Ridgeway, Mill Hill, London, NW7 1AA, United Kingdom; Ehime Daigaku, JAPAN

## Abstract

Aldolase has been implicated as a protein coupling the actomyosin motor and cell surface adhesins involved in motility and host cell invasion in the human malaria parasite *Plasmodium falciparum*. It binds to the cytoplasmic domain (CTD) of type 1 membrane proteins of the thrombospondin-related anonymous protein (TRAP) family. Other type 1 membrane proteins located in the apical organelles of merozoites, the form of the parasite that invades red blood cells, including apical membrane antigen 1 (AMA1) and members of the erythrocyte binding ligand (EBL) and reticulocyte binding homologue (RH) protein families have been implicated in host cell binding and invasion. Using a direct binding method we confirm that TRAP and merozoite TRAP (MTRAP) bind aldolase and show that the interaction is mediated by more than just the C-terminal six amino acid residues identified previously. Single amino acid substitutions in the MTRAP CTD abolished binding to aldolase. The CTDs of AMA1 and members of the EBL and RH protein families also bound to aldolase. MTRAP competed with AMA1 and RH4 for binding to aldolase, indicating overlapping binding sites. MTRAP CTD was phosphorylated *in vitro* by both calcium dependent kinase 1 (CDPK1) and protein kinase A, and this modification increased the affinity of binding to aldolase by ten-fold. Phosphorylation of the CTD of members of the EBL and RH protein families also increased their affinity for aldolase in some cases. To examine whether or not MTRAP expressed in asexual blood stage parasites is phosphorylated, it was tagged with GFP, purified and analysed, however no phosphorylation was detected. We propose that CTD binding to aldolase may be dynamically modulated by phosphorylation, and there may be competition for aldolase binding between different CTDs. The use and efficiency of alternate invasion pathways may be determined by the affinity of adhesins and cell invasion proteins for aldolase, in addition to their host ligand specificity.

## Introduction

*Plasmodium falciparum* is an apicomplexan parasite that causes the most virulent form of human malaria. All of the pathology of the disease is caused by the asexual blood-stage parasite, which develops and multiplies within red blood cells (RBC). Intracellular development through the trophozoite and schizont phases results in a multinucleate cell and then cell division produces individual haploid merozoites. This process is accompanied by the elaboration of apical organelles such as micronemes and rhoptries, and an inner membrane complex (IMC) that forms part of the parasite’s surface pellicle. Following release (or egress) from the schizont, the extracellular merozoites invade new RBC and the cycle continues. The invasion of RBC is a crucial step in the parasite life-cycle and, because the parasite's exposed surface antigens are especially susceptible to immune attack, rapid host cell entry is important. Interactions between parasite and host cell proteins are very important in this process.

The full life-cycle of this parasite is complex and includes other extracellular stages that are motile and that invade host cells and tissue. The motile sporozoite and ookinete stages move by a process called gliding motility, which is powered by a multi-molecular complex called the glideosome. In the current model, the glideosome, containing a class XIV myosin, myosin A (MyoA); a myosin light chain called the MyoA tail domain-interacting protein (MTIP); and glideosome-associated proteins (GAPs) such as GAP50, GAP45, and GAP40 [1], is located beneath the parasite's plasma membrane (PM) and is attached to the IMC to provide the force for the translocation of actin filaments. The actin filaments are thought to be connected through an interaction with the tetrameric fructose 1,6-bisphosphate aldolase [2] to the C-terminal cytoplasmic domain (CTD) of proteins, which provides the extracellular linkage to the motor [3, 4]. In sporozoite stage parasites, the thrombospondin-related anonymous protein (TRAP) has been identified as the adhesin that couples the intracellular movement of actin filaments to the outside of the cell through binding of the extracellular domain of TRAP to a substrate matrix or the surface of a host cell [5], and of the CTD to aldolase [3, 6, 7]. These components (TRAP-aldolase-actin-glideosome) constitute the general motor complex to power sporozoite motility, with key features conserved in many apicomplexan parasites [1, 3].

Host cell and tissue invasion may be driven by the same motor components that power motility. The basic motor complex is present in the blood stage non-gliding but erythrocyte-invasive merozoites with MTRAP (merozoite TRAP) suggested to fulfil the same function as TRAP in sporozoites [1, 8], and binding to the Semaphorin-7A receptor on the surface of the erythrocyte [9]. MTRAP is a micronemal protein that shares key features with TRAP, including a thrombospondin repeat (TSR) domain, a putative rhomboid-protease cleavage site, and a CTD with a conserved sub-terminal tryptophan residue [1]. MTRAP may play an essential role in asexual blood stages as it was not possible to knock out the corresponding gene, and it interacts *in vitro* with aldolase [1], suggesting that it is the merozoite-specific functional homologue of TRAP. However, although MTRAP resembles TRAP structurally, its precise role in merozoite invasion of erythrocytes has not been studied in detail.

The merozoite is a highly polarized cell with the specialized apical subcellular organelles that participate in RBC invasion. Invasion comprises a series of complex molecular events including several molecular interactions and signal transduction between parasite and host cell [10]. The merozoite first establishes low affinity reversible interactions with the host cell [10], and then the parasite orientates, to place its apical end towards the erythrocyte surface [11–13] in preparation for formation of the moving junction between the merozoite and the erythrocyte [14–20]. Several adhesins and invasion proteins that are class 1 membrane proteins and initially located in the apical organelles are involved in this process, including apical membrane antigen 1 (AMA1), and the erythrocyte binding ligand (EBL) and reticulocyte binding homologue (RH) protein families. Secretion of the rhoptry neck (RON) protein complex into the erythrocyte plasma membrane and further interactions with AMA1 [21, 22] establish the moving junction, which is part of the mechanism through which the parasite actively invades, probably using the motor complex to generate the driving force required.

In addition to the TRAP family interaction with aldolase, these other adhesins and invasion proteins may interact with aldolase. It has been reported that *P*. *falciparum* AMA1 is able to bind to rabbit muscle aldolase and that this binding is inhibited competitively by the aldolase substrate, fructose-1,6-bisphosphate, suggesting that the cytoplasmic tail of AMA1 interacts with the substrate binding pocket of aldolase, in a manner similar to TRAP [23]. In addition, the CTD of members of the RH family bind to rabbit muscle aldolase, with the binding disrupted by mutations in terminal Tyr or Phe residues [24]. The CTDs of two members of the EBL family do not bind to rabbit aldolase [24]. However, an interaction between AMA1 and the EBL and RH family proteins with *P*. *falciparum* aldolase has not been examined and the role the CTD of these proteins is not fully understood.

TRAP CTD binding to aldolase involves a key conserved tryptophan residue located within the six C-terminal residues of the CTD (EENEWN). Crystallographic studies have shown that this peptide occludes the active site of aldolase and the tryptophan residue is buried within the aldolase catalytic pocket, thus inhibiting enzyme activity. Substitution of the tryptophan by alanine resulted in peptides that either bound weakly outside the active site or did not bind at all [25–28]. Studies of rabbit actin binding to aldolase had shown that there is partial overlap of the aldolase catalytic and TRAP- and actin-binding sites [25–27], with the protein-binding residues clustered in and around the catalytic pocket of the molecule. Although the binding of one ligand may preclude concurrent binding of the other to the same monomer [29–31], the tetrameric aldolase provides four binding sites and the potential for multiple protein interactions.

Phosphorylation of the CTD is also likely to be important. For example, The CTD of AMA1 in schizonts is phosphorylated in at least 5 positions including residue 610 [32], and substitution of the serine at this position by alanine had a dramatic negative effect on erythrocyte invasion [33]. Likewise Ser3233 in the CTD of RH2b was found to be phosphorylated in schizonts prior to RH2b translocation from the rhoptry neck to the parasite PM [34], and phosphorylation of RH4 is important for successful merozoite invasion [35]. Protein kinase A (PKA) and casein kinase 2 (CK2) are two of the kinases responsible. Several kinases including calcium dependent protein kinase 1 (CDPK1), protein kinase B, and PKA have been shown to phosphorylate glideosome components such as MyoA, MTIP and GAP45 [36], highlighting the potential importance of phosphorylation in regulating parasite motor complex machinery [37]. Therefore it would be of interest to determine whether other CTDs are also phosphorylated and whether or not this affects their binding properties, for example to aldolase.

In recent studies in *Toxoplasma gondii* the role of the actomyosin motor [38, 39] and aldolase [40] in cell invasion have been questioned. Whilst the binding of both *Babesia* and *P*. *falciparum* CTDs directly to actin *in vitro* [41, 42] or the ability of glyceraldehyde-3-phosphate dehydrogenase to substitute for aldolase [24] may explain why aldolase is not essential, the detail of what drives cell invasion needs to be elucidated.

In this study we have investigated the binding of the CTDs of several adhesins and cell invasion proteins to malarial parasite aldolase. We were able to demonstrate that several CTDs bind to *P*. *falciparum* aldolase and that the interaction may be strengthened by phosphorylation of certain serine or threonine residues. We propose that the interaction between these apical proteins and aldolase is regulated by phosphorylation/dephosphorylation and this may modulate erythrocyte invasion.

## Results

### Adhesins and cell invasion proteins bind to aldolase

CTDs of four classes of type 1 membrane protein (AMA1 and the TRAP, EBL and RH families) that are potential adhesins or host cell invasion proteins derived from apical organelles, and present in the merozoite plasma membrane during invasion [14, 16, 21, 22, 43], were examined for their ability to bind to aldolase using bio-layer interferometry. Streptavidin-coated biosensors were loaded with biotinylated synthetic peptides or recombinant proteins representing either the full length CTDs or shorter C-terminal sequences (Table 1) and titrated with increasing aldolase concentrations. This analysis enabled the calculation of a dissociation constant (K_d_) for each pair of proteins as a measure of the strength of interaction between them. As a negative control peptide we used the sequence corresponding to the CTD of the protein PTRAMP, which does not bind to aldolase. The data show that the cytoplasmic tails of AMA1 and the TRAP, EBL and RH family proteins are able to interact with aldolase, however the strength of the interaction differs substantially for the different CTDs.

**Table 1:** Dissociation constants for *P*. *falciparum* aldolase binding to cytoplasmic domains.

ProteinCTD	Residues	Gene	Sequence	K_d_ (μM)	S.D.
MTRAP	479–498	PF3D7_1028700	ALKGKDNKAMDEEEFWALE	17.0	5.0
MTRAP	452–498		YFLRKEKTEKVVQEETKEENFEVMFNDDALKGKDNKAMDEEEFWALE	13.8	3.5
TRAP	555–574	PF3D7_1335900	EDKDLDEPEQFRLPEENEWN	18.0	1.0
TRAP	530–574		YKFVVPGAATPYAGEPAPFDETLGEEDKDLDEPEQFRLPEENEWN	5.0	1.0
AMA1	555–562	PF3D7_1133400	VLMEKPYY	*	
AMA1	567–622		YKRKGNAEKYDKMDEPQDYGKSNSRNDEMLDPEASFWGEEKRASHTTPVLMEKPYY	53.0	6.0
RH1	2941–2971	PF3D7_0402300	CDNNKMDDKSTQKYGRNQEEVMEIFFDNDYI	18.0	5.0
RH1	2921–2971		KNNKQEYDKEQEKQQQNDFVCDNNKMDDKSTQKYGRNQEEVMEIFFDNDYI	6.1	1.3
RH2A	3089–3130	PF3D7_1335400	KTNSGDNNSNEINEAFEPNDDVLFKEKDEIIEITFNDNDSTI	*	
RH2B	3211–3254	PF3D7_1335300	DRSNKDECDFDMCEEVNNNDHLSNYADKEEIIEIVFDENEEKYF	125.0	20.0
RH4	1650–1716	PF3D7_0424200	KNSNEPHHIFNIFQKEFSEADNAHSEEKEEYLPVYFDEVEDEVEDEVEDEDENENEVENENEDFNDI	3.5	0.9
EBA140	1155–1210	PF3D7_1301600	RMGKSNEEYDIGESNIEATFEENNYLNKLSRIFNQEVQETNISDYSEYNYNEKNMY	83.0	10.0
EBA175	1445–1502	PF3D7_0731500	KYQSSEGVMNENNENNFLFEVTDNLDKLSNMFNQQVQETNINDFSEYHEDINDINFKK	96.0	13.0
EBA181	1511–1567	PF3D7_0102500	RKNLDDEKGFYDSNLNDSAFEYNNNKYNKLPYMFDQQINVVNSDLYSEGIYDDTTTF	130.0	20.0
PTRAMP	328–353	PF3D7_1218000	YHIFYKRKGAEKELYENVAGRYMYD	*	

The data are presented as a mean ± standard deviation (S.D.) from duplicate reactions

* no binding.

The short C-terminal peptides from both TRAP and MTRAP (20 and 19 amino acids, respectively) bound aldolase (K_d_ of 18 μM and 17 μM, respectively) somewhat less tightly than the full length CTDs (45 and 47 amino acid residues; K_d_ of 5 μM and 13.8 μM, respectively) as shown in Table 1. These results indicate that more residues in the CTD contribute to the binding to aldolase than just the C-terminal EENEWN or EEFWALE motifs studied previously [1, 27]. These additional interactions with aldolase are likely to be adjacent to and in addition to those within the enzyme’s active site. Full length MTRAP CTD produced as either a synthetic peptide or as a recombinant protein had a similar K_d_ value for aldolase binding. Full length variant MTRAP CTD sequences containing single amino acid substitutions of the tryptophan (Trp495Ala), or the two threonines (Thr459Asp, Thr459Val, Thr467Asp, and Thr467Val) showed no detectable binding to aldolase (data not shown), indicating that single amino acid variants can easily disrupt the protein-protein interaction.

The AMA1 CTD C-terminal eight amino acids (VLMEKPYY) did not bind aldolase, but the full length CTD bound with a K_d_ of 53 μM, consistent with the binding reported with rabbit muscle aldolase using a non-quantitative method [23]. Thus although AMA1 is able to bind aldolase the interaction is much weaker than that between MTRAP and aldolase.

The CTDs of RH and EBL family proteins varied in their ability to bind to aldolase. Within the RH family, the full length RH1 and RH4 CTDs bound tightly (6.1 μM and 3.5 μM, respectively), whereas the RH2B CTD bound weakly (125 μM) and RH2A CTD did not bind at all, confirming previous reports using rabbit muscle aldolase [24]. In the EBL family, the CTDs of EBA140 (83 μM), EBA175 (96 μM), and EBA181 (130 μM) all bound to aldolase, although relatively weakly with much lower binding affinities than those of the RH1/4 and TRAP protein families.

### Phosphorylation of MTRAP, AMA1, RH and EBL CTDs and its effect on their binding to aldolase

To examine whether or not phosphorylation affected the ability of the CTDs to bind aldolase, we first looked at their ability to be phosphorylated by serine/threonine kinases *in vitro*. Two kinases, CDPK1 and PKA, which have been implicated in phosphorylation of glideosome proteins, were chosen for this analysis (Table 2).

**Table 2:** CTD peptide phosphorylation by CDPK1 or PKA in vitro.

Protein CTD	CDPK1	PKA
MTRAP	+	+
AMA1	+	+
RH1	+	-
RH2A	-	-
RH2B	*	-
RH4	-	-
EBA140	+	-
EBA175	+	-
EBA181	+	-
PTRAMP	-	-

Phosphorylation (+) was confirmed by electrospray-mass spectrometry (S1 and S2 Figs).

* not done.

**Fig 1:**
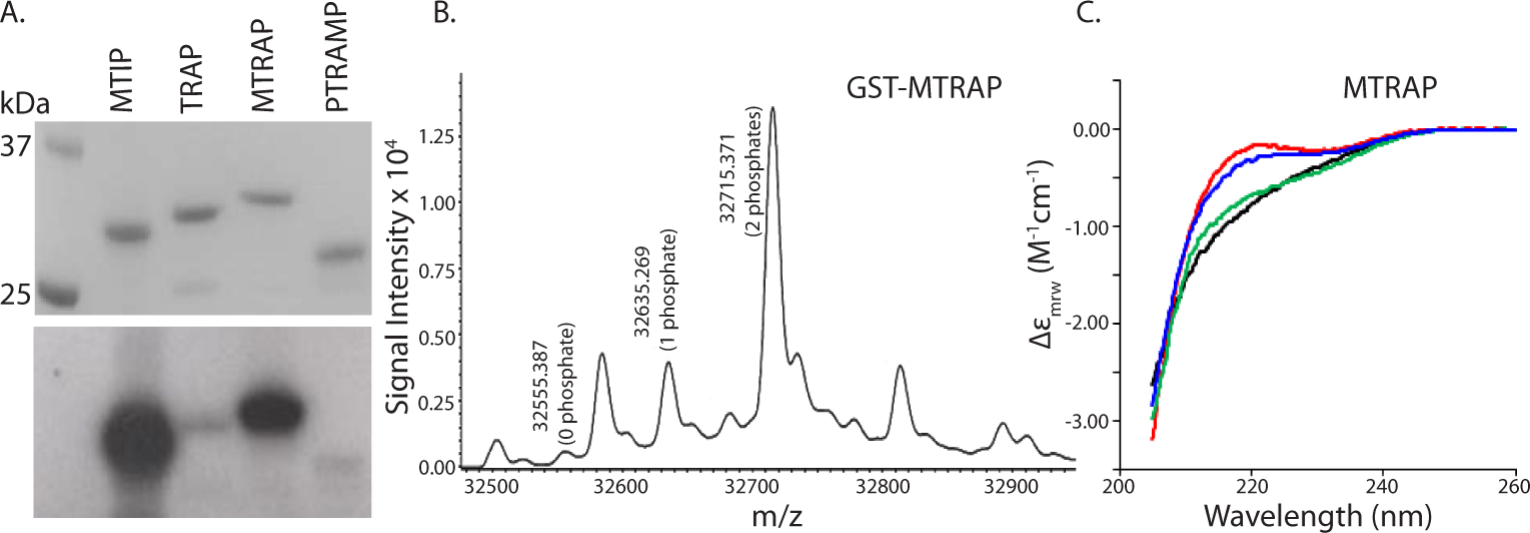
Phosphorylation of MTRAP CTD. A) CDPK1-mediated phosphorylation of recombinant CTDs fused to GST *in vitro*. MTIP and PTRAMP were used as positive and negative control substrates, respectively. The upper panel shows the proteins stained with Coomassie Blue staining and the lower panel shows ^32^P labelling detected by autoradiography resulting from protein phosphorylation. B) Analysis of the phosphorylated MTRAP-GST fusion protein by electrospray mass spectrometry, with the peaks corresponding to the protein and its phosphorylated forms identified. The unlabelled peaks at 32584.4 and 32813.6 correspond to the dual phosphorylated product without the terminal methionine and with an additional H_2_SO_4_ (a known electrospray artefact), respectively. C) Circular dichroism spectrum of MTRAP CTD (black), and the forms phosphorylated at either Thr-459 (blue), Thr-467 (green) residues or both residues (red). The CD absorption coefficient calculated on a mean residue weight basis (Δε_mrw_) is plotted against wavelength.

The phosphorylation of MTRAP and TRAP CTDs expressed as fusion proteins with GST was evaluated *in vitro* by ^32^P incorporation (Fig 1A) and by electrospray (ES) mass spectrometry and collision-induced dissociation (CID) fragmentation analysis. Only GST-MTRAP CTD was efficiently phosphorylated by CDPK1. ES-MS analysis of the unphosphorylated protein (32555.361 Da) and phosphorylated protein (two forms, 32635.269 Da and 32715.371 Da) indicated that the modified protein was an additional 80 and 160 Da in size, corresponding to incorporation of one or two phosphate groups, respectively (Fig 1B). CID analysis indicated that both Thr-459 and Thr-467 were phosphorylated in the MTRAP CTD (S1A Fig). MTRAP CTD produced as either a recombinant protein or as a synthetic peptide was also phosphorylated by PKA at the same two positions (S2A Fig). Far UV circular dichroism spectroscopy of synthetic peptides indicated that this modification did not have a significant effect on the secondary structure (Fig 1C). The spectra were typical of essentially unstructured (random coil) proteins, although the data suggest that phosphorylation of Thr-459 slightly changed the distribution of conformers present in this unfolded state. Using ES-MS and CID it was determined that both CDPK1 and PKA phosphorylated AMA1 at Ser610 (S1B and S2B Figs), the residue implicated in earlier studies [33]. The CTD of RH1 was phosphorylated by CDPK1 but not PKA (S2C Fig) only at either Ser-2950 or Thr-2951; the CID methodology was unable to identify with accuracy the phosphorylation site (S1C Fig). Neither RH2a nor RH4 CTD was phosphorylated by either kinase (Table 2, S2D Fig). CDPK1 but not PKA phosphorylated EBA140 (Table 2, S2E Fig), the exact sites of which were not determined (S1E Fig). CDPK1 phosphorylated EBA175 at Thr1483 and Ser 1489 (S1F and S2F Figs) and EBA181 at Ser1523 and Ser1528 (S1G and S2G Figs). These results show for the first time that the cytoplasmic tails of MTRAP, AMA1, RH1, EBA140, EBA175 and EBA181 can be phosphorylated by CDPK1 *in vitro*. MTRAP and AMA1 CTDs are also phosphorylated by PKA. CK2, which has been shown previously to phosphorylate RH4 [35] and RH2b at position Ser3233 [34], was found to phosphorylate none of the CTDs tested other than RH4 with one phosphate group at either position Ser1667 or Ser1674 (S1D and S2D Figs).

### Phosphorylation increases the affinity of certain CTDs for aldolase

**Fig 2:**
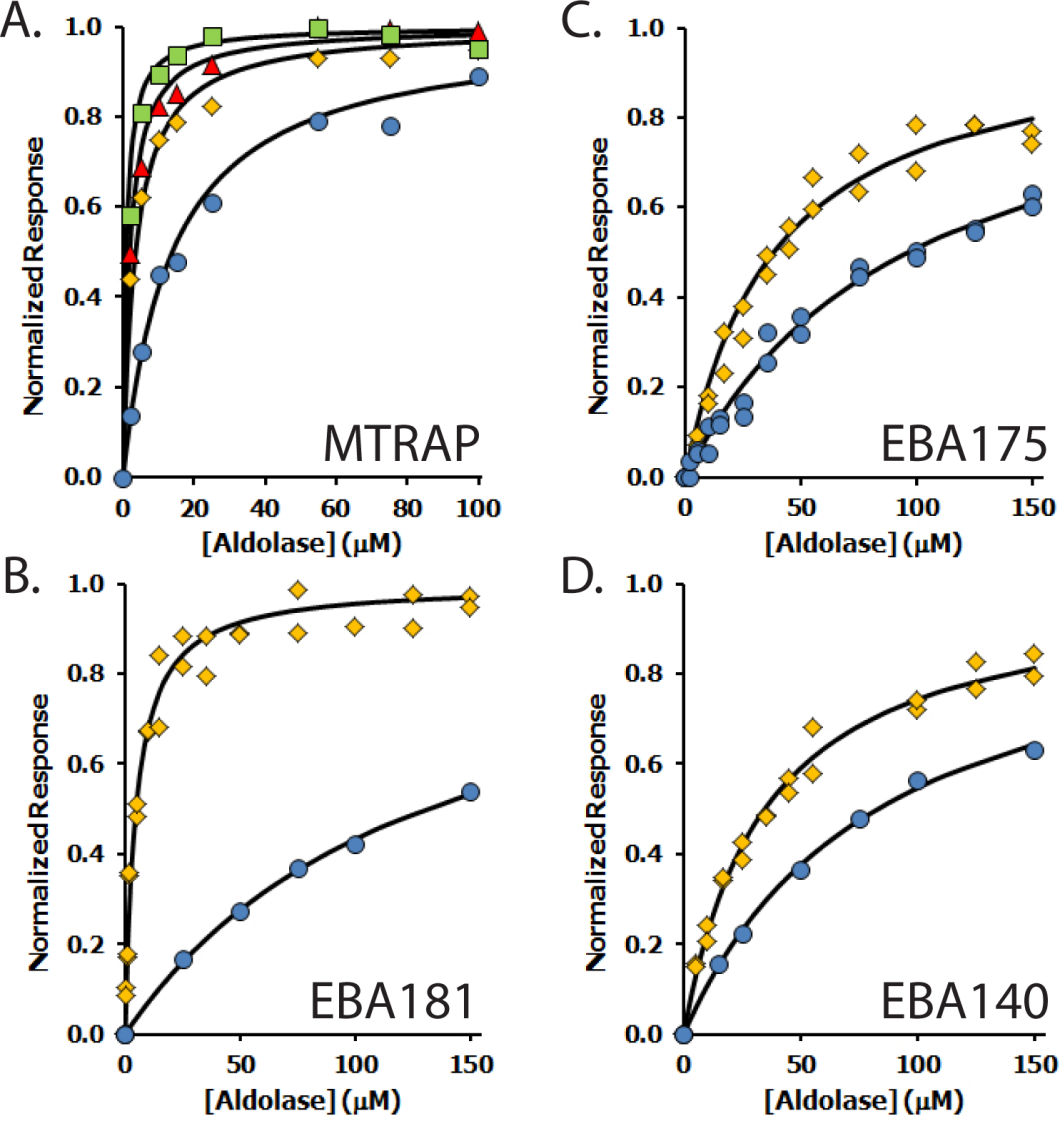
Binding of protein CTDs to aldolase measured by biolayer interferometry. A) Binding assays of aldolase to MTRAP CTD, either non-phosphorylated (blue circles) or phosphorylated at Thr-459 (yellow diamonds), Thr-467 (red triangles), and both Thr residues (green squares); B), C) and D) Binding of aldolase to either non-phosphorylated (blue circles) or phosphorylated (yellow diamonds) EBA181, EBA175 and EBA140, respectively. Data were obtained using the Octet Red system (ForteBio).

The effect of the CTD phosphorylation on aldolase binding was investigated using biolayer interferometry using phosphorylated synthetic peptides or peptides phosphorylated *in vitro* (Table 3). Overall the results were similar for both types of peptide and showed that the outcome of phosphorylation fell into three categories: either a substantial increase in affinity, a slight increase in affinity or no change in affinity relative to the unmodified sequence. Phosphorylation of MTRAP CTD substantially increased its binding to aldolase as shown by the lower K_d_ values compared with the unphosphorylated CTD, representing a greater than 10-fold increase in affinity of the fully phosphorylated form (Table 3 and Fig 2). Similarly, phosphorylation of RH2b and EBA181 CTDs resulted in large increases in affinity for aldolase: 11- and 28-fold respectively. The phosphorylation of AMA1 at Ser-610 resulted in a slightly increased binding to aldolase. Similarly there was a modest increase in affinity for aldolase following phosphorylation of RH1, EBA175, and EBA140 (around 2-fold in all three cases). The phosphorylation of RH4 CTD resulted in no increase in affinity for aldolase. No evidence had been obtained that RH2a can be phosphorylated and so it was not tested further.

**Table 3:** *P*. *falciparum* aldolase binding to phosphorylated cytoplasmic domains.

Protein CTD	K_d_ (μM)	S.D.
*MTRAP*		
unmodified	13.8	3.5
phospho T459	3.5	0.7
phospho T467	1.9	0.5
phospho T459 and T467	0.9	0.3
*AMA1*		
unmodified	53.0	6.0
phospho S610	44.0	8.0
*RH1*		
unmodified	6.1	1.3
phospho S2950 or T2951	2.5	0.9
*RH2B*		
unmodified	125.0	20.0
phospho S3233	11.0	3.5
*RH4*		
Unmodified	3.5	0.9
phospho S1667 or S1674	2.5	0.8
*EBA140*		
unmodified	83.0	10.0
phosphorylated	35.0	6.0
*EBA175*		
unmodified	96.0	13.0
phospho T1483 and S1489	38.0	5.0
*EBA181*		
unmodified	130.0	20.0
phospho S1523 and S1528	4.7	0.8

The data are presented as a mean dissociation constant with standard deviation from the mean calculated from duplicate reactions.

In summary, the phosphorylated forms of certain CTDs bind more strongly than the non-phosphorylated forms to aldolase. This indicates that the complex with aldolase is more stable when the C-terminal domains are phosphorylated, with a higher probability of formation, and greater stability once formed.

### MTRAP competes with AMA1 and RH4 for the binding to aldolase

**Fig 3:**
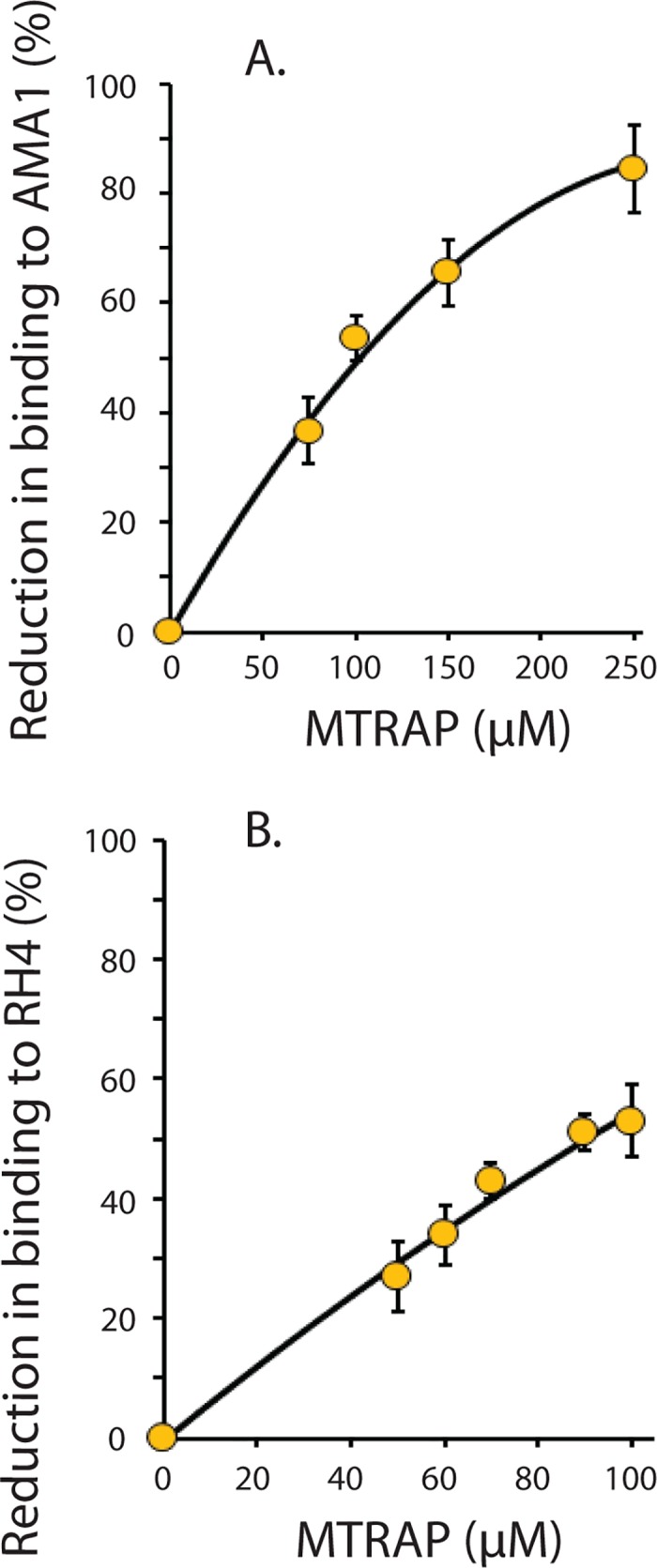
MTRAP competes with AMA1 and RH4 for binding to aldolase. (A) AMA1 was loaded onto the Octet Red sensor and incubated with 70 μM aldolase in the presence of increasing concentrations of MTRAP CTD peptide. (B) RH4 was loaded on the sensor and incubated with 20 μM aldolase in the presence of increasing concentrations of MTRAP CTD. The experiments were performed in duplicate and the mean values +/- S.D. are plotted on the graph as a percentage reduction in aldolase binding to AMA1 or RH4 at increasing concentrations of MTRAP CTD.

Having shown that various CTD peptides bind to aldolase it was important to determine whether or not the binding of one polypeptide competed with that of another. Therefore we evaluated the ability of MTRAP CTD to compete with AMA1 or RH4 CTD for binding to aldolase. Briefly, a fixed concentration of aldolase (either 70 μM or 20 μM), together with increasing concentrations of MTRAP CTD were applied to the streptavidin sensor chip loaded with biotinylated AMA1 or RH4 CTD. The results clearly showed that MTRAP competes with AMA1 for binding to aldolase (Fig 3A). Similarly, MTRAP was found to compete with RH4 for the binding to aldolase (Fig 3B), reducing the aldolase binding by 56% at a 5-fold molar excess of MTRAP to aldolase, indicating that these CTDs have overlapping aldolase binding sites.

### Evaluation of MTRAP phosphorylation in the parasite using the GFP-tagged protein

**Fig 4:**
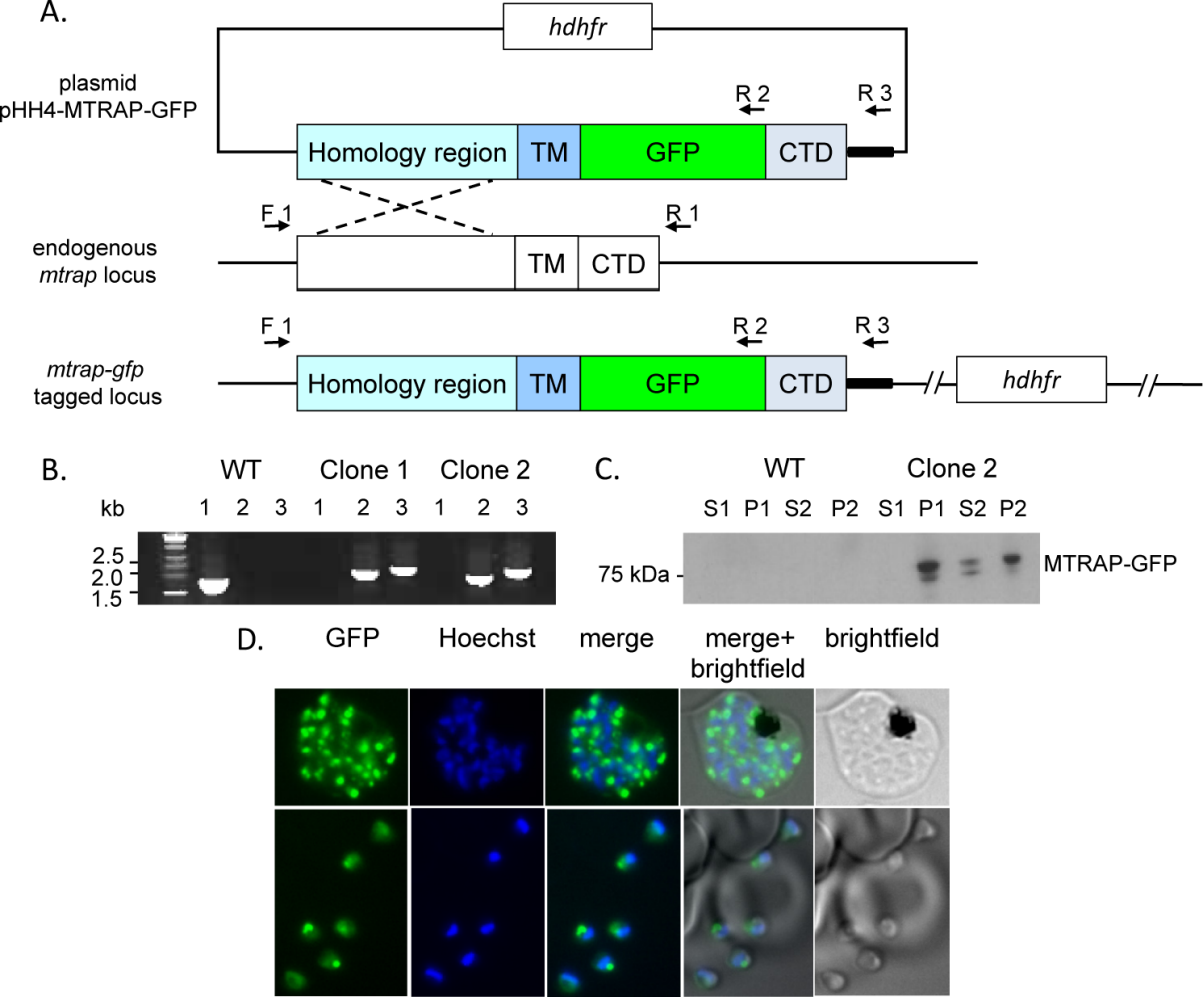
Expression of GFP-tagged MTRAP in *P*. *falciparum* asexual blood stage parasites. A. A schematic representation of the GFP-tagging of MTRAP by single crossover homologous recombination into the *mtrap* locus. The modified *mtrap* containing sequence coding for GFP inserted between the regions coding for the transmembrane (TM) and cytoplasmic domain (CTD) was cloned as the pHH4-MTRAP-GFP plasmid and transfected into parasites. The locations of PCR primers diagnostic of integration are indicated by arrows. B. PCR analysis of genomic DNA from wild type (WT) parasites and two clones with integrated sequence, using primers F1 and R1 (lane 1), F1 and R2 (lane 2) and F1 and R3 (lane 3); the expected size of the product is 1857, 2284 and 2510 bp, respectively. C. Western blot analysis with anti-GFP antibodies of lysates of schizonts prepared from WT or clone 2. Hypotonic buffer lysate was divided into soluble (S1) or insoluble (P1) fractions and a similar fractionation was performed on lysate prepared with a buffer containing NP40 detergent (S2 and P2, respectively). D. Fluorescent images of live parasites expressing GFP-tagged MTRAP (green); nuclei (blue) were stained with Hoechst dye prior to microscopy. Merged and bright-field images are also shown.

To examine whether or not MTRAP CTD phosphorylation can be detected *in vivo*, we expressed GFP-tagged MTRAP in the parasite (Fig 4); the protein was then affinity purified and analysed for the presence of the phosphorylated Thr residues in the CTD. The endogenous *mtrap* gene could be targeted and the *gfp* sequence inserted, leading to the production of GFP-tagged MTRAP in a subcellular location suggestive of an apical organelle. The full length protein was detected by western blotting with antibodies to the GFP-tag and cellular fractionation studies indicated that the protein was associated with the insoluble (membrane bound) fraction. Even in the presence of a non-ionic detergent (Nonidet P40) much of the protein remained insoluble (Fig 4). Detergent soluble protein was then purified using GFP-trap resin and analysed by tryptic digestion and mass spectrometry. Although peptides derived from MTRAP were identified, no evidence of MTRAP phosphorylation was obtained.

## Discussion

We have examined the ability of the CTDs of four groups of proteins to bind to *P*. *falciparum* aldolase using direct binding assays. The majority of the CTDs were able to bind to aldolase, however the affinity was broadly variable. We have also shown that some of these CTDs can be phosphorylated *in vitro* and that this posttranslational modification may enhance their binding to aldolase. In addition the results show that these CTDs bind to overlapping sites on aldolase. In the cell the availability, concentration, phosphorylation status and affinity of the individual CTDs will affect their interactions with aldolase.

In the current model of parasite motility and cell invasion, aldolase provides the direct link between the actomyosin motor that is located beneath the parasite plasma membrane and type 1 membrane proteins that are initially located in the microneme apical organelles and then are secreted onto the parasite surface. It has been proposed that the TRAP family of proteins mediate this process at different stages of the life-cycle: TRAP, which is implicated in sporozoite motility and cell invasion; CTRP in ookinete motility and crossing the mosquito gut epithelium, and MTRAP in merozoite invasion of erythrocytes [3, 7, 25, 44–47]. It had been shown previously that the last 6 amino acids of TRAP bind to aldolase and that aldolase binds to actin, providing a way for TRAP to engage with the actomyosin motor and provide a mechanism by which force generated by the motor is transmitted out of the cell to drive both sporozoite motility and cell invasion [27]. By analogy it has been proposed that MTRAP, expressed during blood stage schizogony in merozoite micronemes, carries out the same function in erythrocyte invasion [7]. Most recently Riglar and colleagues [48] concluded that MTRAP was unlikely to be important in RBC invasion, although it is possible that a tightly bound or phosphorylated protein might not be recognised by the antibodies used to locate the protein.

Other type 1 membrane proteins initially located in the micronemes have been implicated in host cell recognition and formation of the moving junction between parasite and host cell surface that accompanies invasion and successful establishment of the intracellular developmental stage inside a parasitophorous vacuole. These proteins may also have a signalling function, transducing external stimuli into signals mediated by the CTD [49]. At the asexual blood stage two families of proteins have been identified as erythrocyte binding proteins: the EBL family and the RH family and, with the exception of RH5, these are also type 1 membrane proteins [14–20]. AMA1 has also been implicated in the formation of the moving junction between parasite and host cell surface. During cell invasion the moving junction is thought to be moved by the parasite actomyosin motor to the rear, effectively driving the parasite into the developing parasitophorous vacuole [21, 22, 48]. However, the molecular linkage between motor and moving junction is unclear. Whether or not AMA1 and the EBL and RH families are coupled to the motor through aldolase is unclear. AMA1 was reported to bind to rabbit muscle aldolase using non-quantitative methodologies [23]. In addition, Pal-Bhowmick and colleagues also examined the binding of RH and EBL family CTDs to rabbit muscle aldolase [24]. Whilst their results on the relative binding of the RH proteins were in broad agreement with our results they did not detect any binding of EBL CTDs. It is possible that this was due their use of mammalian aldolase rather than parasite aldolase. Our measured K_d_ values with parasite aldolase differ substantially from those reported for rabbit muscle aldolase [24]. The differences in aldolase binding observed for MTRAP and TRAP suggest that even small differences in aldolase structure can affect the binding of these sequences.

Phosphorylation of the CTDs is likely to be important in their function. It has been shown that the AMA1 CTD is important in erythrocyte invasion and that substitution of phosphorylated Ser610 by alanine impaired invasion [33]. More recently, several phosphoproteome studies have identified at least four other Ser and Thr residues in the AMA1 CTD that are phosphorylated in schizonts *in vivo* [50, 51]. Similarly, RH2b was shown to be phosphorylated prior to host cell egress at Ser3233 in the CTD, though no role was identified related to this phosphorylation [34]. No phosphorylation of MTRAP or EBL and other RH family members' CTDs was detected in these phosphoproteome studies. We have shown that some but not all of the CTD examined are phosphorylated *in vitro* by either CDPK1 or PKA, two kinases that have been implicated in the phosphorylation of motor components in merozoites [32, 52]. Recently it was reported that the phosphorylation by CK2 of RH4 CTD residues we have identified is essential for erythrocyte invasion [35]. It is possible that other kinases fulfil the same role at other stages of the life-cycle, for example CDPK3 has been implicated in control of motor function in ookinetes and sporozoites and one prediction is that CDPK3 may phosphorylate TRAP and/or CTRP. Aldolase is also phosphorylated in the parasite [32, 53] but the significance of this modification for its interaction with CTDs is unknown.

Of particular interest is the finding that phosphorylation of all the CTDs increased their affinity to aldolase, with MTRAP CTD having the largest increase. This posttranslational modification provides a way to modulate the binding of these CTDs to aldolase and thus its assembly to the motor complex. However, we were unable to demonstrate phosphorylation of MTRAP purified from merozoites. The fact that phosphorylation of the CTDs of MTRAP, EBL and some RH family members has not been detected in the protein purified from parasites may reflect the fact that they are not modified in this way or there may be technical reasons around the detection methodology and its sensitivity. It is also possible that phosphorylation/dephosphorylation is a dynamic process and, for example, the MTRAP CTD may be transiently phosphorylated as the protein complex is assembled immediately prior to cell invasion and therefore not detectable by current methodology. Phosphorylation/dephosphorylation provides a dynamic dimension to the interaction of the CTDs with aldolase, which may be reflected in the organisation and function of the motor. In the light of the in vitro phosphorylation data it will be important to investigate this process further in vivo, for example by using phospho-peptide specific antibodies to detect the modification[53].

If the binding of AMA1, RH or EBL proteins to aldolase is an important interaction that modulates their function, it is important to note the wide range of affinities of the CTDs for aldolase. The RH and EBL proteins are involved in redundant pathways of invasion, binding different host cell ligands: for example RH4 binds CR1, and EBA175 binds glycophorin A. The efficiency of invasion mediated by particular RH or EBL proteins may depend not just on their affinity for host cell receptors but also on the properties of their respective CTDs, as proposed for RH2a and RH2B [54]. Such properties may include their affinity for aldolase, their potential to be phosphorylated/dephosphorylated and the impact of this post translational modification on protein-protein interactions.

## Materials and Methods

### Recombinant proteins and peptides

*P*. *falciparum* aldolase was prepared as described previously [42]. Full length TRAP, MTRAP, PTRAMP and AMA1 wild type and mutant CTD sequences were also expressed in recombinant form as a C-terminal fusion with GST and with or without a biotinylation sequence GPLGSMSGLNDIFEAQKIEWHE. GST-tags were cleaved using PreScission protease (GE Healthcare) and *in vitro* biotinylation was carried out at 30°C overnight using 1 μM biotin ligase (Avidity) and 40 μM of each protein to be biotinylated. The concentrations of the biotinylated proteins were determined by spectrophotometry and by electrospray-mass spectrometry analysis.

Synthetic peptides based on CTD sequences (shown in Table 1) were purchased from Biomatik, USA, either as unmodified peptides or with specific residues phosphorylated and with N-terminal biotinylation.

### Phosphorylation analysis

Phosphorylation assays were performed using 10 μM recombinant CDPK1 [37], PKA (Promega), or CK2 (New England Biolabs) in kinase buffer (20 mM Tris-HCl pH 8.0, 20 mM MgCl_2_, with or without 1 mM CaCl_2_) and 1 mM ATP at 30°C for 3 hours with 100 μM substrate protein. Products were analysed by mass spectrometry and used in binding assays (see below). Alternatively 0.185 MBq [γ^32^P] ATP was added to 10μl 1mM ATP, and 2μl of this mixture added to each sample to initiate the kinase reaction. Following incubation at 30°C for 3 hours, samples were mixed with SDS-PAGE loading buffer (1:1 vol/vol), and heated to 90°C for 5 minutes. Each sample was loaded in two halves on a 10% Bis-Tris NuPAGE gel using MOPS running buffer. The gel was fixed, dried and exposed to Kodak Biomax MR film to visualize the radiolabelled proteins, or the proteins were visualised by Coomassie Brilliant Blue staining to ensure that equivalent amounts of protein were loaded.

### Peptide analysis by mass spectrometry

Non-phosphorylated, phosphorylated and biotinylated polypeptides were analysed by electrospray (ES)-mass spectrometry and collision-induced dissociation (CID) fragmentation pathway analysis. The samples (100 pmol) were first desalted using P10 ZipTip® Pipette Tips (Millipore) containing either C18 or C4 according to the size of the protein to be desalted. For CID analysis, the eluted proteins in 70% (vol/vol) acetonitrile (ACN) and 0.1% (vol/vol) acetic acid were injected into the mass spectrometer using a 10 μl min^-1^ flow rate. Phosphorylation sites were determined using stand-alone nanospray on an LTQ Orbitrap Velos (Thermo Fisher Scientific). Nanospray needles (“Medium” ES380, Thermo Fisher Scientific) were operated at 1.6 kV and collision induced dissociation MS/MS was performed in the ion trap and recorded in the Orbitrap. Spectra were acquired over a collision energy range of 20-30eV at a resolution of 100k. The averaged MS/MS spectrum was then deconvoluted using Xtract and interpreted using Prosight (https://prosightptm.northwestern.edu/).

For protein analysis, peptides were generated by in-gel or in-solution digestion using sequencing-grade modified endopeptidases, trypsin, Asp-N or chymotrypsin (Promega). The in-gel digestion was performed after first resolving proteins by SDS-PAGE and subsequent staining using the Colloidal Blue staining kit (Invitrogen), according to the manufacturer’s instructions. The proteins bands were excised and both SDS and stain removed with 100% (vol/vol) ACN, and 5 mM ammonium bicarbonate containing 50% (vol/vol) ACN, for 30 min at room temperature. The proteins were then reduced using 20 mM DTT for 1 hour and alkylated with 5 mM iodoacetamide for 20 min at room temperature. The gel slices were first dehydrated in 100% (vol/vol) ACN and then the proteins digested in 20 μl final volume in 5 mM ammonium bicarbonate using 2 ng μl^-1^ of endopeptidase, overnight at 32°C. Digestion in solution was performed in a 20 μl final reaction volume with a molar ratio of 1:100 enzyme:protein in 5 mM ammonium bicarbonate, with overnight digestion at 32°C. One to ten microlitres of peptide solution acidified to a final concentration of 0.1% TFA was loaded at 5 μl min^-1^ onto a 2 mm x 100 μm Acclaim Pepmap C18 trap column attached to an Ultimate 3000 nanoRSLC HPLC (Thermo Scientific), then peptides were eluted at 0.22 μl min^-1^ through a 50 cm x 75 μm Acclaim Pepmap C18 column. Starting with Buffer A (2% ACN, 0.1% formic acid), a gradient from 9% to 25% buffer B (80% ACN, 0.1% formic acid) over 37 minutes, and 25% to 40% buffer B over 18 minutes was used; followed by a short gradient to 100% B. Eluant was introduced into a LTQ Orbitrap Velos Pro (Thermo Scientific) using a Proxeon NanoES source (Thermo Scientific) and a 30 μm ID stainless steel emitter operated at 2 kV. The Orbitrap was operated in “Data Dependent Acquisition” mode with a survey scan at a resolution of 60,000 from m/z 300–1500, followed by MS/MS in the LTQ of the top 6–12 ions. Dynamic exclusion was used with a time window of 40 s. The Orbitrap charge capacity was set to a maximum of 10^6^ ions in 10 ms, whilst the LTQ was set to 10^4^ ions in 100 ms. Raw files were processed using Proteome Discoverer (PD) 1.3 (Thermo Scientific) with Mascot 2.4 (Matrix Science, UK) as the search engine against the appropriate protein fasta database. A decoy database of reversed sequences was used to filter the results at a false detection rate of 1%. Label free quantitation was achieved using the precursor-ion quantitation module of PD.

### Circular Dichroism (CD) spectroscopy

Far-UV CD spectra were recorded on a Jasco J-715 spectropolarimeter fitted with a cell holder thermostatted by a PTC-348 Peltier unit. All CD measurements were made in 20 mM Tris (pH 8) at 20°C using fused silica cuvettes with 1 mm path length (Hellma, Jena, Germany). The spectra were typically recorded with 0.1 nm resolution and baseline corrected by subtraction of the appropriate buffer spectrum. CD intensities are presented as the CD absorption coefficient calculated on a mean residue weight basis (Δε_mrw_) Secondary structure content was estimated using methods described elsewhere [55].

### Binding assays using biolayer interferometry

Protein interactions were analysed by biolayer interferometry using the Octet Red system (ForteBio) at 25°C in 96-well microplates, as described previously [42]. All assays were performed in 100 mM Tris-HCl pH 8.0, according to the manufacturer’s instructions. A baseline was established over 1 min, and biotinylated samples were loaded onto streptavidin sensors for 1 min. The sensors were washed for 1 min and then incubated with aldolase at different concentrations (0.05–250 μM) for 1 min, followed by a dissociation phase over 5 min. The experiments were performed in duplicate or triplicate depending on the experiment. The data were analysed as described previously [42].

For competition experiments, for example of MTRAP in the interaction between AMA1 or RH4 and aldolase, the Octet Red system was also used. Biotinylated AMA1 or RH4 peptide was loaded onto the streptavidin coated sensor and following washing incubated with 20 or 70 μM aldolase with (or without) increasing concentrations of unlabelled competitor MTRAP CTD peptide. Following incubation the data were collected and analysed as described above. The experiments were performed in duplicate.

### Transgenic parasites expressing GFP-tagged MTRAP—genetic modification of *P*. *falciparum* 3D7

Sequence coding for *P*. *falciparum* MTRAP with a GFP tag situated distal to the transmembrane (TM) sequence and proximal to the CTD was placed into the pHH4 vector [56] for insertion into the endogenous *mtrap* locus in the parasite by homologous recombination (see Fig 4). The region of homology was amplified by PCR from genomic DNA with the primers AAACCCGGGTCGAAAAATGCTAGAG GTTATAGAGG (forward) and GGCCTAGGTTTTTCTTTACGTAAGAAATAG (reverse) to incorporate XmaI and AvrII restriction enzyme sites (underlined). The sequence coding for MTRAP CTD was synthesized in a recodonised form: TATTTTCTGCGCAAAGAAAAAACCGAAAAAGTGGTGCAGGAAGAAACCAAAGAAGAAAATTTTGAAGTTATGTTTAATGATGATGCCCTGAAAGGCAAAGATAATAAAGCCATGGATGAAGAAGAATTTTGGGCACTGGAATAA (*GeneArt*®). The GFP sequence was obtained from the pHH4 plasmid and fused to MTRAP CTD sequence by PCR amplification using the primers: CCCCTAGGAGTAAAGGAGAAGAACTTTTCAC, TTTTTTCTTTGCGTTTG TATAGTTCATCCATGCCATGTGTAATCCC, and GGCCGCGGTTATTCCAGTGCCC to introduce AvrII and SacII restriction enzyme sites. These DNA fragments were then incorporated upstream of the *P*. *berghei dhfr* 3’ UTR in the pHH4 plasmid and Sanger sequencing (Beckman Genomics) was used to confirm the correct sequence assembly.

To incorporate the modified gene, *P*. *falciparum* 3D7 ring stage parasites were transfected with 100 μg of plasmid DNA and cultured continuously under a WR99210 (2.5 nM) drug selection cycle for 4 cycles over 12 weeks. After each cycle parasite cultures were examined for genomic integration by PCR and for GFP expression by fluorescence microscopy. PCR was performed with the following primers: F1 (GTGGGAGAGACATTTAAGAAGTAATA) and R1 (CTCGGTGGGGCTTTAAATAATG) targeting sequences upstream and downstream of endogenous *mtrap*, F1 and R2 (TTTGTATAGTTCATCCATGCCATGTGTAATCCC, part of the GFP sequence), and F1 and R3 (ATGCACAAAAAAAAATATGCACACAACATACACA, part of the *pbdhfr* 3’ UTR). Following detection of integration the parasites were cloned by limiting dilution. A schematic representation of the parasite integration, diagnostic PCR and western blot is presented in Fig 4.

### Analysis of recombinant *P*. *falciparum* cultured in vitro

*P*. *falciparum* asexual blood stages were cultured in human RBCs, and analysed by fluorescence microscopy and western blotting, using preparations of schizonts and merozoites purified as described previously [53]. GFP-tagged MTRAP was purified by GFP-trap chromatography and the bound proteins analysed by mass spectrometry as described [56].

## Supporting Information

S1 Fig Analysis of protein phosphorylation using tandem mass spectrometry. **Phosphorylation sites were interpreted from the mass data using either Prosight (https://prosightptm.northwestern.edu/) or Proteome Discoverer (PD) 1.3 (Thermo Scientific).** A. MTRAP phosphorylation (MTRAP_1 to MTRAP_6) interpreted using Prosight. There are only two potential phosphorylation sites and each of these threonines can be phosphorylated. B. AMA1 phosphorylation (AMA1_1 to AMA_5) interpreted using PD1.3. Phosphorylation was by CDPK1 (AMA1_1 to AMA1_3) and PKA (AMA1_4 and AMA1_5).The data are consistent with phosphorylation of Ser610 and not of the other 6 serine/threonine residues in the sequence. C. RH1 phosphorylation by CDPK1 interpreted using Prosight. The method did not resolve whether Ser2950 or Thr2951 was phosphorylated. D. RH4 phosphorylation by CK2 (RH4_1 to RH4_7) interpreted using PD1.3. The data are consistent with phosphorylation of either Ser1667 or Ser1674 with a very small amount of phosphorylation of both residues. E. EBA140 phosphorylation by CDPK1 (EBA140_1 to EBA140_2) interpreted using Prosight. No phosphorylated residues were identified unambiguously. F. EBA175 phosphorylation by CDPK1 (EBA177_1 to EBA175_2) interpreted using Prosight. Phosphorylation of both Thr1483 and Ser1489 was detected. G. EBA181phosphorylation by CDPK1 (EBA181_1 to EBA181_21) interpreted using PD1.3. Phosphorylation of both Ser1523 and Ser1528 was detected.
(PDF)

S2 Fig Phosphorylation in vitro of the cytoplasmic domain of proteins by CDPK1, protein kinase A and casein kinase 2, analysed by electrospray mass spectrometry. A. Phosphorylation of recombinant MTRAP peptide by protein kinase A. The MTRAP peptide (GPLGSMSGLNDIFEAQKIEWHEYFLRKEKTEKVVQEETKEENFEVMFNDDALKGKDNKAMDEEEFWALE; 8171.8) was biotinylated (8558.81; upper panel) and then phosphorylated using PKA (lower panel). The peaks corresponding to the protein and its phosphorylated forms are indicated. B. Phosphorylation of synthetic biotinylated AMA1 peptide by CDPK1 (upper panel) and protein kinase A (lower panel). The non-phosphorylated peptide (Biotin-YKRKGNAEKYDKMDEPQDYGKSNSRNDEMLDPEASFWGEEKRASHTTPVLMEKPYY, 6888.2) and a single phosphorylated form (6968.4) were detected. C. Phosphorylation of synthetic biotinylated RH1 peptide by CDPK1 (upper panel) and protein kinase A (lower panel). Only the non-phosphorylated peptide (Biotin-GKNNKQEYDKEQEKQQQNDFVCDNNKMDDKSTQKYGRNQEEVMEISFDNDYI, 6539.9) was detected after incubation with PKA and an additional single (6619.9) phosphorylated form was detected following incubation with CDPK1. D. Phosphorylation of synthetic biotinylated RH4 peptide by CDPK1 (upper panel), protein kinase A (middle panel) and casein kinase 2 (lower panel). Only the non-phosphorylated peptide (Biotin-KNSNEPHHIFNIFQKEFSEADNAHSEEKEEYLPVYFDEVEDEVEDEVEDEDENENEVENENEDFNDI, 8305.5) was detected after incubation with CDPK1 or PKA and a single (8386.2) phosphorylated form was detected following incubation with CK2. E. Phosphorylation of synthetic biotinylated EBA140 peptide by CDPK1 (upper panel) and protein kinase A (lower panel). Only the non-phosphorylated peptide (Biotin-RMGKSNEEYDIGESNIEATFEENNYLNKLSRIFNQEVQETNISDYSEYNYNEKNMY, 6995.8) was detected after incubation with PKA. F. Phosphorylation of synthetic biotinylated EBA175 peptide by CDPK1 (upper panel) and protein kinase A (lower panel). Only the non-phosphorylated peptide (Biotin-KYQSSEGVMNENNENNFLFEVTDNLDKLSNMFNQQVQETNINDFSEYHEDINDINFKK, 7163.2) was detected after incubation with PKA and a single (7243.2) phosphorylated form was detected following incubation with CDPK1. G. Phosphorylation of synthetic biotinylated EBA181 peptide by CDPK1 (upper panel) and protein kinase A (lower panel). Only the non-phosphorylated peptide (Biotin-RKNLDDEKGFYDSNLNDSAFEYNNNKYNKLPYMFDQQINVVNSDLYSEGIYDDTTTF, 7014.0) was detected after incubation with PKA and single (7094.9) and dual (7173.9) phosphorylated forms were detected following incubation with CDPK1.
(PDF)
