# Supplementary material for: The Binding of Plasmodium falciparum Adhesins and Erythrocyte Invasion Proteins to Aldolase Is Enhanced by Phosphorylation
Source: PLoS One. 2016 Sep 8;11(9):e0161850. doi: 10.1371/journal.pone.0161850 (PMC5015959; doi:10.1371/journal.pone.0161850)
Supplement: S1 Fig — Phosphorylation sites were interpreted from the mass data using either Prosight (https://prosightptm.northwestern.edu/) or Proteome Discoverer (PD) 1.3 (Thermo Scientific). A. MTRAP phosphorylation (MTRAP_1 to MTRAP_6) interpreted using Prosight. There are only two potential phosphorylation sites and each of these threonines can be phosphorylated. B. AMA1 phosphorylation (AMA1_1 to AMA_5) interpreted using PD1.3. Phosphorylation was by CDPK1 (AMA1_1 to AMA1_3) and PKA (AMA1_4 and AMA1_5).The data are consistent with phosphorylation of Ser610 and not of the other 6 serine/threonine residues in the sequence. C. RH1 phosphorylation by CDPK1 interpreted using Prosight. The method did not resolve whether Ser2950 or Thr2951 was phosphorylated. D. RH4 phosphorylation by CK2 (RH4_1 to RH4_7) interpreted using PD1.3. The data are consistent with phosphorylation of either Ser1667 or Ser1674 with a very small amount of phosphorylation of both residues. E. EBA140 phosphorylation by CDPK1 (EBA140_1 to EBA140_2) interpreted using Prosight. No phosphorylated residues were identified unambiguously. F. EBA175 phosphorylation by CDPK1 (EBA177_1 to EBA175_2) interpreted using Prosight. Phosphorylation of both Thr1483 and Ser1489 was detected. G. EBA181phosphorylation by CDPK1 (EBA181_1 to EBA181_21) interpreted using PD1.3. Phosphorylation of both Ser1523 and Ser1528 was detected. (PDF) [file pone.0161850.s001.pdf]

Supporting Information Figure 1.

Analysis of protein phosphorylation using tandem mass spectrometry. Phosphorylation sites were interpreted from the mass data using either Prosight (https://prosightptm.northwestern.edu/) or Proteome Discoverer (PD) 1.3 (Thermo Scientific).

A. MTRAP phosphorylation (MTRAP_1 to MTRAP_6) interpreted using Prosight. There are only two potential phosphorylation sites and each of these threonines can be phosphorylated.

B. AMA1 phosphorylation (AMA1_1 to AMA_5) interpreted using PD1.3. Phosphorylation was by CDPK1 (AMA1_1 to AMA1_3) and PKA (AMA1_4 and AMA1_5).The data are consistent with phosphorylation of Ser610 and not of the other 6 serine/threonine residues in the sequence.

C. RH1 phosphorylation by CDPK1 interpreted using Prosight. The method did not resolve whether Ser2950 or Thr2951was phosphorylated.

D. RH4 phosphorylation by CK2 (RH4_1 to RH4_7) interpreted using PD1.3. The data are consistent with phosphorylation of either Ser1667 or Ser1674 with a very small amount of phosphorylation of both residues.

E. EBA140 phosphorylation by CDPK1 (EBA140_1 to EBA140_2) interpreted using Prosight. No phosphorylated residues were identified unambiguously.

F. EBA175 phosphorylation by CDPK1 (EBA177_1 to EBA175_2) interpreted using Prosight. Phosphorylation of both Thr1483 and Ser1489 was detected.

G. EBA181phosphorylation by CDPK1 (EBA181_1 to EBA181_21) interpreted using PD1.3. Phosphorylation of both Ser1523 and Ser1528 was detected

A. MTRAP_1

```
[226.078]YFLRKEK[79.96633]TEKVVQEETKEENFEVMFNDDALKGKDNKAMDEEEFWALE
      Number of Amino Acids:  47
           Theoretical Mass:  6034.77 Da
            Mass Difference:  0.0609 Da
B Ions:  35     Y Ions:  8
```

Ion	Observed Mass (Da)	Theoretical Mass (Da)	Mass Error (Da)	Mass Error (PPM)	Delta M
Y46	5645.5626	5645.6278	-0.0652	-11.54	--
	5645.5493	5645.6278	-0.0785	-13.90	--
Y26	3073.4180	3073.3704	0.0476	15.50	--
Y25	2944.3683	2944.3278	0.0405	13.77	--
Y22	2567.1592	2567.1505	0.0087	3.40	--
Y5	664.3210	664.3240	-0.0030	-4.49	--
Y4	517.2528	517.2556	-0.0028	-5.41	--
Y3	331.1738	331.1763	-0.0025	-7.55	--
	331.1735	331.1763	-0.0027	-8.28	--
Y2	260.1367	260.1391	-0.0024	-9.34	--
	260.1365	260.1391	-0.0027	-10.29	--
B45	5774.6272	5774.6300	-0.0028	-0.48	--
B44	5703.5783	5703.5928	-0.0145	-2.54	--
B43	5517.5047	5517.5135	-0.0088	-1.60	--
B42	5370.4380	5370.4451	-0.0071	-1.33	--
B41	5241.4038	5241.4025	0.0012	0.23	--
B40	5112.3781	5112.3599	0.0182	3.55	--
B39	4983.3106	4983.3174	-0.0067	-1.35	--
B38	4868.2878	4868.2904	-0.0026	-0.54	--
B36	4666.2338	4666.2128	0.0210	4.50	--
B34	4424.0744	4424.0749	-0.0005	-0.11	--
B30	3995.8300	3995.8366	-0.0066	-1.66	--
B28	3811.6971	3811.7154	-0.0183	-4.81	--
B27	3696.6807	3696.6885	-0.0078	-2.11	--
B24	3320.5446	3320.5502	-0.0056	-1.68	--
B23	3189.5053	3189.5097	-0.0044	-1.39	--
B22	3090.4345	3090.4413	-0.0069	-2.22	--
B21	2961.3920	2961.3987	-0.0068	-2.28	--
B20	2814.3224	2814.3303	-0.0079	-2.79	--
B19	2700.2838	2700.2874	-0.0036	-1.32	--
B18	2571.2390	2571.2448	-0.0058	-2.25	--
	2571.2390	2571.2448	-0.0058	-2.24	--
B17	2442.2009	2442.2022	-0.0013	-0.52	--
	2442.2013	2442.2022	-0.0009	-0.35	--
B16	2314.1012	2314.1072	-0.0060	-2.60	--
	2314.1043	2314.1072	-0.0029	-1.27	--
B15	2213.0549	2213.0596	-0.0046	-2.09	--
	2213.0557	2213.0596	-0.0039	-1.76	--
B14	2084.0114	2084.0170	-0.0056	-2.69	--
	2084.0106	2084.0170	-0.0064	-3.08	--
B13	1954.9682	1954.9744	-0.0062	-3.18	--
B12	1826.9123	1826.9158	-0.0035	-1.91	--
	1826.9120	1826.9158	-0.0038	-2.08	--
B11	1727.8453	1727.8474	-0.0021	-1.24	--
B10	1628.7739	1628.7790	-0.0051	-3.15	--
B9	1500.6799	1500.6840	-0.0041	-2.73	--
	1500.6782	1500.6840	-0.0058	-3.86	--
B8	1371.6417	1371.6414	0.0003	0.19	--
B7	1190.6246	1190.6274	-0.0028	-2.39	--
	1190.6241	1190.6274	-0.0033	-2.81	--
B6	1062.5292	1062.5325	-0.0032	-3.06	--
B5	933.4872	933.4899	-0.0026	-2.81	--
B3	649.2913	649.2938	-0.0025	-3.90	--
B2	536.2080	536.2097	-0.0018	-3.32	--

Crude PScore: 3.36318e-31
McLuckey Score: 15.8284

Graphical Fragment Mapper

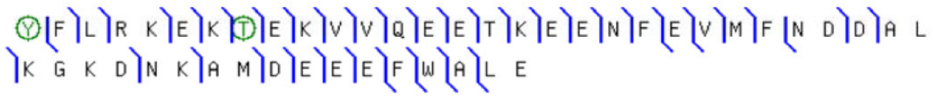

A. MTRAP_2

```
[226.078]YFLRKEK[79.96633]TEKVVQEETKEENFEVMFNDDALKGKDNKAMDEEEFWALE
      Number of Amino Acids:  47
           Theoretical Mass:  6034.77 Da
            Mass Difference:  0.0609 Da
B Ions:  28      Y Ions:  8
```

Ion	Observed Mass (Da)	Theoretical Mass (Da)	Mass Error (Da)	Mass Error (PPM)	Delta M
Y46	5645.5626	5645.6278	-0.0652	-11.54	--
	5645.5493	5645.6278	-0.0785	-13.90	--
Y26	3073.4180	3073.3704	0.0476	15.50	--
Y25	2944.3683	2944.3278	0.0405	13.77	--
Y22	2567.1592	2567.1505	0.0087	3.40	--
Y5	664.3210	664.3240	-0.0030	-4.49	--
Y4	517.2528	517.2556	-0.0028	-5.41	--
Y3	331.1738	331.1763	-0.0025	-7.55	--
Y2	260.1367	260.1391	-0.0024	-9.34	--
B45	5774.6272	5774.6300	-0.0028	-0.48	--
B44	5703.5783	5703.5928	-0.0145	-2.54	--
B43	5517.5047	5517.5135	-0.0088	-1.60	--
B42	5370.4380	5370.4451	-0.0071	-1.33	--
B41	5241.4038	5241.4025	0.0012	0.23	--
B40	5112.3781	5112.3599	0.0182	3.55	--
B39	4983.3106	4983.3174	-0.0067	-1.35	--
B38	4868.2878	4868.2904	-0.0026	-0.54	--
B36	4666.2338	4666.2128	0.0210	4.50	--
B34	4424.0744	4424.0749	-0.0005	-0.11	--
B30	3995.8300	3995.8366	-0.0066	-1.66	--
B28	3811.6971	3811.7154	-0.0183	-4.81	--
B27	3696.6807	3696.6885	-0.0078	-2.11	--
B24	3320.5446	3320.5502	-0.0056	-1.68	--
B23	3189.5053	3189.5097	-0.0044	-1.39	--
B22	3090.4345	3090.4413	-0.0069	-2.22	--
B21	2961.3920	2961.3987	-0.0068	-2.28	--
B19	2700.2838	2700.2874	-0.0036	-1.32	--
B18	2571.2390	2571.2448	-0.0058	-2.25	--
B17	2442.2009	2442.2022	-0.0013	-0.52	--
B16	2314.1012	2314.1072	-0.0060	-2.60	--
B15	2213.0549	2213.0596	-0.0046	-2.09	--
B14	2084.0114	2084.0170	-0.0056	-2.69	--
B12	1826.9123	1826.9158	-0.0035	-1.91	--
B11	1727.8453	1727.8474	-0.0021	-1.24	--
B9	1500.6799	1500.6840	-0.0041	-2.73	--
B8	1371.6417	1371.6414	0.0003	0.19	--
B7	1190.6246	1190.6274	-0.0028	-2.39	--

```
Crude PScore:  3.42849e-37
McLuckey Score: 26.8382
```

Graphical Fragment Mapper

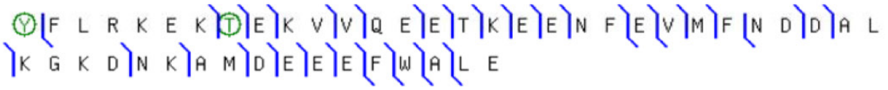

A. MTRAP_3

```
[226.078]YFLRKEK[79.96633]TEKVVQEETKEENFEVMFNDDALKGKDNKAMDEEEFWALE
      Number of Amino Acids:  47
           Theoretical Mass:  6034.77 Da
            Mass Difference:  0.0309 Da
C Ions:  10      Z Ions:  1
```

Ion	Observed Mass (Da)	Theoretical Mass (Da)	Mass Error (Da)	Mass Error (PPM)	Delta M
Z21	2437.0796	2437.0869	-0.0073	-3.00	--
C16	2331.1272	2331.1336	-0.0064	-2.75	--
C15	2230.0751	2230.0859	-0.0108	-4.84	--
C14	2101.0358	2101.0433	-0.0075	-3.58	--
C13	1971.9981	1972.0007	-0.0026	-1.33	--
C12	1843.9324	1843.9421	-0.0097	-5.27	--
C9	1517.7044	1517.7104	-0.0059	-3.91	--
C7	1207.6495	1207.6538	-0.0042	-3.50	--
C6	1079.5554	1079.5588	-0.0034	-3.15	--
C5	950.5126	950.5162	-0.0037	-3.85	--
C4	822.4183	822.4213	-0.0030	-3.60	--

```
Crude PScore:  1.53654e-11
McLuckey Score: 22.1053
```

Graphical Fragment Mapper

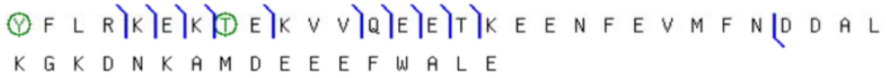

A. MTRAP_4

[226.078]YFLRKEKTEKVVQEE[79.96633]TKEENFEVMFNDDALKGKDNKAMDEEEFWALE
Number of Amino Acids: 47
Theoretical Mass: 6034.77 Da
Mass Difference: 0.0609 Da
B Ions: 16 Y Ions: 2

Ion	Observed Mass (Da)	Theoretical Mass (Da)	Mass Error (Da)	Mass Error (PPM)	Delta M
Y3	331.1735	331.1763	-0.0027	-8.28	--
Y2	260.1365	260.1391	-0.0027	-10.29	--
B20	2814.3224	2814.3303	-0.0079	-2.79	--
B18	2571.2390	2571.2448	-0.0058	-2.24	--
B17	2442.2013	2442.2022	-0.0009	-0.35	--
B16	2314.1043	2314.1072	-0.0029	-1.27	--
B15	2133.0887	2133.0932	-0.0045	-2.13	--
B14	2004.0448	2004.0506	-0.0058	-2.91	--
B13	1875.0013	1875.0080	-0.0067	-3.60	--
B12	1746.9501	1746.9495	0.0006	0.35	--
B11	1647.8758	1647.8811	-0.0053	-3.19	--
B10	1548.8074	1548.8126	-0.0053	-3.39	--
B9	1420.7125	1420.7177	-0.0052	-3.69	--
B7	1190.6241	1190.6274	-0.0033	-2.81	--
B6	1062.5292	1062.5325	-0.0032	-3.06	--
B5	933.4872	933.4899	-0.0026	-2.81	--
B3	649.2913	649.2938	-0.0025	-3.90	--
B2	536.2080	536.2097	-0.0018	-3.32	--

Crude PScore: 8.18535e-16
McLuckey Score: 8.66337

Graphical Fragment Mapper

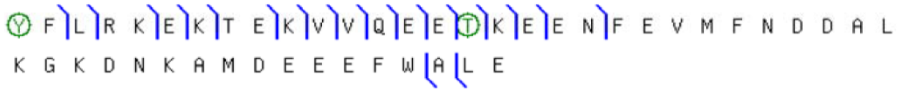

A. MTRAP_5

[226.078]YFLRKEKTEKVVQEE[79.96633]TKEENFEVMFNDDALKGKDNKAMDEEEFWALE
Number of Amino Acids: 47
Theoretical Mass: 6034.77 Da
Mass Difference: 0.0309 Da
C Ions: 5 Z Ions: 1

Ion	Observed Mass (Da)	Theoretical Mass (Da)	Mass Error (Da)	Mass Error (PPM)	Delta M
Z21	2437.0796	2437.0869	-0.0073	-3.00	--
C16	2331.1272	2331.1336	-0.0064	-2.75	--
C7	1207.6495	1207.6538	-0.0042	-3.50	--
C6	1079.5554	1079.5588	-0.0034	-3.15	--
C5	950.5126	950.5162	-0.0037	-3.85	--
C4	822.4183	822.4213	-0.0030	-3.60	--

Crude PScore: 2.03471e-05
McLuckey Score: 13.6842

Graphical Fragment Mapper

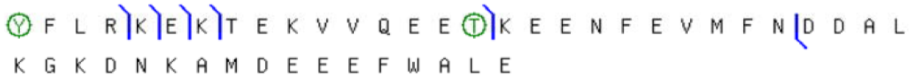

A. MTRAP_6

[226.078]YFLRKEKTEKVVQEE[79.96633]TKEENFEVMFNDDALKGKDNKAMDEEEFWALE
Number of Amino Acids: 47
Theoretical Mass: 6034.77 Da
Mass Difference: 0.0609 Da
B Ions: 22 Y Ions: 8

Ion	Observed Mass (Da)	Theoretical Mass (Da)	Mass Error (Da)	Mass Error (PPM)	Delta M
Y46	5645.5626	5645.6278	-0.0652	-11.54	--
	5645.5493	5645.6278	-0.0785	-13.90	--
Y26	3073.4180	3073.3704	0.0476	15.50	--
Y25	2944.3683	2944.3278	0.0405	13.77	--
Y22	2567.1592	2567.1505	0.0087	3.40	--
Y5	664.3210	664.3240	-0.0030	-4.49	--
Y4	517.2528	517.2556	-0.0028	-5.41	--
Y3	331.1738	331.1763	-0.0025	-7.55	--
Y2	260.1367	260.1391	-0.0024	-9.34	--
B45	5774.6272	5774.6300	-0.0028	-0.48	--
B44	5703.5783	5703.5928	-0.0145	-2.54	--
B43	5517.5047	5517.5135	-0.0088	-1.60	--
B42	5370.4380	5370.4451	-0.0071	-1.33	--
B41	5241.4038	5241.4025	0.0012	0.23	--
B40	5112.3781	5112.3599	0.0182	3.55	--
B39	4983.3106	4983.3174	-0.0067	-1.35	--
B38	4868.2878	4868.2904	-0.0026	-0.54	--
B36	4666.2338	4666.2128	0.0210	4.50	--
B34	4424.0744	4424.0749	-0.0005	-0.11	--
B30	3995.8300	3995.8366	-0.0066	-1.66	--
B28	3811.6971	3811.7154	-0.0183	-4.81	--
B27	3696.6807	3696.6885	-0.0078	-2.11	--
B24	3320.5446	3320.5502	-0.0056	-1.68	--
B23	3189.5053	3189.5097	-0.0044	-1.39	--
B22	3090.4345	3090.4413	-0.0069	-2.22	--
B21	2961.3920	2961.3987	-0.0068	-2.28	--
B19	2700.2838	2700.2874	-0.0036	-1.32	--
B18	2571.2390	2571.2448	-0.0058	-2.25	--
B17	2442.2009	2442.2022	-0.0013	-0.52	--
B16	2314.1012	2314.1072	-0.0060	-2.60	--
B7	1190.6246	1190.6274	-0.0028	-2.39	--

Crude PScore: 5.40752e-29
McLuckey Score: 23.5294

Graphical Fragment Mapper

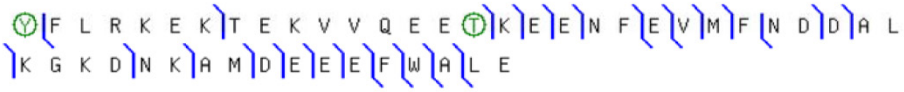

B. AMA1_1

Peptide Summary

Sequence: RASHTTPVLMEKPYY, S3-Phospho (79.96633 Da)
Charge: +3, Monoisotopic m/z: 624.96259 Da (+0.58 mmu/+0.93 ppm), MH+: 1872.87320 Da, RT: 10.88 min,
Identified with: SEQUEST (v1.20); XCorr:2.64, Probability:0.00, Ions matched by search engine: 13/82
Fragment match tolerance used for search: 20 mmu

Fragment Matches

Value Type: Theo. Mass [Da]

Ion Series | Phosphorylation Losses | Neutral Losses | Multiple Neutral Losses | Precursor Ions

#1	b⁺	b²⁺	b³⁺	Seq.	y⁺	y²⁺	y³⁺	#2
1	157.10840	79.05784	53.04098	R				15
2	228.14552	114.57640	76.72002	A	1716.77033	858.88880	572.92830	14
3	395.14388	198.07558	132.38614	S-Phospho	1645.73321	823.37024	549.24926	13
4	532.20279	266.60503	178.07245	H	1478.73485	739.87106	493.58313	12
5	633.25047	317.12887	211.75501	T	1341.67594	671.34161	447.89683	11
6	734.29815	367.65271	245.43757	T	1240.62826	620.81777	414.21427	10
7	831.35092	416.17910	277.78849	P	1139.58058	570.29393	380.53171	9
8	930.41934	465.71331	310.81130	V	1042.52781	521.76754	348.18079	8
9	1043.50341	522.25534	348.50599	L	943.45939	472.23333	315.15798	7
10	1174.54391	587.77559	392.18615	M	830.37532	415.69130	277.46329	6
11	1303.58651	652.29689	435.20035	E	699.33482	350.17105	233.78312	5
12	1431.68148	716.34438	477.89868	K	570.29222	285.64975	190.76892	4
13	1528.73425	764.87076	510.24960	P	442.19725	221.60226	148.07060	3
14	1691.79757	846.40242	564.60404	Y	345.14448	173.07588	115.71968	2
15				Y	182.08116	91.54422	61.36524	1

Fragment Spectrum

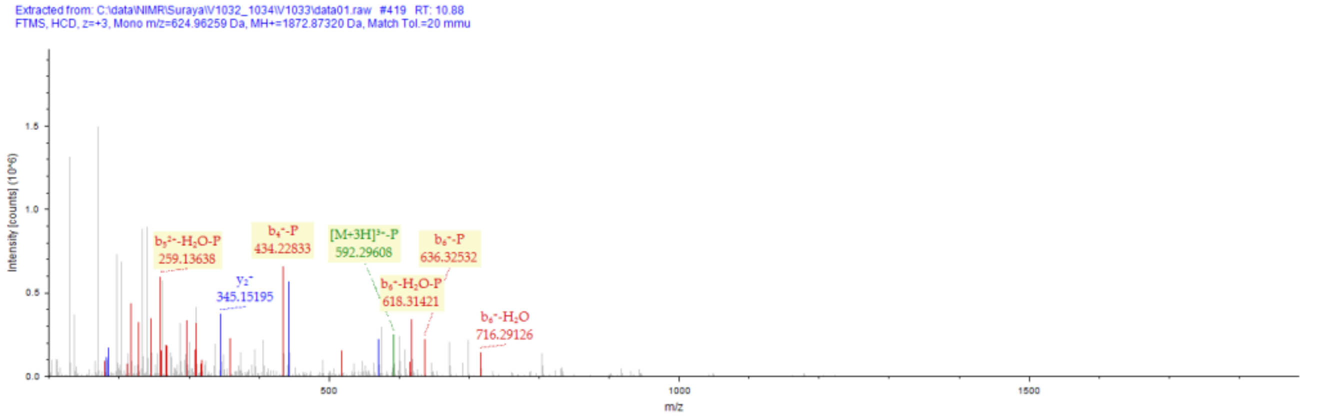

Sequence: RASHTTPVLMEKPYY
S3-Phospho (79.96633 Da)
Charge: +3, Monoisotopic m/z: 624.96259 Da (+0.58 mmu/+0.93 ppm), MH+: 1872.87320 Da, RT: 10.88 min,

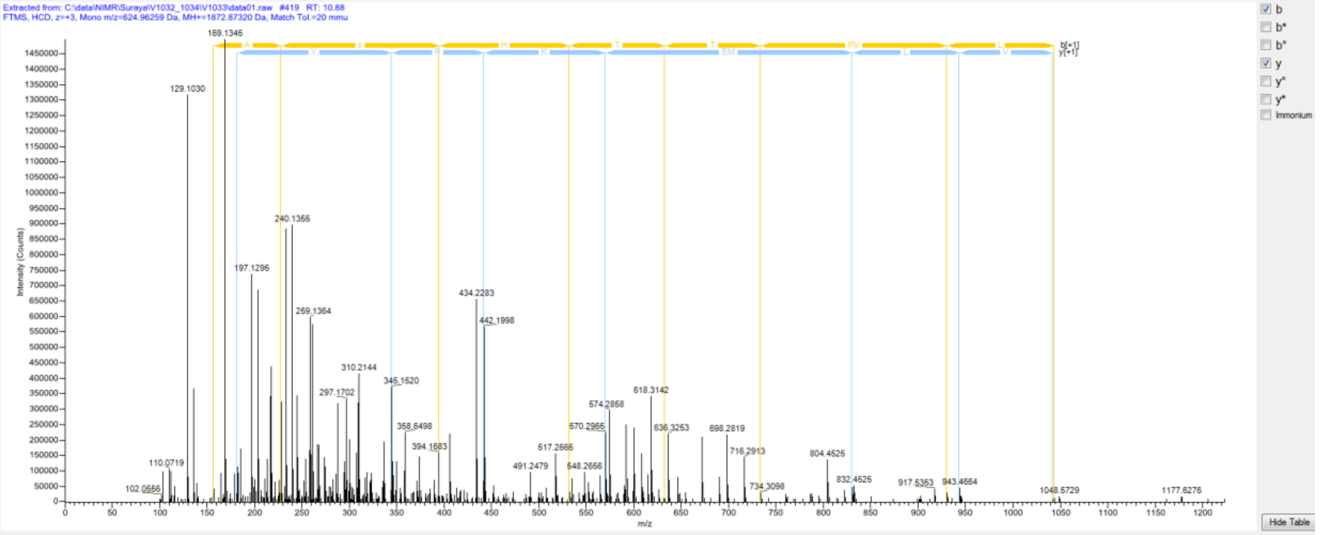

	(NH3+) -	A> <s	s> <H	H> <T	T> <T	T> <P	P> <V	V> <L	L> <M	M> <E	E> <K	K> <P	P> <Y	Y> <Y	Y - (COOH)
b (+1)	157.10840	228.14552	395.14388	532.20279	633.25047	734.29815	831.35092	930.41934	1043.50341	1174.54391	1303.58651	1431.68148	1528.73425	1691.79757	1854.86089
b*(+1)	140.08185	211.11897	378.11733	515.17624	616.22392	717.27160	814.32437	913.39279	1026.47686	1157.51736	1286.55996	1414.65493	1511.70770	1674.77102	1837.83434
b°(+1)	139.09783	210.13495	377.13331	514.19222	615.23990	716.28758	813.34035	912.40877	1025.49284	1156.53334	1285.57594	1413.67091	1510.72368	1673.78700	1836.85032
y (+1)	1716.77033	1645.73321	1478.73485	1341.67594	1240.62826	1139.58058	1042.52781	943.45939	830.37532	699.33482	570.29222	442.19725	345.14448	182.08116	1872.87145
y*(+1)	1699.74378	1628.70666	1461.70830	1324.64939	1223.60171	1122.55403	1025.50126	926.43284	813.34877	682.30827	553.26567	425.17070	328.11793	165.05461	1855.84490
y°(+1)	1698.75977	1627.72265	1460.72429	1323.66538	1222.61770	1121.57002	1024.51725	925.44883	812.36476	681.32426	552.28166	424.18669	327.13392	164.07060	1854.86089

Value Type: Theo. Mass [Da]

R A s H T T P V L M E K P Y Y

B. AMA1_2

Peptide Summary

Sequence: RASHTTPVLMEKPYY, S3-Phospho (79.96633 Da)
Charge: +3, Monoisotopic m/z: 624.96167 Da (-0.33 mmu/-0.53 ppm), MH+: 1872.87046 Da, RT: 11.73 min,
Identified with: SEQUEST (v1.20); XCorr:4.58, Probability:0.00, Ions matched by search engine: 36/82
Fragment match tolerance used for search: 0.8 Da

Fragment Matches

Value Type: Theo. Mass [Da]

Ion Series | Phosphorylation Losses | Neutral Losses | Multiple Neutral Losses | Precursor Ions

#1	b+	b2+	b3+	Seq.	y+	y2+	y3+	#2
1	157.10840	79.05784	53.04098	R				15
2	228.14552	114.57640	76.72002	A	1716.77033	858.88880	572.92830	14
3	395.14388	198.07558	132.38614	S-Phospho	1645.73321	823.37024	549.24926	13
4	532.20279	266.60503	178.07245	H	1478.73485	739.87106	493.58313	12
5	633.25047	317.12887	211.75501	T	1341.67594	671.34161	447.89683	11
6	734.29815	367.65271	245.43757	T	1240.62826	620.81777	414.21427	10
7	831.35092	416.17910	277.78849	P	1139.58058	570.29393	380.53171	9
8	930.41934	465.71331	310.81130	V	1042.52781	521.76754	348.18079	8
9	1043.50341	522.25534	348.50599	L	943.45939	472.23333	315.15798	7
10	1174.54391	587.77559	392.18615	M	830.37532	415.69130	277.46329	6
11	1303.58651	652.29689	435.20035	E	699.33482	350.17105	233.78312	5
12	1431.68148	716.34438	477.89868	K	570.29222	285.64975	190.76892	4
13	1528.73425	764.87076	510.24960	P	442.19725	221.60226	148.07060	3
14	1691.79757	846.40242	564.60404	Y	345.14448	173.07588	115.71968	2
15				Y	182.08116	91.54422	61.36524	1

Fragment Spectrum

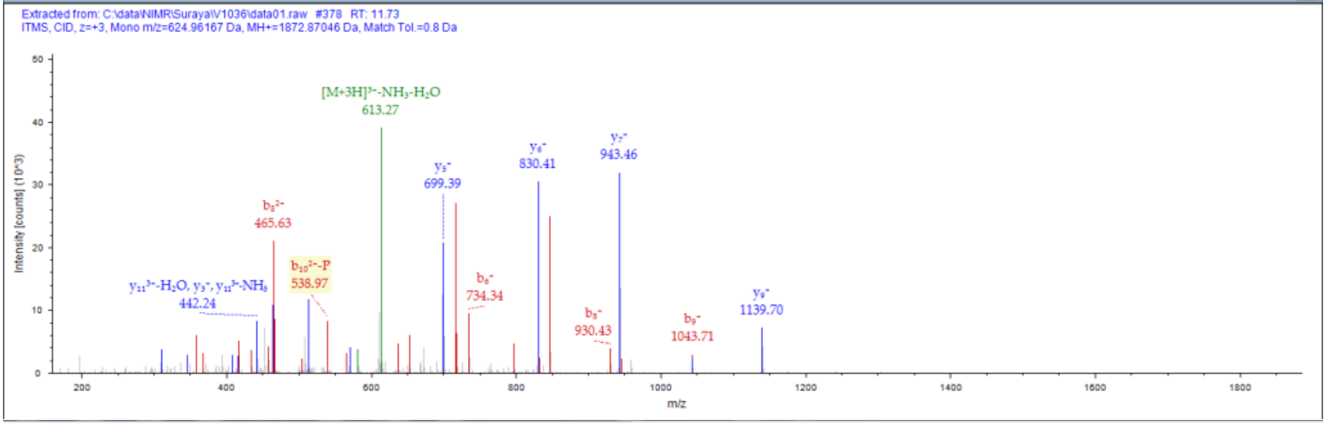

Sequence: RASHTTPVLMEKPYY
S3-Phospho (79.96633 Da)
Charge: +3, Monoisotopic m/z: 624.96167 Da (-0.33 mmu/-0.53 ppm), MH+: 1872.87046 Da, RT: 11.73 min,

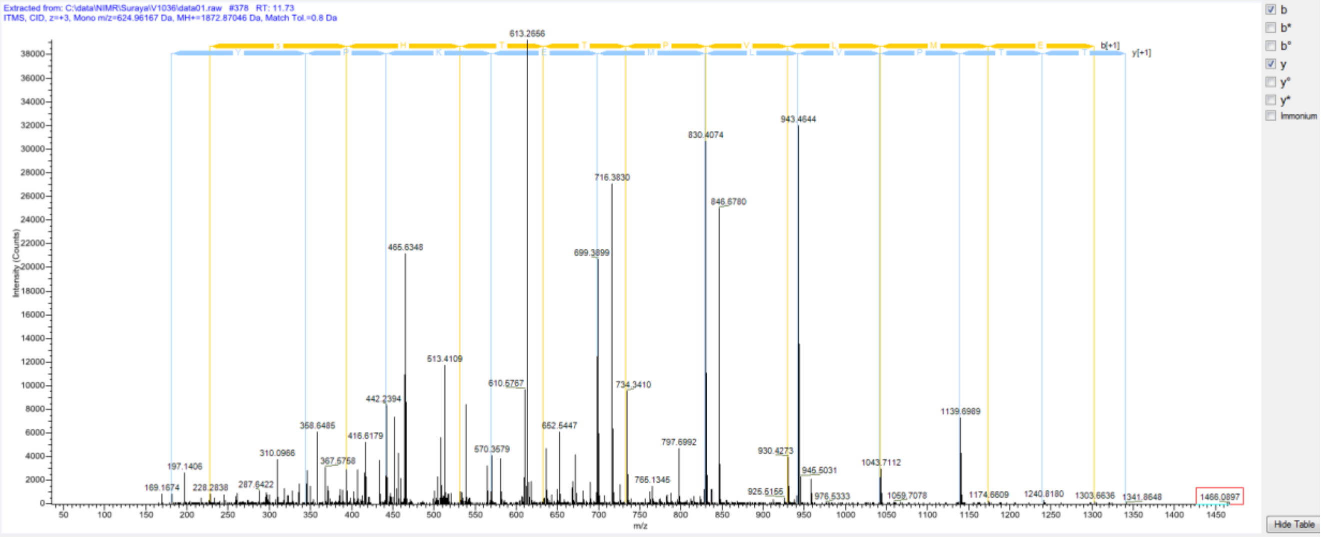

	(NH3+) -	A> <s	s> <H	H> <T	T> <T	T> <P	P> <V	V> <L	L> <M	M> <E	E> <K	K> <P	P> <Y	Y> <Y	Y - (COOH)
b (+1)	157.10840	228.14552	395.14388	532.20279	633.25047	734.29815	831.35092	930.41934	1043.50341	1174.54391	1303.58651	1431.68148	1528.73425	1691.79757	1854.86089
b*(+1)	140.08185	211.11897	378.11733	515.17624	616.22392	717.27160	814.32437	913.39279	1026.47686	1157.51736	1286.55996	1414.65493	1511.70770	1674.77102	1837.83434
b°(+1)	139.09783	210.13495	377.13331	514.19222	615.23990	716.28758	813.34035	912.40877	1025.49284	1156.53334	1285.57594	1413.67091	1510.72368	1673.78700	1836.85032
y (+1)	1716.77033	1645.73321	1478.73485	1341.67594	1240.62826	1139.58058	1042.52781	943.45939	830.37532	699.33482	570.29222	442.19725	345.14448	182.08116	1872.87145
y*(+1)	1699.74378	1628.70666	1461.70830	1324.64939	1223.60171	1122.55403	1025.50126	926.43284	813.34877	682.30827	553.26567	425.17070	328.11793	165.05461	1855.84490
y°(+1)	1698.75977	1627.72265	1460.72429	1323.66538	1222.61770	1121.57002	1024.51725	925.44883	812.36476	681.32426	552.28166	424.18669	327.13392	164.07060	1854.86089

Value Type: Theo. Mass [Da]

R A s H T T P V L M E K P Y Y

B. AMA1_3

Peptide Summary

Sequence: RASHTTPVLMEKPYY, S3-Phospho (79.96633 Da)
Charge: +2, Monoisotopic m/z: 936.93909 Da (-0.28 mmu/-0.3 ppm), MH+: 1872.87090 Da, RT: 10.06 min,
Identified with: SEQUEST (v1.20); XCorr:3.69, Probability:0.00, Ions matched by search engine: 15/41
Fragment match tolerance used for search: 20 mmu

Fragment Matches

Value Type: Theo. Mass [Da]

Ion Series | Phosphorylation Losses | Neutral Losses | Multiple Neutral Losses | Precursor Ions

#1	b+	b2+	Seq.	y+	y2+	#2
1	157.10840	79.05784	R			15
2	228.14552	114.57640	A	1716.77033	858.88880	14
3	395.14388	198.07558	S-Phospho	1645.73321	823.37024	13
4	532.20279	266.60503	H	1478.73485	739.87106	12
5	633.25047	317.12887	T	1341.67594	671.34161	11
6	734.29815	367.65271	T	1240.62826	620.81777	10
7	831.35092	416.17910	P	1139.58058	570.29393	9
8	930.41934	465.71331	V	1042.52781	521.76754	8
9	1043.50341	522.25534	L	943.45939	472.23333	7
10	1174.54391	587.77559	M	830.37532	415.69130	6
11	1303.58651	652.29689	E	699.33482	350.17105	5
12	1431.68148	716.34438	K	570.29222	285.64975	4
13	1528.73425	764.87076	P	442.19725	221.60226	3
14	1691.79757	846.40242	Y	345.14448	173.07588	2
15			Y	182.08116	91.54422	1

Fragment Spectrum

Extracted from: C:\data\NIMR\Suraya\V1037_38\data01.raw #418 RT: 10.06
FTMS, CID, z=+2, Mono m/z=936.93909 Da, MH+=1872.87090 Da, Match Tol.=20 mmu

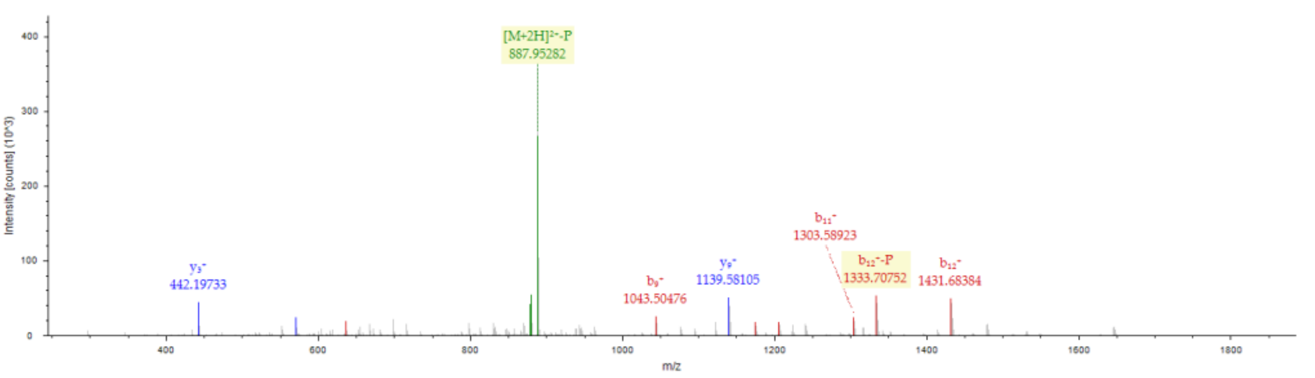

Sequence: RASHTTPVLMEKPYY
S3-Phospho (79.96633 Da)
Charge: +2, Monoisotopic m/z: 936.93909 Da (-0.28 mmu/-0.3 ppm), MH+: 1872.87090 Da, RT: 10.06 min,

Extracted from: C:\data\NIMR\Suraya\V1037_38\data01.raw #418 RT: 10.06
FTMS, CID, z=+2, Mono m/z=936.93909 Da, MH+=1872.87090 Da, Match Tol.=20 mmu

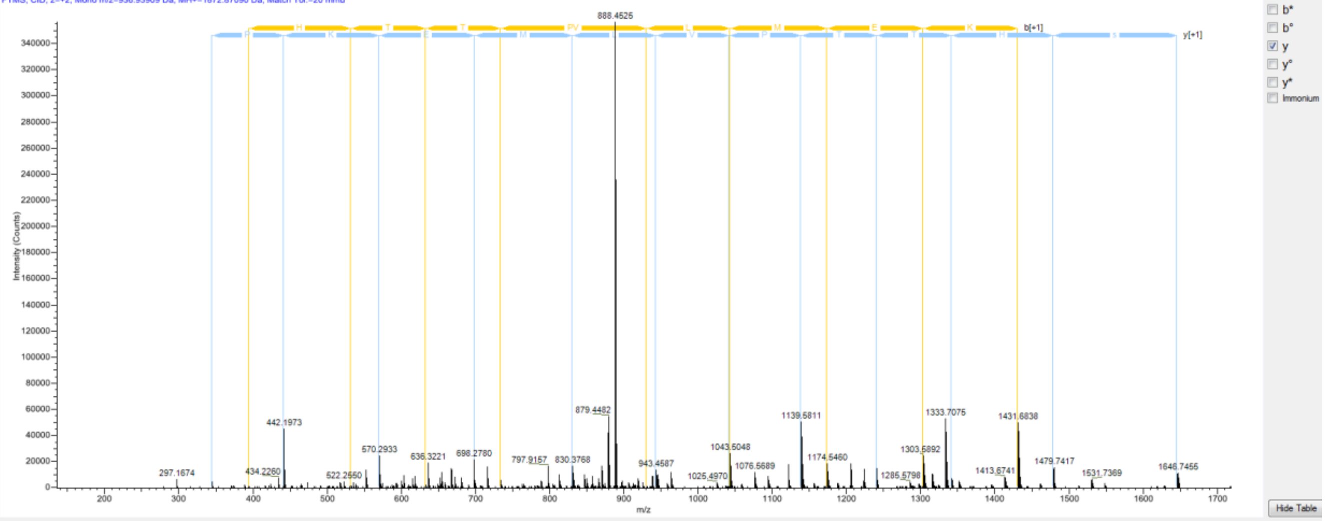

	(NH3+) -	A> <s	s> <H	H> <T	T> <T	T> <P	P> <V	V> <L	L> <M	M> <E	E> <K	K> <P	P> <Y	Y> <Y	Y - (COOH)
b (+1)	157.10840	228.14552	395.14388	532.20279	633.25047	734.29815	831.35092	930.41934	1043.50341	1174.54391	1303.58651	1431.68148	1528.73425	1691.79757	1854.86089
b*(+1)	140.08185	211.11897	378.11733	515.17624	616.22392	717.27160	814.32437	913.39279	1026.47686	1157.51736	1286.55996	1414.65493	1511.70770	1674.77102	1837.83434
b°(+1)	139.09783	210.13495	377.13331	514.19222	615.23990	716.28758	813.34035	912.40877	1025.49284	1156.53334	1285.57594	1413.67091	1510.72368	1673.78700	1836.85032
y (+1)	1716.77033	1645.73321	1478.73485	1341.67594	1240.62826	1139.58058	1042.52781	943.45939	830.37532	699.33482	570.29222	442.19725	345.14448	182.08116	1872.87145
y*(+1)	1699.74378	1628.70666	1461.70830	1324.64939	1223.60171	1122.55403	1025.50126	926.43284	813.34877	682.30827	553.26567	425.17070	328.11793	165.05461	1855.84490
y°(+1)	1698.75977	1627.72265	1460.72429	1323.66538	1222.61770	1121.57002	1024.51725	925.44883	812.36476	681.32426	552.28166	424.18669	327.13392	164.07060	1854.86089

Value Type: Theo. Mass [Da]

R A s H T T P V L M E K P Y Y

B. AMA1_4

Peptide Summary

Sequence: RASHTTPVLMEKPYY, S3-Phospho (79.96633 Da)
Charge: +2, Monoisotopic m/z: 936.93884 Da (-0.52 mmu/-0.56 ppm), MH+: 1872.87041 Da, RT: 12.05 min,
Identified with: SEQUEST (v1.20); XCorr:2.22, Probability:0.00, Ions matched by search engine: 18/41
Fragment match tolerance used for search: 20 mmu

Fragment Matches

Value Type: Theo. Mass [Da]

Ion Series | Phosphorylation Losses | Neutral Losses | Multiple Neutral Losses | Precursor Ions

#1	b+	b2+	Seq.	y+	y2+	#2
1	157.10840	79.05784	R			15
2	228.14552	114.57640	A	1716.77033	858.88880	14
3	395.14388	198.07558	S-Phospho	1645.73321	823.37024	13
4	532.20279	266.60503	H	1478.73485	739.87106	12
5	633.25047	317.12887	T	1341.67594	671.34161	11
6	734.29815	367.65271	T	1240.62826	620.81777	10
7	831.35092	416.17910	P	1139.58058	570.29393	9
8	930.41934	465.71331	V	1042.52781	521.76754	8
9	1043.50341	522.25534	L	943.45939	472.23333	7
10	1174.54391	587.77559	M	830.37532	415.69130	6
11	1303.58651	652.29689	E	699.33482	350.17105	5
12	1431.68148	716.34438	K	570.29222	285.64975	4
13	1528.73425	764.87076	P	442.19725	221.60226	3
14	1691.79757	846.40242	Y	345.14448	173.07588	2
15			Y	182.08116	91.54422	1

Fragment Spectrum

Extracted from: C:\data\NIMR\Suraya\V1037_38\data02.raw #512 RT: 12.05
FTMS, CID, z=+2, Mono m/z=936.93884 Da, MH+=1872.87041 Da, Match Tol.=20 mmu

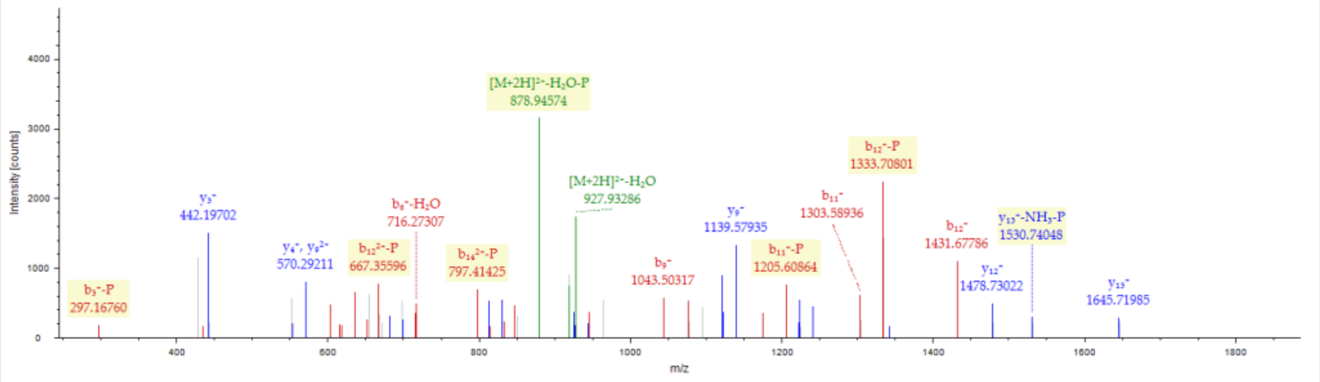

Sequence: RASHTTPVLMEKPYY
S3-Phospho (79.96633 Da)
Charge: +2, Monoisotopic m/z: 936.93884 Da (-0.52 mmu/-0.56 ppm), MH+: 1872.87041 Da, RT: 12.05 min,

Extracted from: C:\data\NIMR\Suraya\V1037_38\data02.raw #512 RT: 12.05
FTMS, CID, z=+2, Mono m/z=936.93884 Da, MH+=1872.87041 Da, Match Tol.=20 mmu

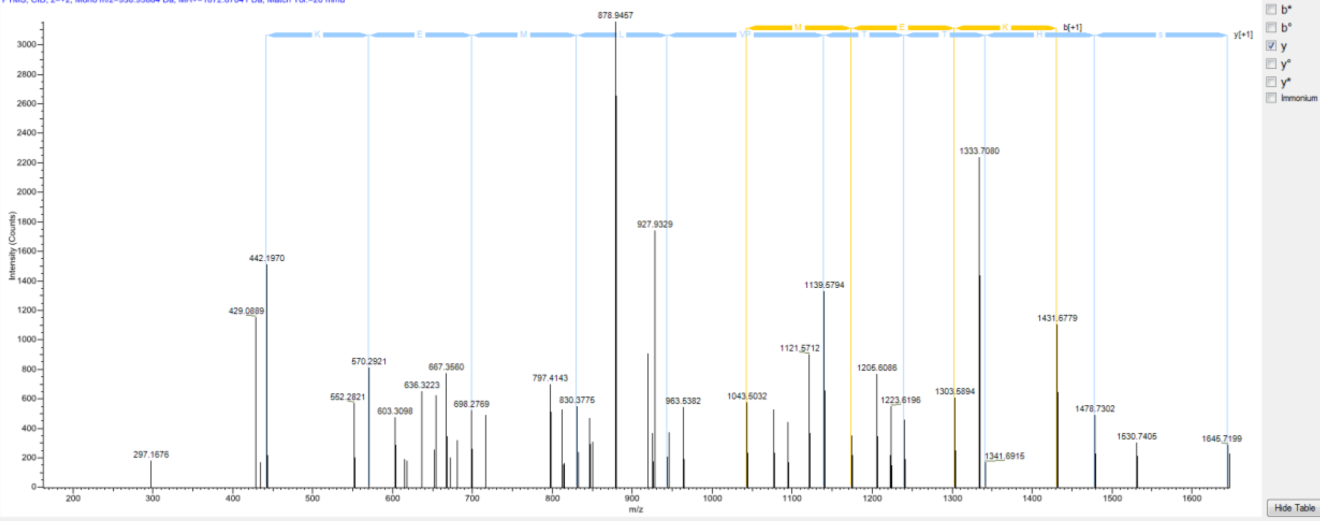

	(NH3+) -	A> <s	s> <H	H> <T	T> <T	T> <P	P> <V	V> <L	L> <M	M> <E	E> <K	K> <P	P> <Y	Y> <Y	Y - (COOH)
b (+1)	157.10840	228.14552	395.14388	532.20279	633.25047	734.29815	831.35092	930.41934	1043.50341	1174.54391	1303.58651	1431.68148	1528.73425	1691.79757	1854.86089
b*(+1)	140.08185	211.11897	378.11733	515.17624	616.22392	717.27160	814.32437	913.39279	1026.47686	1157.51736	1286.55996	1414.65493	1511.70770	1674.77102	1837.83434
b°(+1)	139.09783	210.13495	377.13331	514.19222	615.23990	716.28758	813.34035	912.40877	1025.49284	1156.53334	1285.57594	1413.67091	1510.72368	1673.78700	1836.85032
y (+1)	1716.77033	1645.73321	1478.73485	1341.67594	1240.62826	1139.58058	1042.52781	943.45939	830.37532	699.33482	570.29222	442.19725	345.14448	182.08116	1872.87145
y*(+1)	1699.74378	1628.70666	1461.70830	1324.64939	1223.60171	1122.55403	1025.50126	926.43284	813.34877	682.30827	553.26567	425.17070	328.11793	165.05461	1855.84490
y°(+1)	1698.75977	1627.72265	1460.72429	1323.66538	1222.61770	1121.57002	1024.51725	925.44883	812.36476	681.32426	552.28166	424.18669	327.13392	164.07060	1854.86089

Value Type: Theo. Mass [Da]

R A s H T T P V L M E K P Y Y

B. AMA1_5

Peptide Summary

Sequence: RASHTTPVLMEKPYY, S3-Phospho (79.96633 Da), M10-Oxidation (15.99492 Da)
Charge: +2, Monoisotopic m/z: 944.93518 Da (-1.64 mmu/-1.74 ppm), MH+: 1888.86308 Da, RT: 8.84 min,
Identified with: SEQUEST (v1.20); XCorr:3.46, Probability:0.00, Ions matched by search engine: 19/55
Fragment match tolerance used for search: 20 mmu

Fragment Matches

Value Type: Theo. Mass [Da]

Ion Series | Phosphorylation Losses | Neutral Losses | Multiple Neutral Losses | Precursor Ions

#1	a^{+}	a^{2+}	b^{+}	b^{2+}	Seq.	y^{+}	y^{2+}	#2
1	129.11348	65.06038	157.10840	79.05784	R			15
2	200.15060	100.57894	228.14552	114.57640	A	1732.76525	866.88626	14
3	367.14896	184.07812	395.14388	198.07558	S-Phospho	1661.72813	831.36770	13
4	504.20787	252.60757	532.20279	266.60503	H	1494.72977	747.86852	12
5	605.25555	303.13141	633.25047	317.12887	T	1357.67086	679.33907	11
6	706.30323	353.65525	734.29815	367.65271	T	1256.62318	628.81523	10
7	803.35600	402.18164	831.35092	416.17910	P	1155.57550	578.29139	9
8	902.42442	451.71585	930.41934	465.71331	V	1058.52273	529.76500	8
9	1015.50849	508.25788	1043.50341	522.25534	L	959.45431	480.23079	7
10	1162.54391	581.77559	1190.53882	595.77305	M-Oxidation	846.37024	423.68876	6
11	1291.58651	646.29689	1319.58142	660.29435	E	699.33482	350.17105	5
12	1419.68148	710.34438	1447.67639	724.34183	K	570.29222	285.64975	4
13	1516.73425	758.87076	1544.72916	772.86822	P	442.19725	221.60226	3
14	1679.79757	840.40242	1707.79248	854.39988	Y	345.14448	173.07588	2
15					Y	182.08116	91.54422	1

Fragment Spectrum

Extracted from: C:\data\NIMR\Suraya\V1035\data01.raw #546 RT: 8.84
FTMS, HCD, z=+2, Mono m/z=944.93518 Da, MH+=1888.86308 Da, Match Tol.=20 mmu

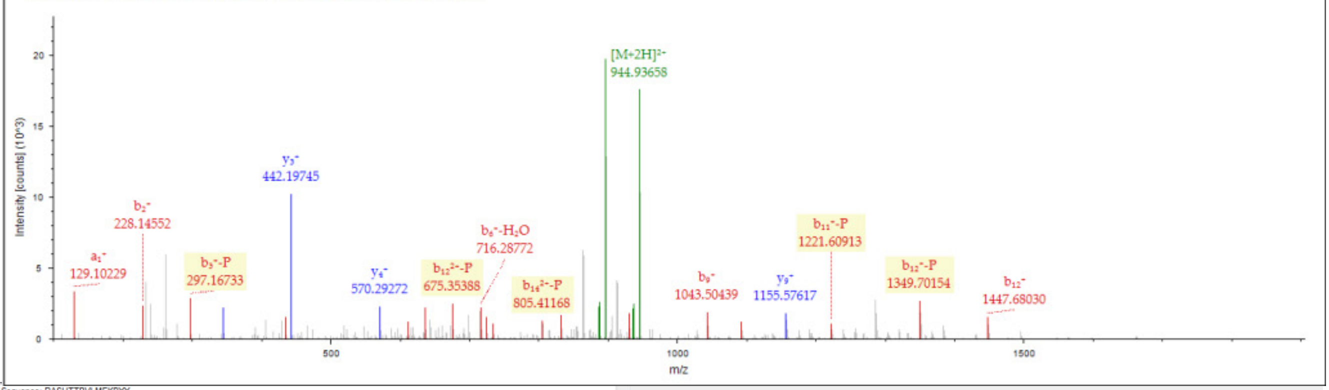

Sequence: RASHTTPVLMEKPYY
S3-Phospho (79.96633 Da), M10-Oxidation (15.99492 Da)
Charge: +2, Monoisotopic m/z: 944.93518 Da (-1.64 mmu/-1.74 ppm), MH+: 1888.86308 Da, RT: 8.84 min,

Extracted from: C:\data\NIMR\Suraya\V1035\data01.raw #546 RT: 8.84
FTMS, HCD, z=+2, Mono m/z=944.93518 Da, MH+=1888.86308 Da, Match Tol.=20 mmu

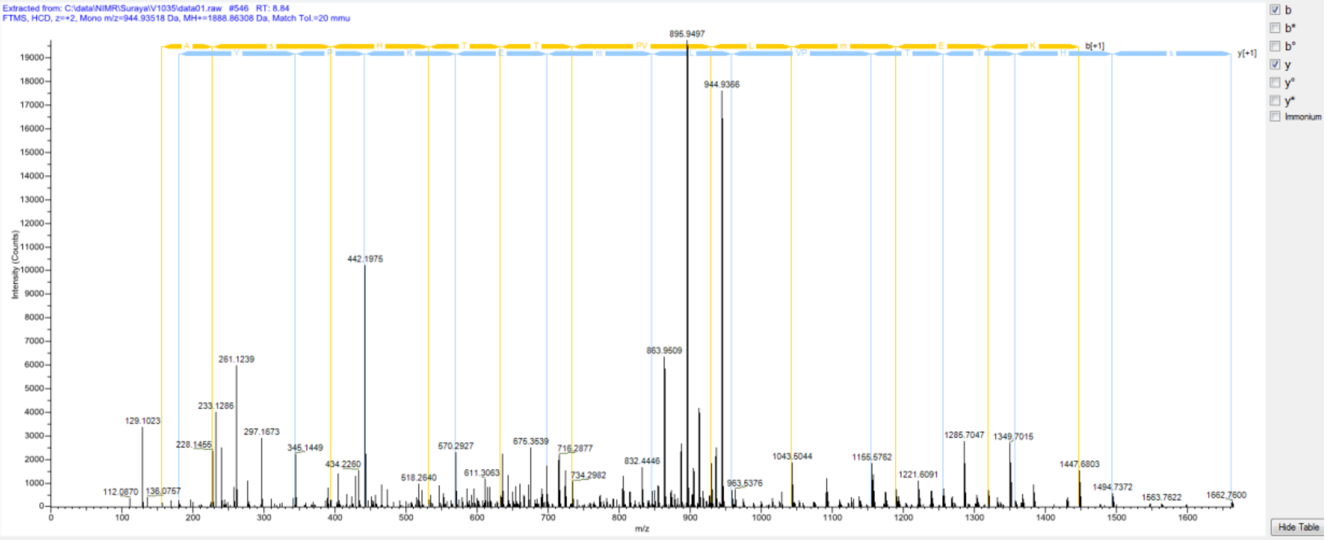

Hide Table

	(NH3+) -	A> <s	s> <H	H> <T	T> <T	T> <P	P> <V	V> <L	L> <m	m> <E	E> <K	K> <P	P> <Y	Y> <Y	Y - (COOH)
b (+1)	157.10840	228.14552	395.14388	532.20279	633.25047	734.29815	831.35092	930.41934	1043.50341	1190.53882	1319.58142	1447.67639	1544.72916	1707.79248	1870.85580
b*(+1)	140.08185	211.11897	378.11733	515.17624	616.22392	717.27160	814.32437	913.39279	1026.47686	1173.51227	1302.55487	1430.64984	1527.70261	1690.76593	1853.82925
b°(+1)	139.09783	210.13495	377.13331	514.19222	615.23990	716.28758	813.34035	912.40877	1025.49284	1172.52826	1301.57086	1429.66583	1526.71860	1689.78192	1852.84524
y (+1)	1732.76525	1661.72813	1494.72977	1357.67086	1256.62318	1155.57550	1058.52273	959.45431	846.37024	699.33482	570.29222	442.19725	345.14448	182.08116	1888.86637
y*(+1)	1715.73870	1644.70158	1477.70322	1340.64431	1239.59663	1138.54895	1041.49618	942.42776	829.34369	682.30827	553.26567	425.17070	328.11793	165.05461	1871.83982
y°(+1)	1714.75468	1643.71756	1476.71920	1339.66029	1238.61261	1137.56493	1040.51216	941.44374	828.35967	681.32426	552.28166	424.18669	327.13392	164.07060	1870.85580

Value Type: Theo. Mass [Da]

R A *s* H T T P V L *m* E K P Y Y

C. RH1

[226.0776]DNNKMDDK[79.96633]STQKYGRNQEEVMEIFFDNDYI
Number of Amino Acids: 30
Theoretical Mass: 3991.68 Da
Mass Difference: -0.67593 Da
B Ions: 20 Y Ions: 13

Ion	Observed Mass (Da)	Theoretical Mass (Da)	Mass Error (Da)	Mass Error (PPM)	Delta M
Y29	3650.5547	3650.5714	-0.0167	-4.57	--
Y26	3294.3863	3294.3906	-0.0043	-1.31	--
Y23	2933.3052	2933.2962	0.0090	3.07	--
Y20	2537.1494	2537.1552	-0.0058	-2.29	--
Y19	2409.0935	2409.0966	-0.0031	-1.29	--
Y18	2280.9943	2281.0017	-0.0073	-3.21	--
Y17	2117.9357	2117.9383	-0.0027	-1.26	--
Y13	1662.7472	1662.7143	0.0330	19.83	--
Y10	1305.5570	1305.5607	-0.0037	-2.81	--
Y8	1045.4729	1045.4776	-0.0047	-4.45	--
Y7	932.3895	932.3935	-0.0041	-4.36	--
Y6	785.3211	785.3251	-0.0040	-5.09	--
Y2	294.1574	294.1599	-0.0025	-8.58	--
B28	3697.5063	3697.5160	-0.0097	-2.63	--
B27	3582.5180	3582.4891	0.0289	8.07	--
B26	3468.4673	3468.4462	0.0212	6.11	--
B25	3353.4500	3353.4192	0.0308	9.18	--
B24	3206.3456	3206.3508	-0.0052	-1.63	--
B23	3059.2746	3059.2824	-0.0078	-2.57	--
B22	2946.1731	2946.1983	-0.0253	-8.58	--
B21	2817.1543	2817.1558	-0.0014	-0.51	--
B20	2686.1094	2686.1153	-0.0058	-2.18	--
B18	2457.9983	2458.0043	-0.0060	-2.42	--
B15	2086.8442	2086.8602	-0.0159	-7.64	--
B13	1873.7349	1873.7376	-0.0027	-1.45	--
B12	1710.6694	1710.6743	-0.0048	-2.82	--
B10	1454.5205	1454.5207	-0.0002	-0.15	--
B8	1186.4719	1186.4747	-0.0028	-2.38	--
B7	1058.3769	1058.3797	-0.0029	-2.72	--
B6	943.3503	943.3528	-0.0024	-2.59	--
B5	828.3234	828.3258	-0.0024	-2.95	--
B3	569.1892	569.1904	-0.0012	-2.05	--
B1	341.1039	341.1045	-0.0007	-1.98	--

Crude PScore: 1.60658e-39
McLuckey Score: 37.9147

Graphical Fragment Mapper

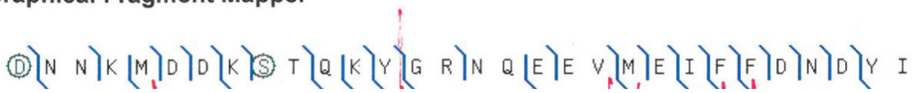

D. RH4_1

Peptide Summary

Sequence: KEFSEADNAHSEEK, S4-Phospho (79.96633 Da)
Charge: +3, Monoisotopic m/z: 567.56464 Da (-0.22 mmu/-0.39 ppm), MH+: 1700.67936 Da, RT: 10.94 min,
Identified with: Mascot (v1.27); IonScore:53, Exp Value:8.9E-006, Ions matched by search engine: 15/156
Fragment match tolerance used for search: 20 mmu

Fragment Matches

Value Type: Theo. Mass [Da]

Ion Series | Phosphorylation Losses | Neutral Losses | Multiple Neutral Losses | Precursor Ions

#1	a⁺	a²⁺	a³⁺	b⁺	b²⁺	b³⁺	Seq.	y⁺	y²⁺	y³⁺	#2
1	101.10733	51.05730	34.37396	129.10225	65.05476	43.70560	K				14
2	230.14993	115.57860	77.38816	258.14485	129.57606	86.71980	E	1572.58505	786.79616	524.86654	13
3	377.21835	189.11281	126.41097	405.21327	203.11027	135.74261	F	1443.54245	722.27486	481.85234	12
4	544.21671	272.61199	182.07709	572.21163	286.60945	191.40873	S-Phospho	1296.47403	648.74065	432.82953	11
5	673.25931	337.13329	225.09129	701.25423	351.13075	234.42293	E	1129.47567	565.24147	377.16341	10
6	744.29643	372.65185	248.77033	772.29135	386.64931	258.10197	A	1000.43307	500.72017	334.14921	9
7	859.32338	430.16533	287.11265	887.31830	444.16279	296.44428	D	929.39595	465.20161	310.47017	8
8	973.36631	487.18679	325.12696	1001.36123	501.18425	334.45859	N	814.36900	407.68814	272.12785	7
9	1044.40343	522.70535	348.80600	1072.39835	536.70281	358.13763	A	700.32607	350.66667	234.11354	6
10	1181.46234	591.23481	394.49230	1209.45726	605.23227	403.82394	H	629.28895	315.14811	210.43450	5
11	1268.49437	634.75082	423.50298	1296.48929	648.74828	432.83461	S	492.23004	246.61866	164.74820	4
12	1397.53697	699.27212	466.51718	1425.53189	713.26958	475.84881	E	405.19801	203.10264	135.73752	3
13	1526.57957	763.79342	509.53138	1554.57449	777.79088	518.86301	E	276.15541	138.58134	92.72332	2
14							K	147.11281	74.06004	49.70912	1

Fragment Spectrum

Extracted from: C:\Users\SAH\Desktop\Data\Suraya\V2085_EBA181_RH4_tryp_chymo\V2087_RH4_P_tryp_200fmol_43C_70m09233648H4C4.raw #197 RT: 10.94
FTMS, HCD, z=+3, Mono m/z=567.56464 Da, MH+=1700.67936 Da, Match Tol.=20 mmu

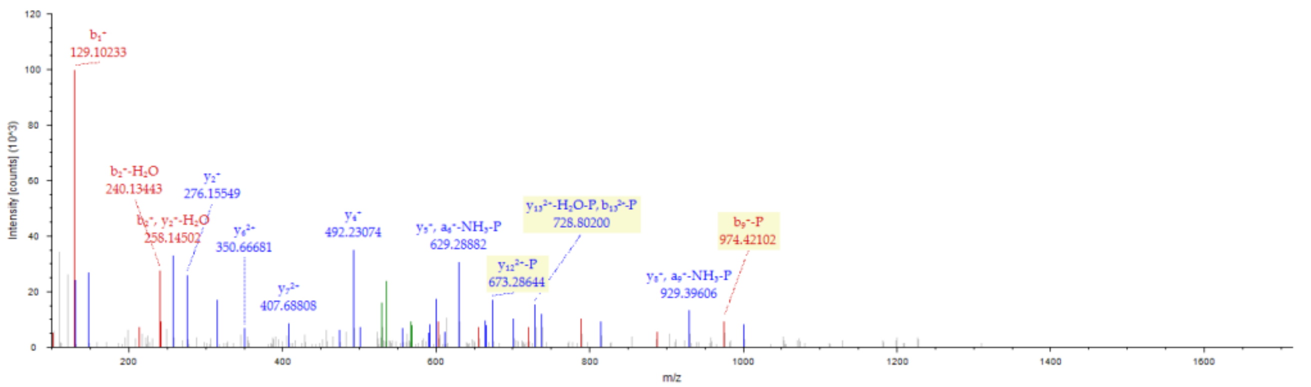

Sequence: KEFSEADNAHSEEK
S4-Phospho (79.96633 Da)
Charge: +3, Monoisotopic m/z: 567.56464 Da (-0.22 mmu/-0.39 ppm), MH+: 1700.67936 Da, RT: 10.94 min,

Extracted from: C:\Users\SAH\Desktop\Data\Suraya\V2085_EBA181_RH4_tryp_chymo\V2087_RH4_P_tryp_200fmol_43C_70m09233648H4C4.raw #197 RT: 10.94
FTMS, HCD, z=+3, Mono m/z=567.56464 Da, MH+=1700.67936 Da, Match Tol.=20 mmu

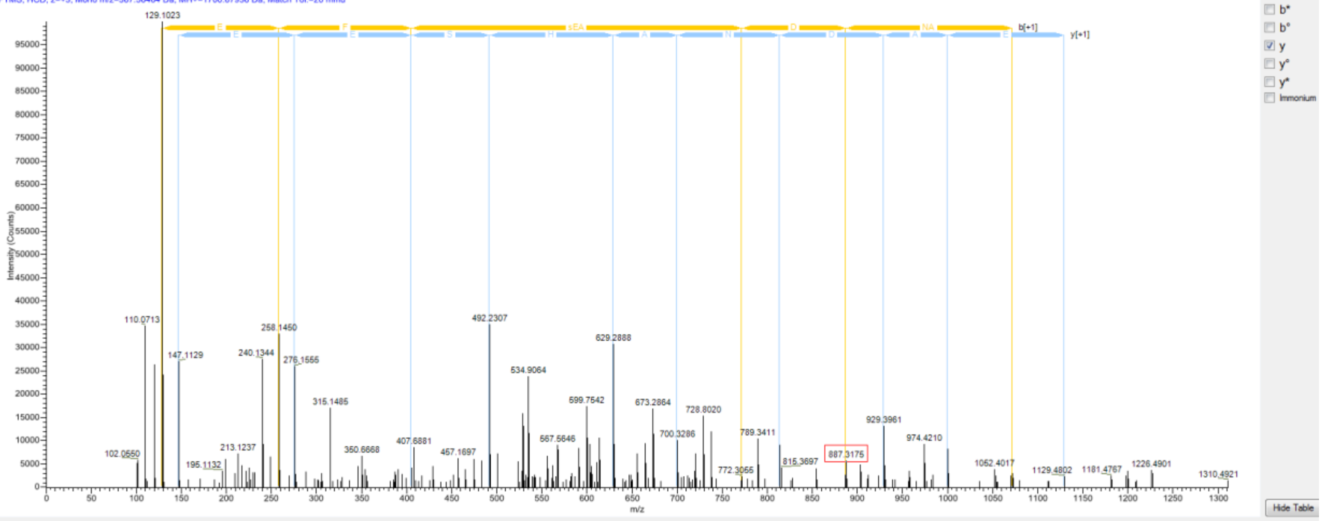

	(NH3+) -	E> <F	F> <s	s> <E	E> <A	A>	D>	N> <A	A> <H	H> <S	S> <E	E> <E	E> <K	K - (COOH)
b (+1)	129.10225	258.14485	405.21327	572.21163	701.25423	772.29135	887.31830	1001.36123	1072.39835	1209.45726	1296.48929	1425.53189	1554.57449	1682.66946
b*(+1)	112.07570	241.11830	388.18672	555.18508	684.22768	755.26480	870.29175	984.33468	1055.37180	1192.43071	1279.46274	1408.50534	1537.54794	1665.64291
b°(+1)	111.09168	240.13428	387.20270	554.20106	683.24366	754.28078	869.30773	983.35066	1054.38778	1191.44669	1278.47872	1407.52132	1536.56392	1664.65889
y (+1)	1572.58505	1443.54245	1296.47403	1129.47567	1000.43307	929.39595	814.36900	700.32607	629.28895	492.23004	405.19801	276.15541	147.11281	1700.68002
y*(+1)	1555.55850	1426.51590	1279.44748	1112.44912	983.40652	912.36940	797.34245	683.29952	612.26240	475.20349	388.17146	259.12886	130.08626	1683.65347
y°(+1)	1554.57449	1425.53189	1278.46347	1111.46511	982.42251	911.38539	796.35844	682.31551	611.27839	474.21948	387.18745	258.14485	129.10225	1682.66946

Value Type: Theo. Mass [Da]

K⌋E⌋F⌋**s**⌈E⌈A⌋D⌋N⌈A⌋H⌈S⌈E⌈E⌈K

D. RH4_2

Peptide Summary

Sequence: EFSEADNAHSE, S3-Phospho (79.96633 Da)
Charge: +2, Monoisotopic m/z: 658.22736 Da (-0.02 mmu/-0.04 ppm), MH+: 1315.44744 Da, RT: 18.46 min,
Identified with: Mascot (v1.27); IonScore:32, Exp Value:5.7E-004, Ions matched by search engine: 5/88
Fragment match tolerance used for search: 20 mmu

Fragment Matches

Value Type: Theo. Mass [Da]

Ion Series | Phosphorylation Losses | Neutral Losses | Multiple Neutral Losses | Precursor Ions

#1	a+	a2+	b+	b2+	Seq.	y+	y2+	#2
1	102.05496	51.53112	130.04988	65.52858	E			11
2	249.12338	125.06533	277.11830	139.06279	F	1186.40488	593.70608	10
3	416.12174	208.56451	444.11666	222.56197	S-Phospho	1039.33646	520.17187	9
4	545.16434	273.08581	573.15926	287.08327	E	872.33810	436.67269	8
5	616.20146	308.60437	644.19638	322.60183	A	743.29550	372.15139	7
6	731.22841	366.11784	759.22333	380.11530	D	672.25838	336.63283	6
7	845.27134	423.13931	873.26626	437.13677	N	557.23143	279.11935	5
8	916.30846	458.65787	944.30338	472.65533	A	443.18850	222.09789	4
9	1053.36737	527.18732	1081.36229	541.18478	H	372.15138	186.57933	3
10	1140.39940	570.70334	1168.39432	584.70080	S	235.09247	118.04987	2
11					E	148.06044	74.53386	1

Fragment Spectrum

Extracted from: C:\Users\SAH\Desktop\Data\Suraya\V2085_EBA181_RH4_tryp_chymo\V2087_RH4_P_tryp_200fmol_43C_70m09233648H4C4.raw #1396 RT: 18.46
FTMS, HCD, z=+2, Mono m/z=658.22736 Da, MH+=1315.44744 Da, Match Tol.=20 mmu

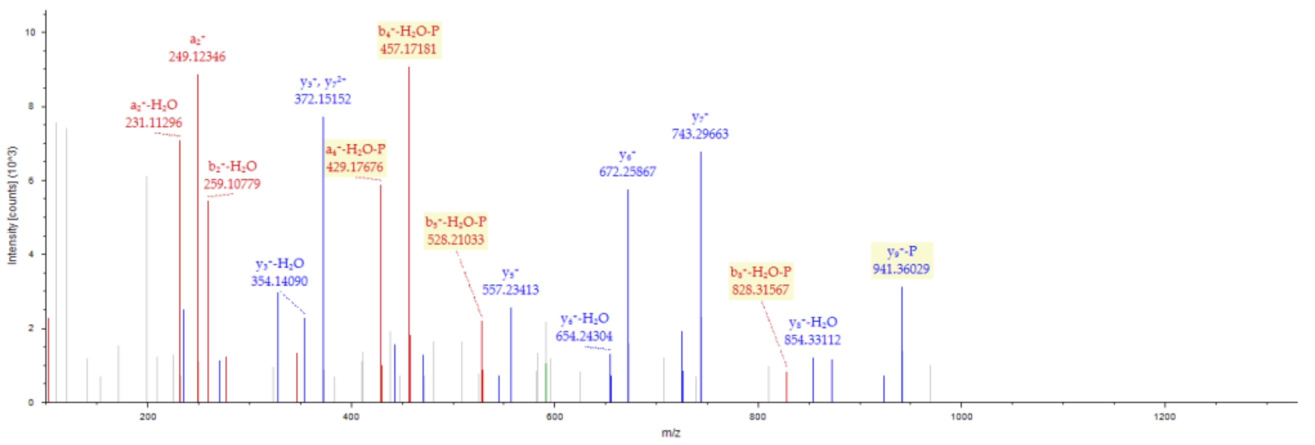

Sequence: EFSEADNAHSE
S3-Phospho (79.96633 Da)
Charge: +2, Monoisotopic m/z: 658.22736 Da (-0.02 mmu/-0.04 ppm), MH+: 1315.44744 Da, RT: 18.46 min,

Extracted from: C:\Users\SAH\Desktop\Data\Suraya\V2085_EBA181_RH4_tryp_chymo\V2087_RH4_P_tryp_200fmol_43C_70m09233648H4C4.raw #1396 RT: 18.46
FTMS, HCD, z=+2, Mono m/z=658.22736 Da, MH+=1315.44744 Da, Match Tol.=20 mmu

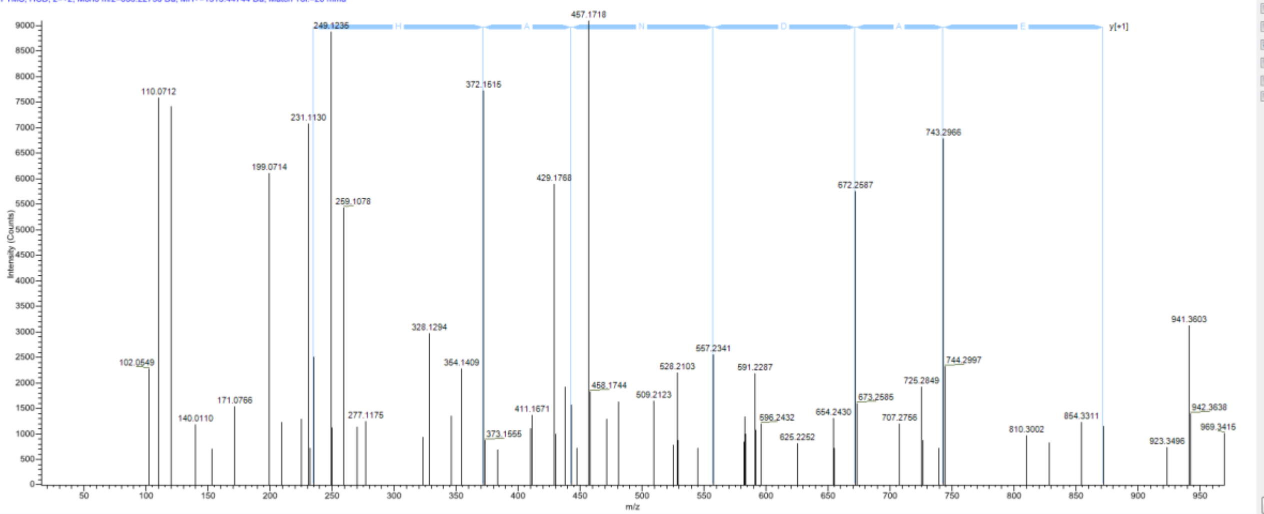

	(NH3+) -	F> <s	s> <E	E>	A>	D>	N>	A>	H> <S	S> <E	E - (COOH)
b (+1)	130.04988	277.11830	444.11666	573.15926	644.19638	759.22333	873.26626	944.30338	1081.36229	1168.39432	1297.43692
b*(+1)	113.02333	260.09175	427.09011	556.13271	627.16983	742.19678	856.23971	927.27683	1064.33574	1151.36777	1280.41037
b°(+1)	112.03931	259.10773	426.10609	555.14869	626.18581	741.21276	855.25569	926.29281	1063.35172	1150.38375	1279.42635
y (+1)	1186.40488	1039.33646	872.33810	743.29550	672.25838	557.23143	443.18850	372.15138	235.09247	148.06044	1315.44748
y*(+1)	1169.37833	1022.30991	855.31155	726.26895	655.23183	540.20488	426.16195	355.12483	218.06592	131.03389	1298.42093
y°(+1)	1168.39432	1021.32590	854.32754	725.28494	654.24782	539.22087	425.17794	354.14082	217.08191	130.04988	1297.43692

Value Type: Theo. Mass [Da]

E F s E A D N A H S E

D. RH4_3

Peptide Summary

Sequence: FQKEFSEADNAH, S6-Phospho (79.96633 Da)
Charge: +2, Monoisotopic m/z: 751.80066 Da (-0.39 mmu/-0.52 ppm), MH+: 1502.59404 Da, RT: 17.72 min,
Identified with: Mascot (v1.27); IonScore:53, Exp Value:3.5E-005, Ions matched by search engine: 10/124
Fragment match tolerance used for search: 20 mmu

Fragment Matches

Value Type: Theo. Mass [Da]

Ion Series | Phosphorylation Losses | Neutral Losses | Multiple Neutral Losses | Precursor Ions

#1	a^+	a^{2+}	b^+	b^{2+}	Seq.	y^+	y^{2+}	#2
1	120.08078	60.54403	148.07570	74.54149	F			12
2	248.13936	124.57332	276.13428	138.57078	Q	1355.52640	678.26684	11
3	376.23433	188.62080	404.22925	202.61826	K	1227.46782	614.23755	10
4	505.27693	253.14210	533.27185	267.13956	E	1099.37285	550.19006	9
5	652.34535	326.67631	680.34027	340.67377	F	970.33025	485.66876	8
6	819.34371	410.17549	847.33863	424.17295	S-Phospho	823.26183	412.13455	7
7	948.38631	474.69679	976.38123	488.69425	E	656.26347	328.63537	6
8	1019.42343	510.21535	1047.41835	524.21281	A	527.22087	264.11407	5
9	1134.45038	567.72883	1162.44530	581.72629	D	456.18375	228.59551	4
10	1248.49331	624.75029	1276.48823	638.74775	N	341.15680	171.08204	3
11	1319.53043	660.26885	1347.52535	674.26631	A	227.11387	114.06057	2
12					H	156.07675	78.54201	1

Fragment Spectrum

Extracted from: C:\Users\SAH\Desktop\Data\Suraya\V2085_EBA181_RH4_tryp_chymo\V2088_RH4_P_chy_200fmol_43C_70m09233648H4C4.raw #1272 RT: 17.72
FTMS, HCD, z=+2, Mono m/z=751.80066 Da, MH+=1502.59404 Da, Match Tol.=20 mmu

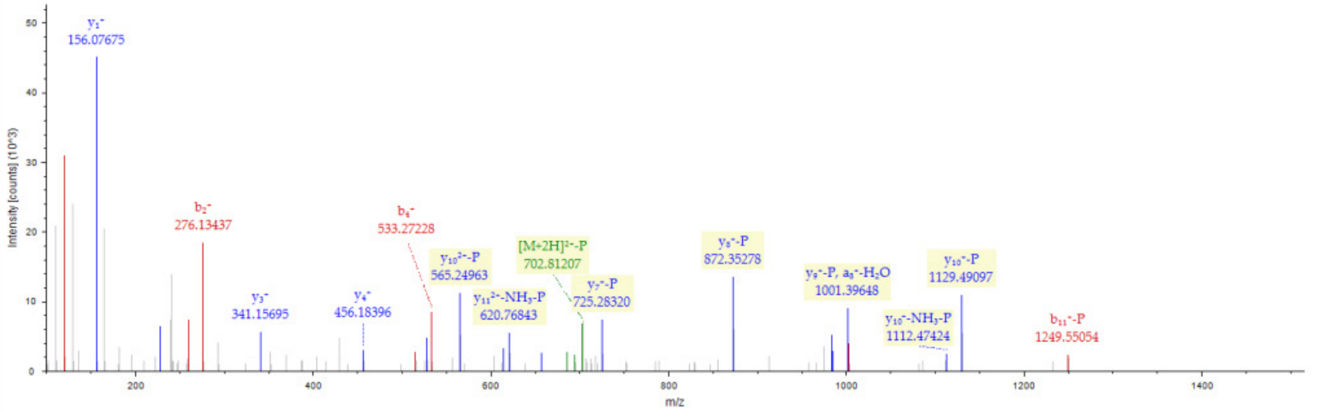

Sequence: FQKEFSEADNAH
S6-Phospho (79.96633 Da)
Charge: +2, Monoisotopic m/z: 751.80066 Da (-0.39 mmu/-0.52 ppm), MH+: 1502.59404 Da, RT: 17.72 min,

Extracted from: C:\Users\SAH\Desktop\Data\Suraya\V2085_EBA181_RH4_tryp_chymo\V2088_RH4_P_chy_200fmol_43C_70m09233648H4C4.raw #1272 RT: 17.72
FTMS, HCD, z=+2, Mono m/z=751.80066 Da, MH+=1502.59404 Da, Match Tol.=20 mmu

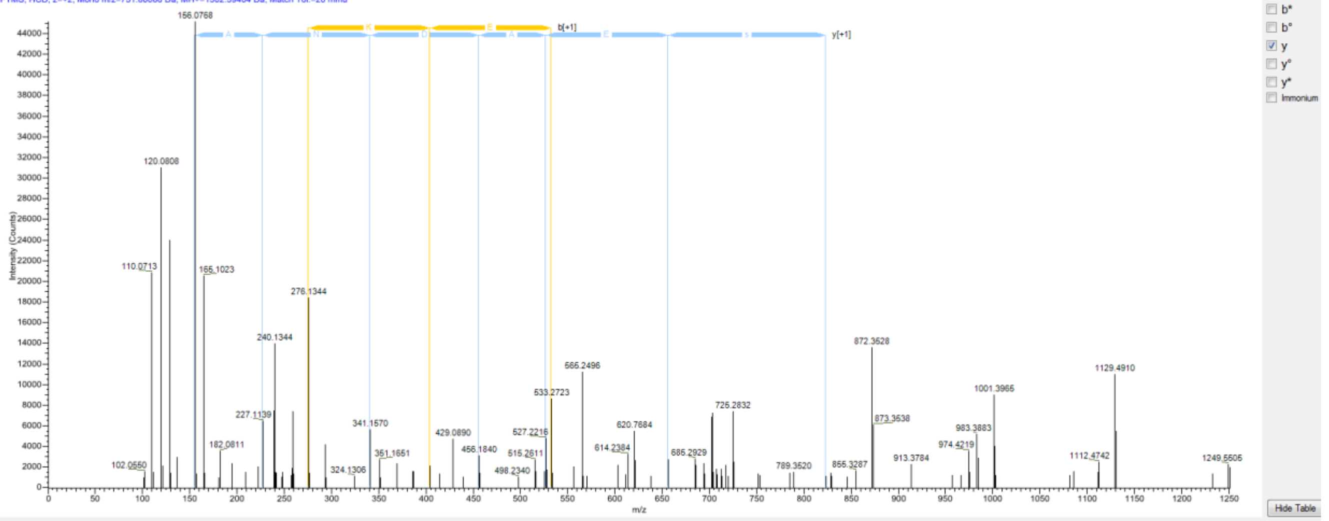

	(NH3+) - F>	Q> <K	K> <E	E> <F	F> <s	s> <E	E>	A> <D	D> <N	N> <A	A> <H	H - (COOH)
b (+1)	148.07570	276.13428	404.22925	533.27185	680.34027	847.33863	976.38123	1047.41835	1162.44530	1276.48823	1347.52535	1484.58426
b*(+1)	131.04915	259.10773	387.20270	516.24530	663.31372	830.31208	959.35468	1030.39180	1145.41875	1259.46168	1330.49880	1467.55771
b°(+1)	130.06513	258.12371	386.21868	515.26128	662.32970	829.32806	958.37066	1029.40778	1144.43473	1258.47766	1329.51478	1466.57369
y (+1)	1355.52640	1227.46782	1099.37285	970.33025	823.26183	656.26347	527.22087	456.18375	341.15680	227.11387	156.07675	1502.59482
y*(+1)	1338.49985	1210.44127	1082.34630	953.30370	806.23528	639.23692	510.19432	439.15720	324.13025	210.08732	139.05020	1485.56827
y°(+1)	1337.51584	1209.45726	1081.36229	952.31969	805.25127	638.25291	509.21031	438.17319	323.14624	209.10331	138.06619	1484.58426

Value Type: Theo. Mass [Da]

F Q K E F s E A D N A H

D. RH4_4

Peptide Summary

Sequence: KEFSEADNAH, S4-Phospho (79.96633 Da)
Charge: +2, Monoisotopic m/z: 614.23749 Da (-0.06 mmu/-0.1 ppm), MH+: 1227.46770 Da, RT: 12.31 min,
Identified with: Mascot (v1.27); IonScore:62, Exp Value:4.9E-006, Ions matched by search engine: 9/104
Fragment match tolerance used for search: 20 mmu

Fragment Matches

Value Type: Theo. Mass [Da]

Ion Series | Phosphorylation Losses | Neutral Losses | Multiple Neutral Losses | Precursor Ions

#1	a⁺	a²⁺	b⁺	b²⁺	Seq.	y⁺	y²⁺	#2
1	101.10733	51.05730	129.10225	65.05476	K			10
2	230.14993	115.57860	258.14485	129.57606	E	1099.37285	550.19006	9
3	377.21835	189.11281	405.21327	203.11027	F	970.33025	485.66876	8
4	544.21671	272.61199	572.21163	286.60945	S-Phospho	823.26183	412.13455	7
5	673.25931	337.13329	701.25423	351.13075	E	656.26347	328.63537	6
6	744.29643	372.65185	772.29135	386.64931	A	527.22087	264.11407	5
7	859.32338	430.16533	887.31830	444.16279	D	456.18375	228.59551	4
8	973.36631	487.18679	1001.36123	501.18425	N	341.15680	171.08204	3
9	1044.40343	522.70535	1072.39835	536.70281	A	227.11387	114.06057	2
10					H	156.07675	78.54201	1

Fragment Spectrum

Extracted from: C:\Users\SAH\Desktop\Data\Suraya\V2085_EBA181_RH4_tryp_chymo\V2088_RH4_P_chy_200fmol_43C_70m09233648H4C4.raw #409 RT: 12.31
FTMS, HCD, z=+2, Mono m/z=614.23749 Da, MH+=1227.46770 Da, Match Tol.=20 mmu

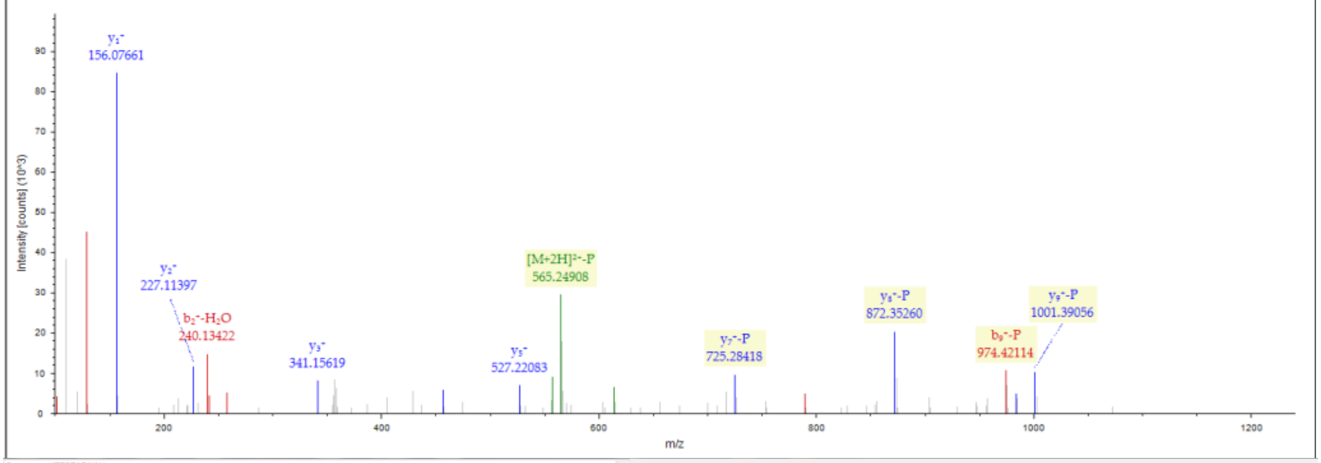

Sequence: KEFSEADNAH
S4-Phospho (79.96633 Da)
Charge: +2, Monoisotopic m/z: 614.23749 Da (-0.06 mmu/-0.1 ppm), MH+: 1227.46770 Da, RT: 12.31 min,

Extracted from: C:\Users\SAH\Desktop\Data\Suraya\V2085_EBA181_RH4_tryp_chymo\V2088_RH4_P_chy_200fmol_43C_70m09233648H4C4.raw #409 RT: 12.31
FTMS, HCD, z=+2, Mono m/z=614.23749 Da, MH+=1227.46770 Da, Match Tol.=20 mmu

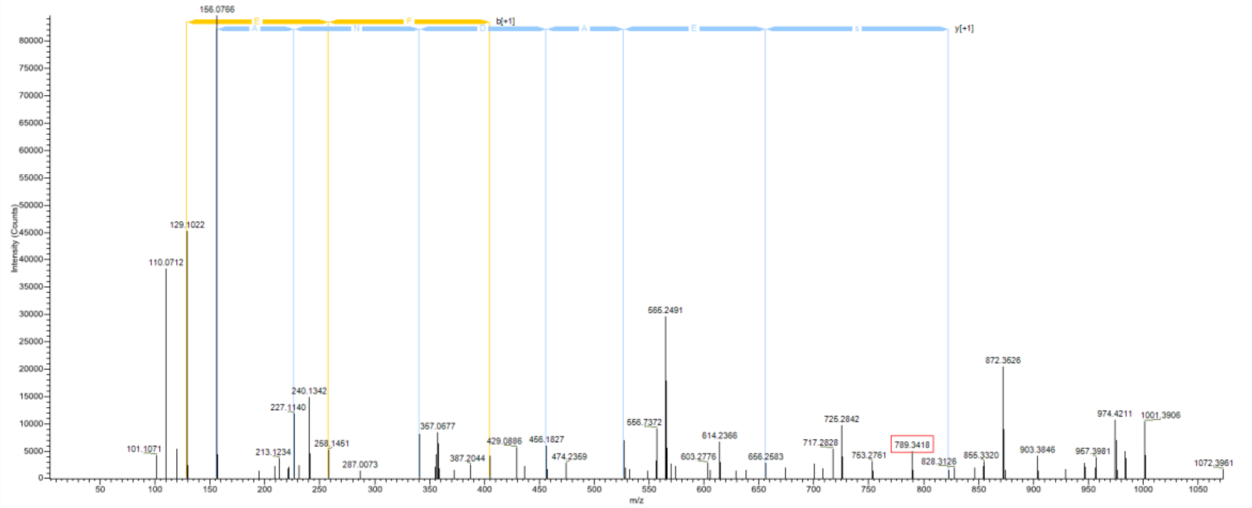

b | b* | b° | y | y° | y* | Immonium

Hide Table

	(NH3+) -	E> <F	F> <s	s> <E	E>	A>	D>	N> <A	A> <H	H - (COOH)
b (+1)	129.10225	258.14485	405.21327	572.21163	701.25423	772.29135	887.31830	1001.36123	1072.39835	1209.45726
b*(+1)	112.07570	241.11830	388.18672	555.18508	684.22768	755.26480	870.29175	984.33468	1055.37180	1192.43071
b°(+1)	111.09168	240.13428	387.20270	554.20106	683.24366	754.28078	869.30773	983.35066	1054.38778	1191.44669
y (+1)	1099.37285	970.33025	823.26183	656.26347	527.22087	456.18375	341.15680	227.11387	156.07675	1227.46782
y*(+1)	1082.34630	953.30370	806.23528	639.23692	510.19432	439.15720	324.13025	210.08732	139.05020	1210.44127
y°(+1)	1081.36229	952.31969	805.25127	638.25291	509.21031	438.17319	323.14624	209.10331	138.06619	1209.45726

Value Type: Theo. Mass [Da]

K E F s E A D N A H

D. RH4_5

Peptide Summary

Sequence: SEADNAHSEEKEEY, S8-Phospho (79.96633 Da)
Charge: +2, Monoisotopic m/z: 859.31451 Da (-0.4 mmu/-0.47 ppm), MH+: 1717.62175 Da, RT: 12.26 min,
Identified with: Mascot (v1.27); IonScore:55, Exp Value:1.7E-005, Ions matched by search engine: 12/134
Fragment match tolerance used for search: 20 mmu

Fragment Matches

Value Type: Theo. Mass [Da]

Ion Series | Phosphorylation Losses | Neutral Losses | Multiple Neutral Losses | Precursor Ions

#1	a^+	a^{2+}	b^+	b^{2+}	Seq.	y^+	y^{2+}	#2
1	60.04439	30.52583	88.03931	44.52329	S			14
2	189.08699	95.04713	217.08191	109.04459	E	1630.59052	815.79890	13
3	260.12411	130.56569	288.11903	144.56315	A	1501.54792	751.27760	12
4	375.15106	188.07917	403.14598	202.07663	D	1430.51080	715.75904	11
5	489.19399	245.10063	517.18891	259.09809	N	1315.48385	658.24556	10
6	560.23111	280.61919	588.22603	294.61665	A	1201.44092	601.22410	9
7	697.29002	349.14865	725.28494	363.14611	H	1130.40380	565.70554	8
8	864.28838	432.64783	892.28330	446.64529	S-Phospho	993.34489	497.17608	7
9	993.33098	497.16913	1021.32590	511.16659	E	826.34653	413.67690	6
10	1122.37358	561.69043	1150.36850	575.68789	E	697.30393	349.15560	5
11	1250.46855	625.73791	1278.46347	639.73537	K	568.26133	284.63430	4
12	1379.51115	690.25921	1407.50607	704.25667	E	440.16636	220.58682	3
13	1508.55375	754.78051	1536.54867	768.77797	E	311.12376	156.06552	2
14					Y	182.08116	91.54422	1

Fragment Spectrum

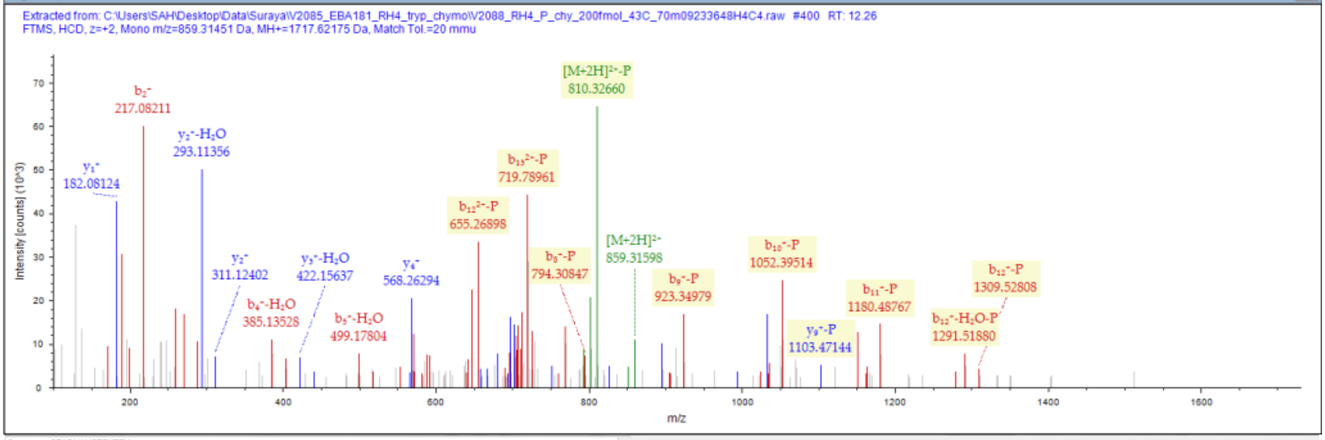

Sequence: SEADNAHSEEKEEY
S8-Phospho (79.96633 Da)
Charge: +2, Monoisotopic m/z: 859.31451 Da (-0.4 mmu/-0.47 ppm), MH+: 1717.62175 Da, RT: 12.26 min,

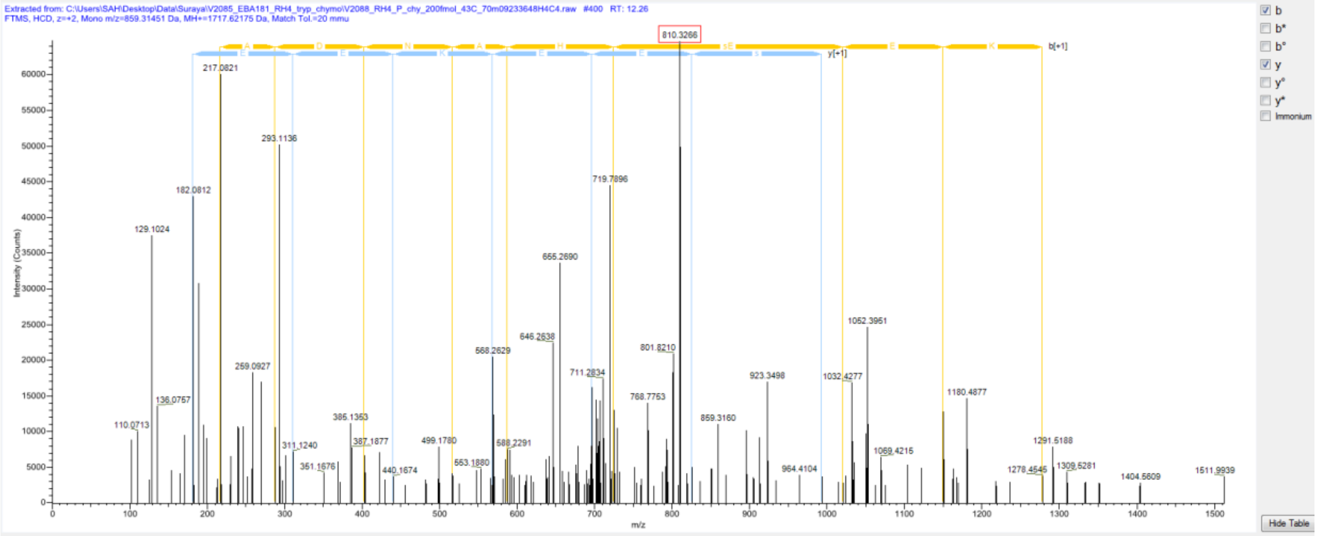

	(NH3+) -	E> <A	A> <D	D> <N	N> <A	A> <H	H> <s	s> <E	E> <E	E> <K	K> <E	E> <E	E> <Y	Y - (COOH)
b (+1)	88.03931	217.08191	288.11903	403.14598	517.18891	588.22603	725.28494	892.28330	1021.32590	1150.36850	1278.46347	1407.50607	1536.54867	1699.61199
b*(+1)	71.01276	200.05536	271.09248	386.11943	500.16236	571.19948	708.25839	875.25675	1004.29935	1133.34195	1261.43692	1390.47952	1519.52212	1682.58544
b°(+1)	70.02874	199.07134	270.10846	385.13541	499.17834	570.21546	707.27437	874.27273	1003.31533	1132.35793	1260.45290	1389.49550	1518.53810	1681.60142
y (+1)	1630.59052	1501.54792	1430.51080	1315.48385	1201.44092	1130.40380	993.34489	826.34653	697.30393	568.26133	440.16636	311.12376	182.08116	1717.62255
y*(+1)	1613.56397	1484.52137	1413.48425	1298.45730	1184.41437	1113.37725	976.31834	809.31998	680.27738	551.23478	423.13981	294.09721	165.05461	1700.59600
y°(+1)	1612.57996	1483.53736	1412.50024	1297.47329	1183.43036	1112.39324	975.33433	808.33597	679.29337	550.25077	422.15580	293.11320	164.07060	1699.61199

Value Type: Theo. Mass [Da]

S E⌋A⌋D⌋N⌋A⌋H⌋**s**⌈E⌋E⌋K⌋E⌈E⌈Y

D. RH4_6

Peptide Summary

Sequence: EFSEADNAHSEEK, S10-Phospho (79.96633 Da)
Charge: +3, Monoisotopic m/z: 524.86652 Da (-0.02 mmu/-0.04 ppm), MH+: 1572.58500 Da, RT: 12.02 min,
Identified with: Mascot (v1.27); IonScore:30, Exp Value:1.1E-003, Ions matched by search engine: 8/120
Fragment match tolerance used for search: 20 mmu

Fragment Matches

Value Type: Theo. Mass [Da]

Ion Series | Phosphorylation Losses | Neutral Losses | Multiple Neutral Losses | Precursor Ions

#1	a⁺	a²⁺	a³⁺	b⁺	b²⁺	b³⁺	Seq.	y⁺	y²⁺	y³⁺	#2
1	102.05496	51.53112	34.68984	130.04988	65.52858	44.02148	E				13
2	249.12338	125.06533	83.71265	277.11830	139.06279	93.04428	F	1443.54245	722.27486	481.85234	12
3	336.15541	168.58134	112.72332	364.15033	182.57880	122.05496	S	1296.47403	648.74065	432.82953	11
4	465.19801	233.10264	155.73752	493.19293	247.10010	165.06916	E	1209.44200	605.22464	403.81885	10
5	536.23513	268.62120	179.41656	564.23005	282.61866	188.74820	A	1080.39940	540.70334	360.80465	9
6	651.26208	326.13468	217.75888	679.25700	340.13214	227.09052	D	1009.36228	505.18478	337.12561	8
7	765.30501	383.15614	255.77319	793.29993	397.15360	265.10483	N	894.33533	447.67130	298.78330	7
8	836.34213	418.67470	279.45223	864.33705	432.67216	288.78387	A	780.29240	390.64984	260.76899	6
9	973.40104	487.20416	325.13853	1001.39596	501.20162	334.47017	H	709.25528	355.13128	237.08995	5
10	1140.39940	570.70334	380.80465	1168.39432	584.70080	390.13629	S-Phospho	572.19637	286.60182	191.40364	4
11	1269.44200	635.22464	423.81885	1297.43692	649.22210	433.15049	E	405.19801	203.10264	135.73752	3
12	1398.48460	699.74594	466.83305	1426.47952	713.74340	476.16469	E	276.15541	138.58134	92.72332	2
13							K	147.11281	74.06004	49.70912	1

Fragment Spectrum

Extracted from: C:\Users\SAH\Desktop\Data\Suraya\V2085_EBA181_RH4_tryp_chymo\V2087_RH4_P_tryp_200fmol_43C_70m09233648H4C4.raw #367 RT: 12.02
FTMS, HCD, z=+3, Mono m/z=524.86652 Da, MH+=1572.58500 Da, Match Tol.=20 mmu

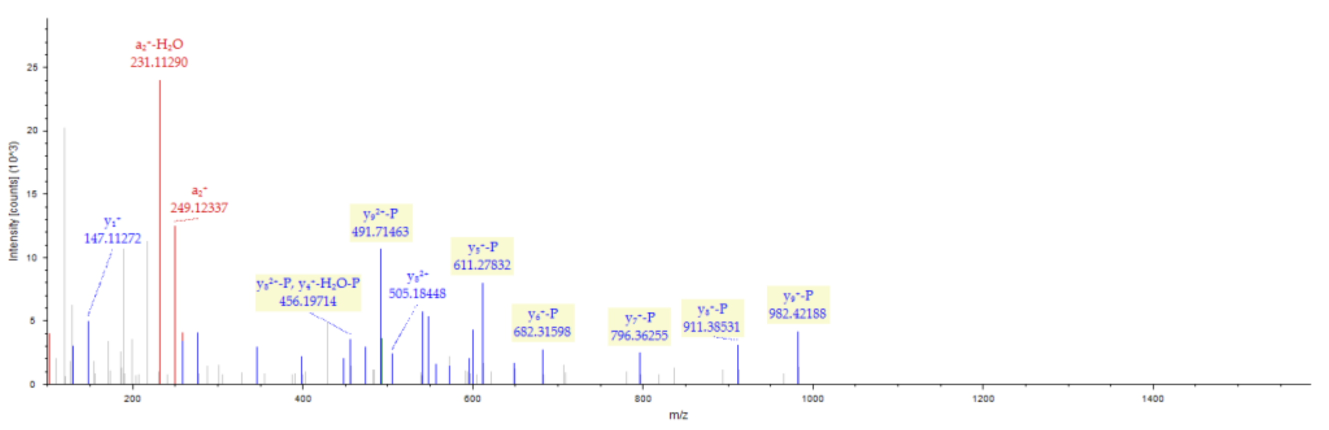

Sequence: EFSEADNAHSEEK
S10-Phospho (79.96633 Da)
Charge: +3, Monoisotopic m/z: 524.86652 Da (-0.02 mmu/-0.04 ppm), MH+: 1572.58500 Da, RT: 12.02 min,

Extracted from: C:\Users\SAH\Desktop\Data\Suraya\V2085_EBA181_RH4_tryp_chymo\V2087_RH4_P_tryp_200fmol_43C_70m09233648H4C4.raw #367 RT: 12.02
FTMS, HCD, z=+3, Mono m/z=524.86652 Da, MH+=1572.58500 Da, Match Tol.=20 mmu

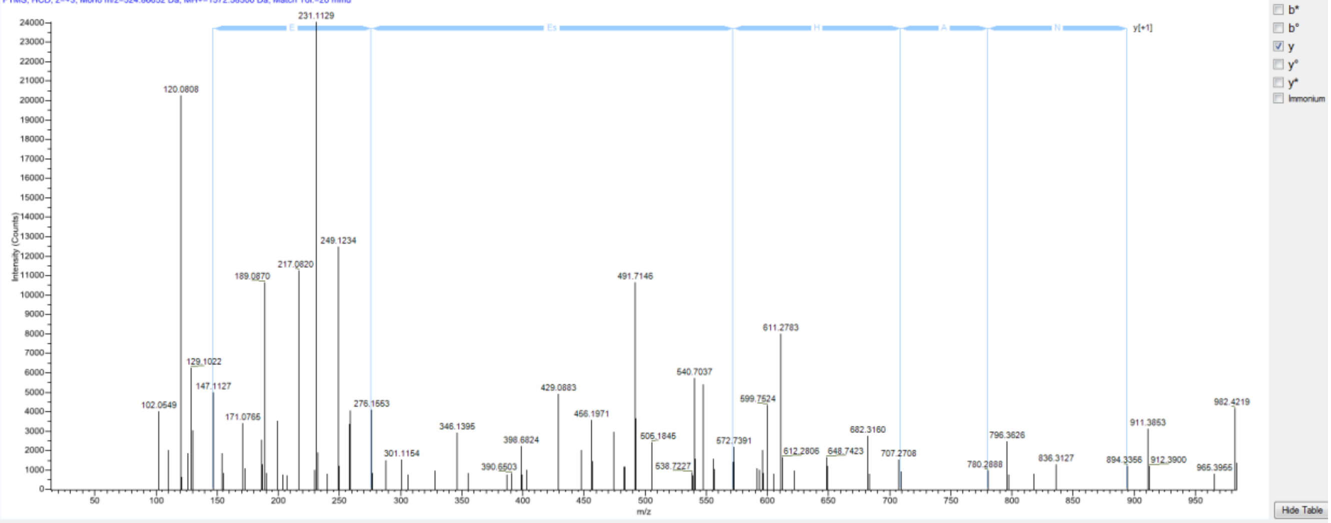

	(NH3+) -	F> <S	S> <E	E> <A	A> <D	D>	N>	A>	H> <s	s> <E	E> <E	E> <K	K - (COOH)
b (+1)	130.04988	277.11830	364.15033	493.19293	564.23005	679.25700	793.29993	864.33705	1001.39596	1168.39432	1297.43692	1426.47952	1554.57449
b*(+1)	113.02333	260.09175	347.12378	476.16638	547.20350	662.23045	776.27338	847.31050	984.36941	1151.36777	1280.41037	1409.45297	1537.54794
b°(+1)	112.03931	259.10773	346.13976	475.18236	546.21948	661.24643	775.28936	846.32648	983.38539	1150.38375	1279.42635	1408.46895	1536.56392
y (+1)	1443.54245	1296.47403	1209.44200	1080.39940	1009.36228	894.33533	780.29240	709.25528	572.19637	405.19801	276.15541	147.11281	1572.58505
y*(+1)	1426.51590	1279.44748	1192.41545	1063.37285	992.33573	877.30878	763.26585	692.22873	555.16982	388.17146	259.12886	130.08626	1555.55850
y°(+1)	1425.53189	1278.46347	1191.43144	1062.38884	991.35172	876.32477	762.28184	691.24472	554.18581	387.18745	258.14485	129.10225	1554.57449

Value Type: Theo. Mass [Da]

E F⌋S E A D⌈N⌈A⌈H⌈**s** E⌈E⌈K

D. RH4_7

Peptide Summary

Sequence: AHSEEKEEY, S3-Phospho (79.96633 Da)
Charge: +2, Monoisotopic m/z: 601.22388 Da (-0.22 mmu/-0.37 ppm), MH+: 1201.44048 Da, RT: 11.08 min,
Identified with: Mascot (v1.27); IonScore:20, Exp Value:4.8E-002, Ions matched by search engine: 7/70
Fragment match tolerance used for search: 20 mmu

Fragment Matches

Value Type: Theo. Mass [Da]

Ion Series | Phosphorylation Losses | Neutral Losses | Multiple Neutral Losses | Precursor Ions

#1	a⁺	a²⁺	b⁺	b²⁺	Seq.	y⁺	y²⁺	#2
1	44.04948	22.52838	72.04440	36.52584	A			9
2	181.10839	91.05783	209.10331	105.05529	H	1130.40380	565.70554	8
3	348.10675	174.55701	376.10167	188.55447	S-Phospho	993.34489	497.17608	7
4	477.14935	239.07831	505.14427	253.07577	E	826.34653	413.67690	6
5	606.19195	303.59961	634.18687	317.59707	E	697.30393	349.15560	5
6	734.28692	367.64710	762.28184	381.64456	K	568.26133	284.63430	4
7	863.32952	432.16840	891.32444	446.16586	E	440.16636	220.58682	3
8	992.37212	496.68970	1020.36704	510.68716	E	311.12376	156.06552	2
9					Y	182.08116	91.54422	1

Fragment Spectrum

Extracted from: C:\Users\SAH\Desktop\Data\Suraya\V2085_EBA181_RH4_tryp_chymo\V2088_RH4_P_chy_200fmol_43C_70m09233648H4C4.raw #219 RT: 11.08
FTMS, HCD, z=+2, Mono m/z=601.22388 Da, MH+=1201.44048 Da, Match Tol.=20 mmu

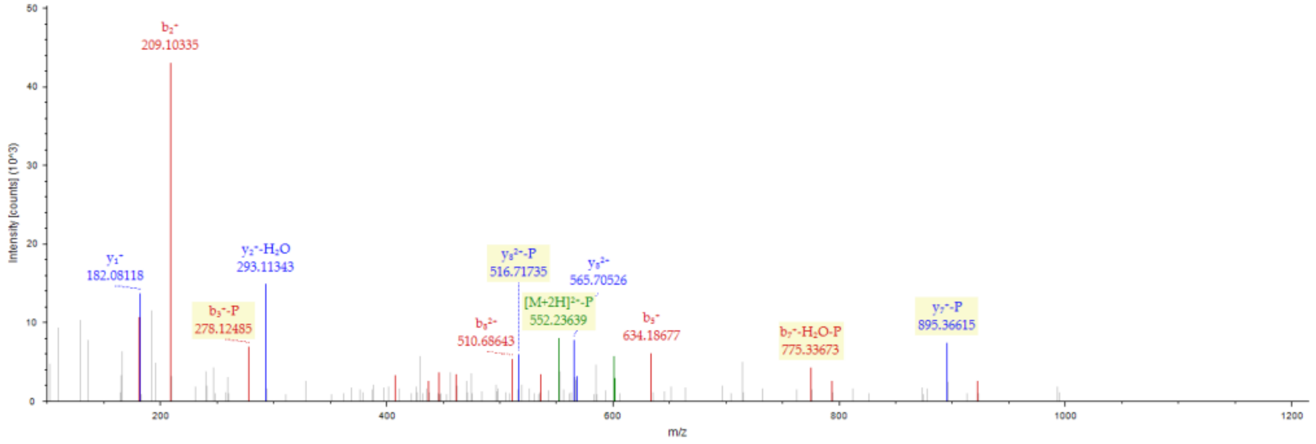

Sequence: AHSEEKEEY
S3-Phospho (79.96633 Da)
Charge: +2, Monoisotopic m/z: 601.22388 Da (-0.22 mmu/-0.37 ppm), MH+: 1201.44048 Da, RT: 11.08 min.

Extracted from: C:\Users\SAH\Desktop\Data\Suraya\V2085_EBA181_RH4_tryp_chymo\V2088_RH4_P_chy_200fmol_43C_70m09233648H4C4.raw #219 RT: 11.08
FTMS, HCD, z=+2, Mono m/z=601.22388 Da, MH+=1201.44048 Da, Match Tol.=20 mmu

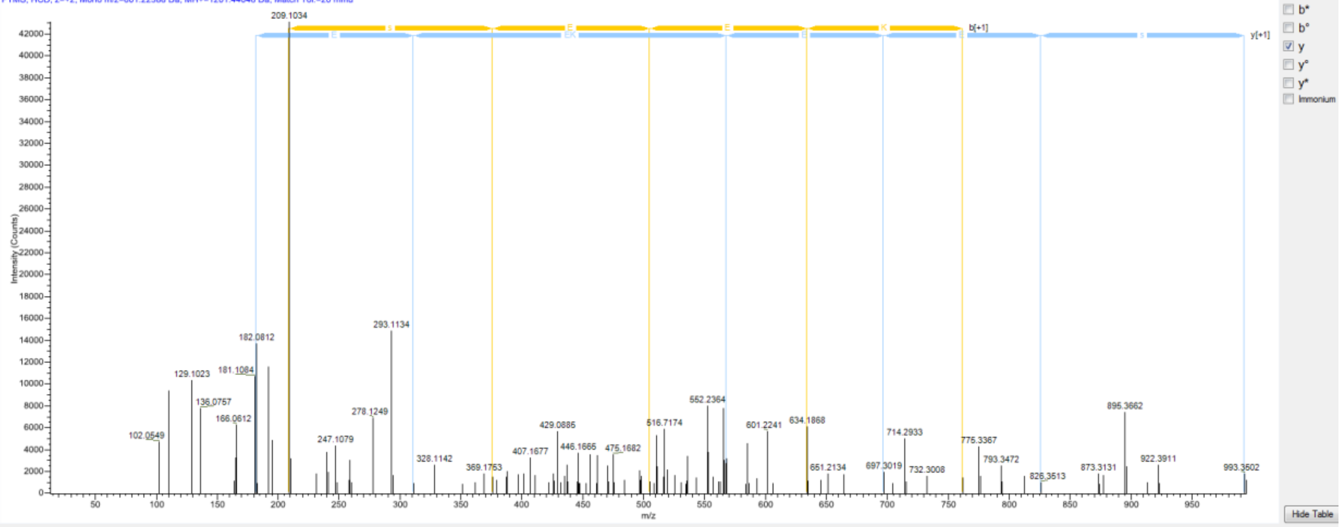

	(NH3+) -	H> <s	s> <E	E>	E>	K>	E>	E> <Y	Y - (COOH)
b (+1)	72.04440	209.10331	376.10167	505.14427	634.18687	762.28184	891.32444	1020.36704	1183.43036
b*(+1)	55.01785	192.07676	359.07512	488.11772	617.16032	745.25529	874.29789	1003.34049	1166.40381
b°(+1)	54.03383	191.09274	358.09110	487.13370	616.17630	744.27127	873.31387	1002.35647	1165.41979
y (+1)	1130.40380	993.34489	826.34653	697.30393	568.26133	440.16636	311.12376	182.08116	1201.44092
y*(+1)	1113.37725	976.31834	809.31998	680.27738	551.23478	423.13981	294.09721	165.05461	1184.41437
y°(+1)	1112.39324	975.33433	808.33597	679.29337	550.25077	422.15580	293.11320	164.07060	1183.43036

Value Type: Theo. Mass [Da]

A H s E E K E E Y

E. EBA140_1

```
[226.0776]RMGKSNEEYDIGESNIEATFEENNYLNKLSRIFNQEVQETNISDYSEYNYNEKNMY
      Number of Amino Acids:  56
           Theoretical Mass:  6992.11 Da
            Mass Difference:  261.89 Da
B Ions:  13     Y Ions:  3
```

Ion	Observed Mass (Da)	Theoretical Mass (Da)	Mass Error (Da)	Mass Error (PPM)	Delta M
Y12	1616.6393	1616.6473	-0.0080	-4.93	--
Y9	1237.5040	1237.5093	-0.0054	-4.32	--
Y8	1074.4401	1074.4460	-0.0059	-5.46	--
B19	2350.0138	2350.0205	-0.0067	-2.85	--
B18	2248.9671	2248.9729	-0.0057	-2.55	--
B17	2177.9296	2177.9358	-0.0061	-2.81	--
B16	2048.8871	2048.8932	-0.0061	-2.97	--
B15	1935.8032	1935.8091	-0.0059	-3.04	--
B14	1821.7605	1821.7662	-0.0056	-3.09	--
B13	1734.7291	1734.7341	-0.0051	-2.92	--
B12	1605.6871	1605.6915	-0.0045	-2.79	--
B11	1548.6656	1548.6701	-0.0045	-2.91	--
B10	1435.5818	1435.5860	-0.0042	-2.96	--
B9	1320.5553	1320.5591	-0.0038	-2.91	--
B8	1157.4917	1157.4958	-0.0041	-3.53	--
B7	1028.4504	1028.4532	-0.0027	-2.66	--

Crude PScore: 6.00565e-13
McLuckey Score: 14.8718

Graphical Fragment Mapper

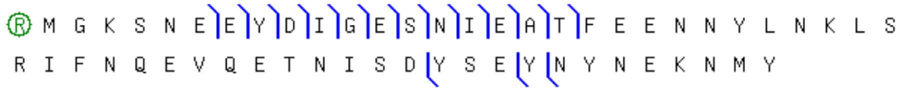

E. EBA140_2

```
[226.0776]RMGKSNEEYDIGESNIEATFEENNYLNKLSRIFNQEVQETNISDYSEYNYNEKNMY
      Number of Amino Acids:  56
           Theoretical Mass:  6992.11 Da
            Mass Difference:  261.89 Da
B Ions:  16     Y Ions:  1
```

Ion	Observed Mass (Da)	Theoretical Mass (Da)	Mass Error (Da)	Mass Error (PPM)	Delta M
Y8	1074.4408	1074.4460	-0.0052	-4.83	--
B19	2350.0142	2350.0205	-0.0064	-2.71	--
B18	2248.9674	2248.9729	-0.0055	-2.44	--
B17	2177.9300	2177.9358	-0.0058	-2.65	--
B16	2048.8873	2048.8932	-0.0059	-2.86	--
B15	1935.8033	1935.8091	-0.0058	-2.98	--
B14	1821.7610	1821.7662	-0.0052	-2.85	--
B13	1734.7292	1734.7341	-0.0050	-2.87	--
B12	1605.6871	1605.6915	-0.0044	-2.76	--
B11	1548.6656	1548.6701	-0.0045	-2.87	--
B10	1435.5817	1435.5860	-0.0043	-3.03	--
B9	1320.5554	1320.5591	-0.0037	-2.79	--
B8	1157.4919	1157.4958	-0.0039	-3.34	--
B7	1028.4507	1028.4532	-0.0025	-2.42	--
B6	899.4078	899.4106	-0.0028	-3.13	--
B4	698.3336	698.3356	-0.0020	-2.89	--
B1	382.1777	382.1787	-0.0010	-2.66	--

Crude PScore: 1.86896e-14
McLuckey Score: 15.3005

Graphical Fragment Mapper

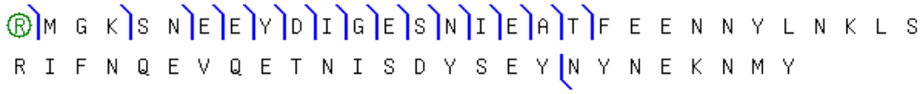

F. EBA175_1

VQE[79.96633]TNINDFSEYHEDINDINFKK
Number of Amino Acids: 23
Theoretical Mass: 2891.27 Da
Mass Difference: -0.067 Da
B Ions: 8 Y Ions: 21

Ion	Observed Mass (Da)	Theoretical Mass (Da)	Mass Error (Da)	Mass Error (PPM)	Delta M
Y22	2792.1806	2792.1986	-0.0180	-6.46	--
	2792.1448	2792.1986	-0.0538	-19.25	--
Y21	2664.1216	2664.1400	-0.0184	-6.92	--
Y20	2535.0794	2535.0974	-0.0180	-7.10	--
Y19	2354.0658	2354.0834	-0.0176	-7.46	--
Y18	2240.0237	2240.0405	-0.0168	-7.51	--
Y17	2126.9407	2126.9564	-0.0158	-7.41	--
Y16	2012.8981	2012.9135	-0.0154	-7.63	--
Y15	1897.8727	1897.8866	-0.0138	-7.28	--
Y14	1750.8050	1750.8181	-0.0132	-7.52	--
Y13	1663.7742	1663.7861	-0.0119	-7.14	--
Y12	1534.7322	1534.7435	-0.0113	-7.35	--
Y11	1371.6692	1371.6802	-0.0110	-8.04	--
Y10	1234.6110	1234.6213	-0.0102	-8.29	--
Y9	1105.5694	1105.5787	-0.0093	-8.38	--
Y8	990.5432	990.5517	-0.0086	-8.63	--
Y7	877.4601	877.4677	-0.0075	-8.60	--
Y6	763.4179	763.4248	-0.0069	-9.01	--
Y5	648.3916	648.3978	-0.0063	-9.67	--
Y4	535.3082	535.3138	-0.0055	-10.37	--
Y3	421.2663	421.2708	-0.0045	-10.74	--
Y2	274.1987	274.2024	-0.0037	-13.40	--
B18	2242.8546	2242.8692	-0.0145	-6.49	--
B13	1656.6354	1656.6457	-0.0103	-6.22	--
B11	1356.5151	1356.5235	-0.0083	-6.15	--
B9	1140.4420	1140.4489	-0.0068	-5.99	--
B6	764.3057	764.3106	-0.0049	-6.41	--
B5	651.2222	651.2265	-0.0043	-6.57	--
B3	356.1675	356.1696	-0.0021	-5.97	--
B2	227.1255	227.1270	-0.0015	-6.53	--

Crude PScore: 1.17369e-32
McLuckey Score: 13.8965

Graphical Fragment Mapper

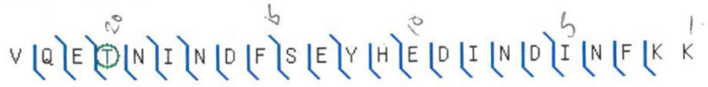

F. EBA175_2

```
VQETNINDF[79.96633]SEYHEDINDINFKK
      Number of Amino Acids:  23
           Theoretical Mass:  2891.27 Da
            Mass Difference:  -0.067 Da
B Ions:  5      Y Ions:  17
```

Ion	Observed Mass (Da)	Theoretical Mass (Da)	Mass Error (Da)	Mass Error (PPM)	Delta M
Y22	2792.1806	2792.1986	-0.0180	-6.46	--
	2792.1448	2792.1986	-0.0538	-19.25	--
Y21	2664.1216	2664.1400	-0.0184	-6.92	--
Y20	2535.0794	2535.0974	-0.0180	-7.10	--
Y16	2092.8983	2092.8798	0.0185	8.84	--
Y15	1977.8621	1977.8529	0.0092	4.65	--
Y13	1663.7742	1663.7861	-0.0119	-7.14	--
Y12	1534.7322	1534.7435	-0.0113	-7.35	--
Y11	1371.6692	1371.6802	-0.0110	-8.04	--
Y10	1234.6110	1234.6213	-0.0102	-8.29	--
Y9	1105.5694	1105.5787	-0.0093	-8.38	--
Y8	990.5432	990.5517	-0.0086	-8.63	--
Y7	877.4601	877.4677	-0.0075	-8.60	--
Y6	763.4179	763.4248	-0.0069	-9.01	--
Y5	648.3916	648.3978	-0.0063	-9.67	--
Y4	535.3082	535.3138	-0.0055	-10.37	--
Y3	421.2663	421.2708	-0.0045	-10.74	--
Y2	274.1987	274.2024	-0.0037	-13.40	--
B18	2242.8546	2242.8692	-0.0145	-6.49	--
B13	1656.6354	1656.6457	-0.0103	-6.22	--
B11	1356.5151	1356.5235	-0.0083	-6.15	--
B3	356.1675	356.1696	-0.0021	-5.97	--
B2	227.1255	227.1270	-0.0015	-6.53	--

Crude PScore: 1.28965e-22
McLuckey Score: 11.9891

Graphical Fragment Mapper

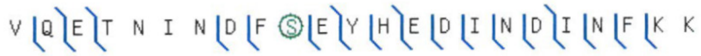

G. EBA181_1

Peptide Summary

Sequence: YDSNLNDSAFEYNNNK, S3-Phospho (79.96633 Da)
Charge: +2, Monoisotopic m/z: 994.38861 Da (-0.33 mmu/-0.33 ppm), MH+: 1987.76995 Da, RT: 35.80 min,
Identified with: Mascot (v1.27); IonScore:81, Exp Value:1.2E-008, Ions matched by search engine: 11/168
Fragment match tolerance used for search: 20 mmu

Fragment Matches

Value Type: Theo. Mass [Da]

Ion Series | Phosphorylation Losses | Neutral Losses | Multiple Neutral Losses | Precursor Ions

#1	a⁺	a²⁺	b⁺	b²⁺	Seq.	y⁺	y²⁺	#2
1	136.07568	68.54148	164.07060	82.53894	Y			16
2	251.10263	126.05495	279.09755	140.05241	D	1824.70728	912.85728	15
3	418.10099	209.55413	446.09591	223.55159	S-Phospho	1709.68033	855.34380	14
4	532.14392	266.57560	560.13884	280.57306	N	1542.68197	771.84462	13
5	645.22799	323.11763	673.22291	337.11509	L	1428.63904	714.82316	12
6	759.27092	380.13910	787.26584	394.13656	N	1315.55497	658.28112	11
7	874.29787	437.65257	902.29279	451.65003	D	1201.51204	601.25966	10
8	961.32990	481.16859	989.32482	495.16605	S	1086.48509	543.74618	9
9	1032.36702	516.68715	1060.36194	530.68461	A	999.45306	500.23017	8
10	1179.43544	590.22136	1207.43036	604.21882	F	928.41594	464.71161	7
11	1308.47804	654.74266	1336.47296	668.74012	E	781.34752	391.17740	6
12	1471.54136	736.27432	1499.53628	750.27178	Y	652.30492	326.65610	5
13	1585.58429	793.29578	1613.57921	807.29324	N	489.24160	245.12444	4
14	1699.62722	850.31725	1727.62214	864.31471	N	375.19867	188.10297	3
15	1813.67015	907.33871	1841.66507	921.33617	N	261.15574	131.08151	2
16					K	147.11281	74.06004	1

Fragment Spectrum

Extracted from: C:\Users\SAH\Desktop\Data\Suraya\V2085_EBA181_RH4_tryp_chymo\V2085_EBA181_P_tryp_2pmols_43C_70m09233648H4C4.raw #5404 RT: 35.80
FTMS, HCD, z=+2, Mono m/z=994.38861 Da, MH+=1987.76995 Da, Match Tol.=20 mmu

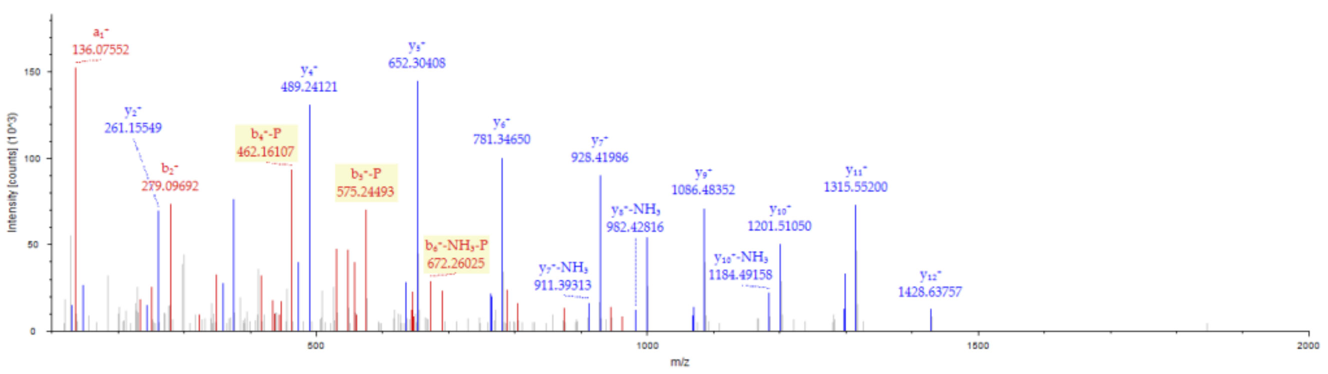

Sequence: YDSNLNDSAFEYNNNK
S3-Phospho (79.96633 Da)
Charge: +2, Monoisotopic m/z: 994.38861 Da (-0.33 mmu/-0.33 ppm), MH+: 1987.76995 Da, RT: 35.80 min,

Extracted from: C:\Users\SAH\Desktop\Data\Suraya\V2085_EBA181_RH4_tryp_chymo\V2085_EBA181_P_tryp_2pmols_43C_70m09233648H4C4.raw #5404 RT: 35.80
FTMS, HCD, z=+2, Mono m/z=994.38861 Da, MH+=1987.76995 Da, Match Tol.=20 mmu

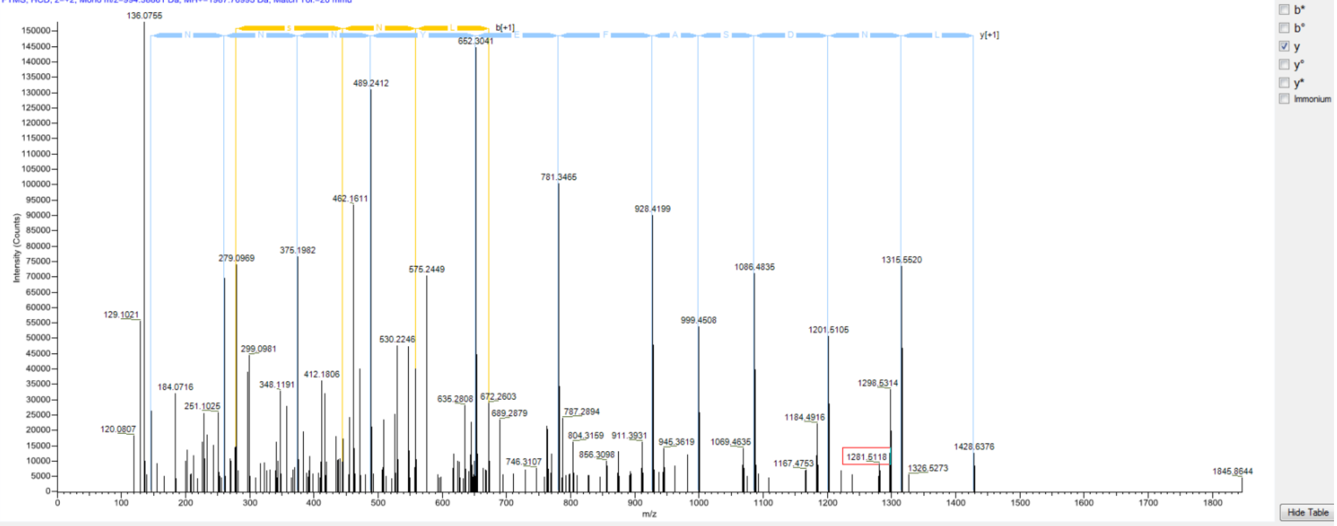

	(NH3+) -	D> <s	s> <N	N> <L	L> <N	N> <D	D> <S	S>	A> <F	F> <E	E> <Y	Y> <N	N> <N	N> <N	N> <K	K - (COOH)
b (+1)	164.07060	279.09755	446.09591	560.13884	673.22291	787.26584	902.29279	989.32482	1060.36194	1207.43036	1336.47296	1499.53628	1613.57921	1727.62214	1841.66507	1969.76004
b*(+1)	147.04405	262.07100	429.06936	543.11229	656.19636	770.23929	885.26624	972.29827	1043.33539	1190.40381	1319.44641	1482.50973	1596.55266	1710.59559	1824.63852	1952.73349
b°(+1)	146.06003	261.08698	428.08534	542.12827	655.21234	769.25527	884.28222	971.31425	1042.35137	1189.41979	1318.46239	1481.52571	1595.56864	1709.61157	1823.65450	1951.74947
y (+1)	1824.70728	1709.68033	1542.68197	1428.63904	1315.55497	1201.51204	1086.48509	999.45306	928.41594	781.34752	652.30492	489.24160	375.19867	261.15574	147.11281	1987.77060
y*(+1)	1807.68073	1692.65378	1525.65542	1411.61249	1298.52842	1184.48549	1069.45854	982.42651	911.38939	764.32097	635.27837	472.21505	358.17212	244.12919	130.08626	1970.74405
y°(+1)	1806.69672	1691.66977	1524.67141	1410.62848	1297.54441	1183.50148	1068.47453	981.44250	910.40538	763.33696	634.29436	471.23104	357.18811	243.14518	129.10225	1969.76004

Value Type: Theo. Mass [Da]

Y D s N L N D S A F E Y N N N K

G. EBA181_2

Peptide Summary

Sequence: SNLNDSAFEYNNNK, S1-Phospho (79.96633 Da)
Charge: +2, Monoisotopic m/z: 855.34296 Da (-0.85 mmu/-0.99 ppm), MH+: 1709.67864 Da, RT: 30.40 min,
Identified with: Mascot (v1.27); IonScore:81, Exp Value:8.4E-009, Ions matched by search engine: 8/152
Fragment match tolerance used for search: 20 mmu

Fragment Matches

Value Type: Theo. Mass [Da]

Ion Series | Phosphorylation Losses | Neutral Losses | Multiple Neutral Losses | Precursor Ions

#1	a⁺	a²⁺	b⁺	b²⁺	Seq.	y⁺	y²⁺	#2
1	140.01072	70.50900	168.00564	84.50646	S-Phospho			14
2	254.05365	127.53046	282.04857	141.52792	N	1542.68197	771.84462	13
3	367.13772	184.07250	395.13264	198.06996	L	1428.63904	714.82316	12
4	481.18065	241.09396	509.17557	255.09142	N	1315.55497	658.28112	11
5	596.20760	298.60744	624.20252	312.60490	D	1201.51204	601.25966	10
6	683.23963	342.12345	711.23455	356.12091	S	1086.48509	543.74618	9
7	754.27675	377.64201	782.27167	391.63947	A	999.45306	500.23017	8
8	901.34517	451.17622	929.34009	465.17368	F	928.41594	464.71161	7
9	1030.38777	515.69752	1058.38269	529.69498	E	781.34752	391.17740	6
10	1193.45109	597.22918	1221.44601	611.22664	Y	652.30492	326.65610	5
11	1307.49402	654.25065	1335.48894	668.24811	N	489.24160	245.12444	4
12	1421.53695	711.27211	1449.53187	725.26957	N	375.19867	188.10297	3
13	1535.57988	768.29358	1563.57480	782.29104	N	261.15574	131.08151	2
14					K	147.11281	74.06004	1

Fragment Spectrum

Extracted from: C:\Users\SAH\Desktop\Data\Suraya\V2085_EBA181_RH4_tryp_chymo\V2085_EBA181_P_tryp_2pmols_43C_70m09233648H4C4.raw #4224 RT: 30.40
FTMS, HCD, z=+2, Mono m/z=855.34296 Da, MH+=1709.67864 Da, Match Tol.=20 mmu

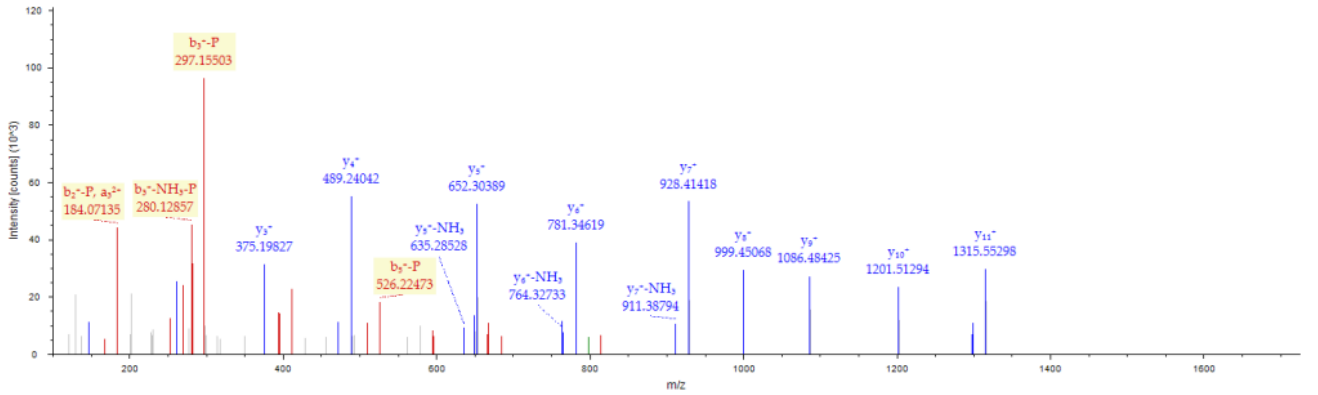

Sequence: SNLNDSAFEYNNNK
S1-Phospho (79.96633 Da)
Charge: +2, Monoisotopic m/z: 855.34296 Da (-0.85 mmu/-0.99 ppm), MH+: 1709.67864 Da, RT: 30.40 min,

Extracted from: C:\Users\SAH\Desktop\Data\Suraya\V2085_EBA181_RH4_tryp_chymo\V2085_EBA181_P_tryp_2pmols_43C_70m09233648H4C4.raw #4224 RT: 30.40
FTMS, HCD, z=+2, Mono m/z=855.34296 Da, MH+=1709.67864 Da, Match Tol.=20 mmu

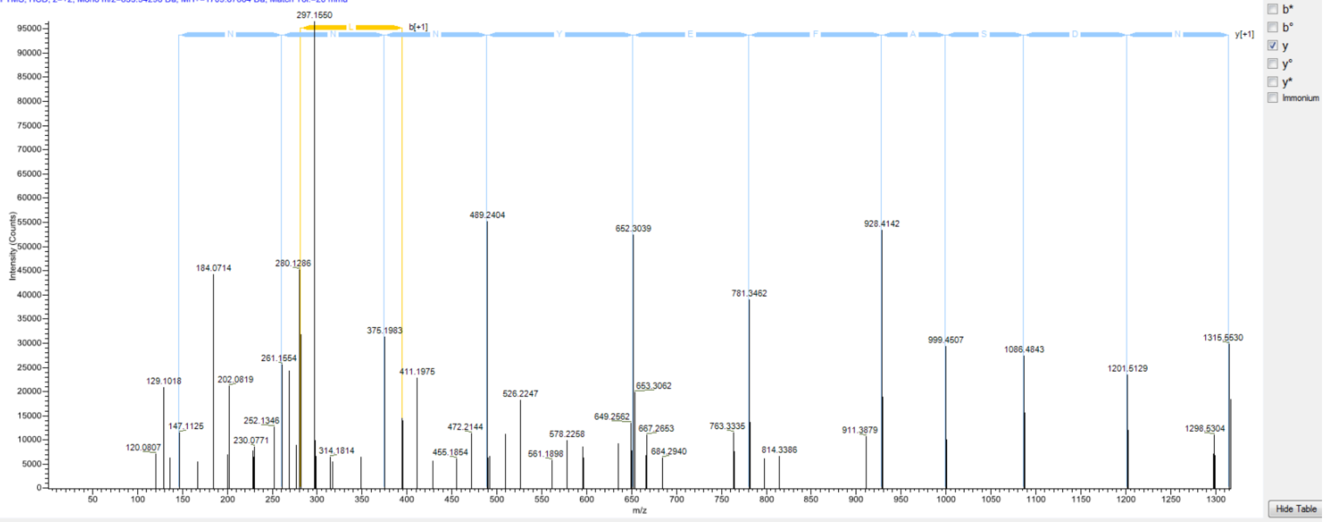

	(NH3+) - s>	N> <L	L> <N	N> <D	D> <S	S>	A> <F	F> <E	E> <Y	Y> <N	N> <N	N> <N	N> <K	K - (COOH)
b (+1)	168.00564	282.04857	395.13264	509.17557	624.20252	711.23455	782.27167	929.34009	1058.38269	1221.44601	1335.48894	1449.53187	1563.57480	1691.66977
b*(+1)	150.97909	265.02202	378.10609	492.14902	607.17597	694.20800	765.24512	912.31354	1041.35614	1204.41946	1318.46239	1432.50532	1546.54825	1674.64322
b°(+1)	149.99507	264.03800	377.12207	491.16500	606.19195	693.22398	764.26110	911.32952	1040.37212	1203.43544	1317.47837	1431.52130	1545.56423	1673.65920
y (+1)	1542.68197	1428.63904	1315.55497	1201.51204	1086.48509	999.45306	928.41594	781.34752	652.30492	489.24160	375.19867	261.15574	147.11281	1709.68033
y*(+1)	1525.65542	1411.61249	1298.52842	1184.48549	1069.45854	982.42651	911.38939	764.32097	635.27837	472.21505	358.17212	244.12919	130.08626	1692.65378
y°(+1)	1524.67141	1410.62848	1297.54441	1183.50148	1068.47453	981.44250	910.40538	763.33696	634.29436	471.23104	357.18811	243.14518	129.10225	1691.66977

Value Type: Theo. Mass [Da]

s N L N D S A F E Y N N N K

G. EBA181_3

Peptide Summary

Close

Sequence: DSNLNDSAFEYNNNK, S2-Phospho (79.96633 Da)
Charge: +2, Monoisotopic m/z: 912.85657 Da (-0.71 mmu/-0.78 ppm), MH+: 1824.70586 Da, RT: 33.82 min,
Identified with: Mascot (v1.27); IonScore:62, Exp Value:8.9E-007, Ions matched by search engine: 9/160
Fragment match tolerance used for search: 20 mmu

Fragment Matches

Value Type: Theo. Mass [Da]

Ion Series | Phosphorylation Losses | Neutral Losses | Multiple Neutral Losses | Precursor Ions

#1	a⁺	a²⁺	b⁺	b²⁺	Seq.	y⁺	y²⁺	#2
1	88.03931	44.52329	116.03423	58.52075	D			15
2	255.03767	128.02247	283.03259	142.01993	S-Phospho	1709.68033	855.34380	14
3	369.08060	185.04394	397.07552	199.04140	N	1542.68197	771.84462	13
4	482.16467	241.58597	510.15959	255.58343	L	1428.63904	714.82316	12
5	596.20760	298.60744	624.20252	312.60490	N	1315.55497	658.28112	11
6	711.23455	356.12091	739.22947	370.11837	D	1201.51204	601.25966	10
7	798.26658	399.63693	826.26150	413.63439	S	1086.48509	543.74618	9
8	869.30370	435.15549	897.29862	449.15295	A	999.45306	500.23017	8
9	1016.37212	508.68970	1044.36704	522.68716	F	928.41594	464.71161	7
10	1145.41472	573.21100	1173.40964	587.20846	E	781.34752	391.17740	6
11	1308.47804	654.74266	1336.47296	668.74012	Y	652.30492	326.65610	5
12	1422.52097	711.76412	1450.51589	725.76158	N	489.24160	245.12444	4
13	1536.56390	768.78559	1564.55882	782.78305	N	375.19867	188.10297	3
14	1650.60683	825.80705	1678.60175	839.80451	N	261.15574	131.08151	2
15					K	147.11281	74.06004	1

Fragment Spectrum

Extracted from: C:\Users\SAH\Desktop\Data\Suraya\V2085_EBA181_RH4_tryp_chymo\V2085_EBA181_P_tryp_2pmols_43C_70m09233648H4C4.raw #4970 RT: 33.82
FTMS, HCD, z=+2, Mono m/z=912.85657 Da, MH+=1824.70586 Da, Match Tol.=20 mmu

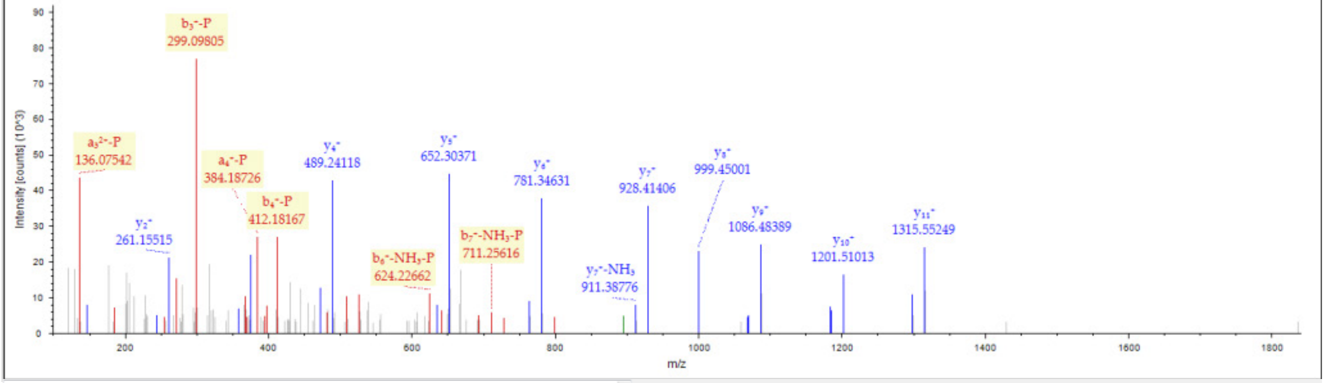

Sequence: DSNLNDSAFEYNNNK
S2-Phospho (79.96633 Da)
Charge: +2, Monoisotopic m/z: 912.85657 Da (-0.71 mmu/-0.78 ppm), MH+: 1824.70586 Da, RT: 33.82 min,

Extracted from: C:\Users\SAH\Desktop\Data\Suraya\V2085_EBA181_RH4_tryp_chymo\V2085_EBA181_P_tryp_2pmols_43C_70m09233648H4C4.raw #4970 RT: 33.82
FTMS, HCD, z=+2, Mono m/z=912.85657 Da, MH+=1824.70586 Da, Match Tol.=20 mmu

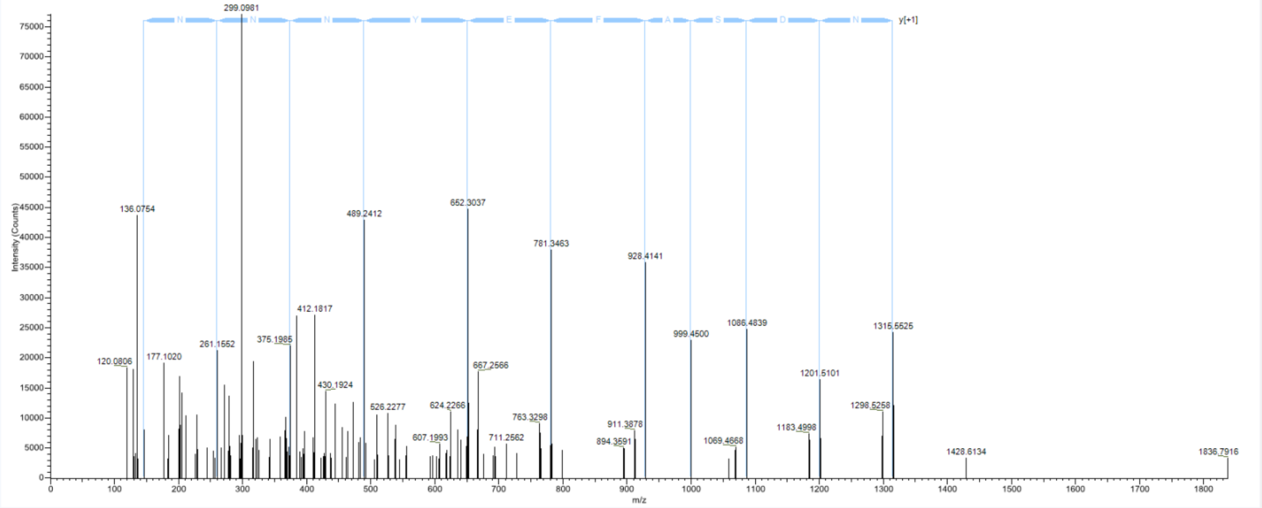

b / b* / b° / y / y* / y° / Immonium

Hide Table

	(NH3+) -	s> <N	N> <L	L> <N	N> <D	D> <S	S>	A> <F	F> <E	E> <Y	Y> <N	N> <N	N> <N	N> <K	K - (COOH)
b (+1)	116.03423	283.03259	397.07552	510.15959	624.20252	739.22947	826.26150	897.29862	1044.36704	1173.40964	1336.47296	1450.51589	1564.55882	1678.60175	1806.69672
b*(+1)	99.00768	266.00604	380.04897	493.13304	607.17597	722.20292	809.23495	880.27207	1027.34049	1156.38309	1319.44641	1433.48934	1547.53227	1661.57520	1789.67017
b°(+1)	98.02366	265.02202	379.06495	492.14902	606.19195	721.21890	808.25093	879.28805	1026.35647	1155.39907	1318.46239	1432.50532	1546.54825	1660.59118	1788.68615
y (+1)	1709.68033	1542.68197	1428.63904	1315.55497	1201.51204	1086.48509	999.45306	928.41594	781.34752	652.30492	489.24160	375.19867	261.15574	147.11281	1824.70728
y*(+1)	1692.65378	1525.65542	1411.61249	1298.52842	1184.48549	1069.45854	982.42651	911.38939	764.32097	635.27837	472.21505	358.17212	244.12919	130.08626	1807.68073
y°(+1)	1691.66977	1524.67141	1410.62848	1297.54441	1183.50148	1068.47453	981.44250	910.40538	763.33696	634.29436	471.23104	357.18811	243.14518	129.10225	1806.69672

Value Type: Theo. Mass [Da]

D s N L N D S A F E Y N N N K

G. EBA181_4

Peptide Summary

Sequence: YDSNLNDSAFEYNNNK, S3-Phospho (79.96633 Da)
Charge: +2, Monoisotopic m/z: 994.38861 Da (-0.33 mmu/-0.33 ppm), MH+: 1987.76995 Da, RT: 35.80 min,
Identified with: Mascot (v1.27); IonScore:81, Exp Value:1.2E-008, Ions matched by search engine: 11/168
Fragment match tolerance used for search: 20 mmu

Fragment Matches

Value Type: Theo. Mass [Da]

Ion Series | Phosphorylation Losses | Neutral Losses | Multiple Neutral Losses | Precursor Ions

#1	a⁺	a²⁺	b⁺	b²⁺	Seq.	y⁺	y²⁺	#2
1	136.07568	68.54148	164.07060	82.53894	Y			16
2	251.10263	126.05495	279.09755	140.05241	D	1824.70728	912.85728	15
3	418.10099	209.55413	446.09591	223.55159	S-Phospho	1709.68033	855.34380	14
4	532.14392	266.57560	560.13884	280.57306	N	1542.68197	771.84462	13
5	645.22799	323.11763	673.22291	337.11509	L	1428.63904	714.82316	12
6	759.27092	380.13910	787.26584	394.13656	N	1315.55497	658.28112	11
7	874.29787	437.65257	902.29279	451.65003	D	1201.51204	601.25966	10
8	961.32990	481.16859	989.32482	495.16605	S	1086.48509	543.74618	9
9	1032.36702	516.68715	1060.36194	530.68461	A	999.45306	500.23017	8
10	1179.43544	590.22136	1207.43036	604.21882	F	928.41594	464.71161	7
11	1308.47804	654.74266	1336.47296	668.74012	E	781.34752	391.17740	6
12	1471.54136	736.27432	1499.53628	750.27178	Y	652.30492	326.65610	5
13	1585.58429	793.29578	1613.57921	807.29324	N	489.24160	245.12444	4
14	1699.62722	850.31725	1727.62214	864.31471	N	375.19867	188.10297	3
15	1813.67015	907.33871	1841.66507	921.33617	N	261.15574	131.08151	2
16					K	147.11281	74.06004	1

Fragment Spectrum

Extracted from: C:\Users\SAH\Desktop\Data\Suraya\V2085_EBA181_RH4_tryp_chymo\V2085_EBA181_P_tryp_2pmols_43C_70m09233648H4C4.raw #5404 RT: 35.80
FTMS, HCD, z=+2, Mono m/z=994.38861 Da, MH+=1987.76995 Da, Match Tol.=20 mmu

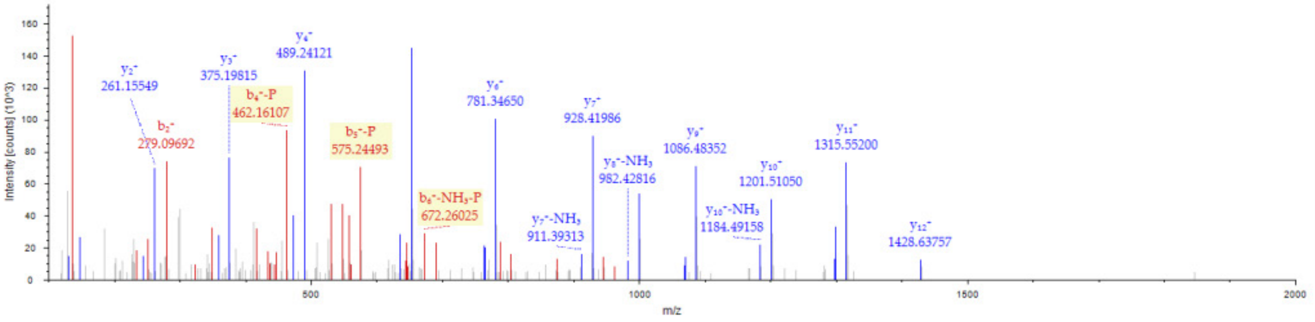

Sequence: YDSNLNDSAFEYNNNK
S3-Phospho (79.96633 Da)
Charge: +2, Monoisotopic m/z: 994.38861 Da (-0.33 mmu/-0.33 ppm), MH+: 1987.76995 Da, RT: 35.80 min,

Extracted from: C:\Users\SAH\Desktop\Data\Suraya\V2085_EBA181_RH4_tryp_chymo\V2085_EBA181_P_tryp_2pmols_43C_70m09233648H4C4.raw #5404 RT: 35.80
FTMS, HCD, z=+2, Mono m/z=994.38861 Da, MH+=1987.76995 Da, Match Tol.=20 mmu

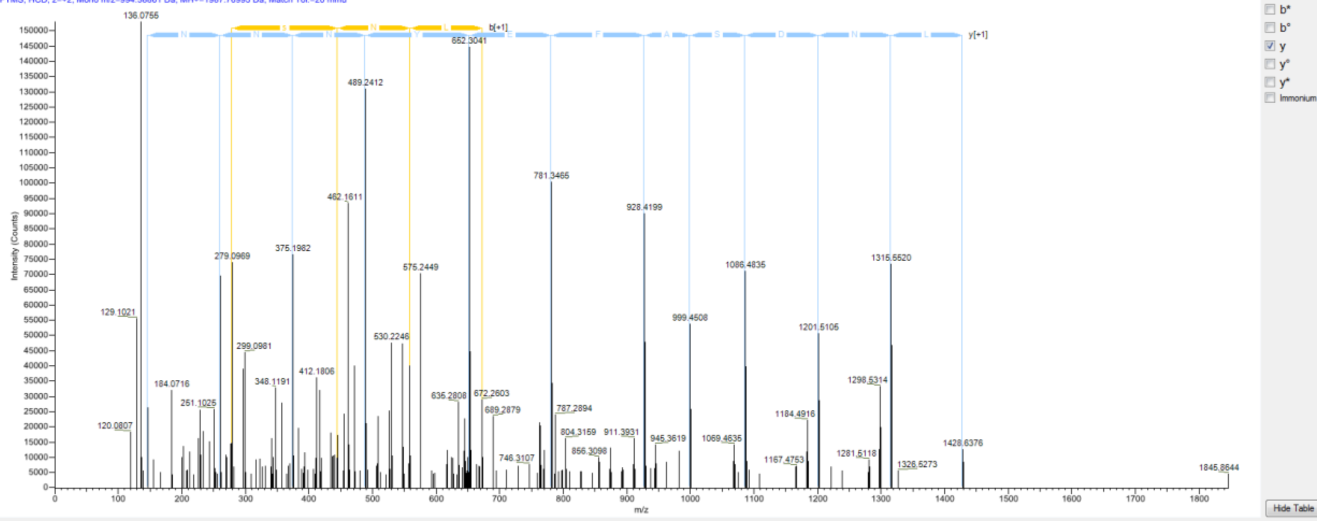

	(NH3+) -	D> <s	s> <N	N> <L	L> <N	N> <D	D> <S	S>	A> <F	F> <E	E> <Y	Y> <N	N> <N	N> <N	N> <K	K - (COOH)
b (+1)	164.07060	279.09755	446.09591	560.13884	673.22291	787.26584	902.29279	989.32482	1060.36194	1207.43036	1336.47296	1499.53628	1613.57921	1727.62214	1841.66507	1969.76004
b*(+1)	147.04405	262.07100	429.06936	543.11229	656.19636	770.23929	885.26624	972.29827	1043.33539	1190.40381	1319.44641	1482.50973	1596.55266	1710.59559	1824.63852	1952.73349
b°(+1)	146.06003	261.08698	428.08534	542.12827	655.21234	769.25527	884.28222	971.31425	1042.35137	1189.41979	1318.46239	1481.52571	1595.56864	1709.61157	1823.65450	1951.74947
y (+1)	1824.70728	1709.68033	1542.68197	1428.63904	1315.55497	1201.51204	1086.48509	999.45306	928.41594	781.34752	652.30492	489.24160	375.19867	261.15574	147.11281	1987.77060
y*(+1)	1807.68073	1692.65378	1525.65542	1411.61249	1298.52842	1184.48549	1069.45854	982.42651	911.38939	764.32097	635.27837	472.21505	358.17212	244.12919	130.08626	1970.74405
y°(+1)	1806.69672	1691.66977	1524.67141	1410.62848	1297.54441	1183.50148	1068.47453	981.44250	910.40538	763.33696	634.29436	471.23104	357.18811	243.14518	129.10225	1969.76004

Value Type: Theo. Mass [Da]

Y D⌋ s ⌋ N⌋L⌋N⌈D⌈S⌈A⌈F⌈E⌈Y⌈N⌈N⌈N⌈K

G. EBA181_5

Peptide Summary

Sequence: YDSNLNDSAFEYNNNK, S3-Phospho (79.96633 Da)
Charge: +2, Monoisotopic m/z: 994.38861 Da (-0.33 mmu/-0.33 ppm), MH+: 1987.76995 Da, RT: 35.80 min,
Identified with: Mascot (v1.27); IonScore:81, Exp Value:1.2E-008, Ions matched by search engine: 11/168
Fragment match tolerance used for search: 20 mmu

Fragment Matches

Value Type: Theo. Mass [Da]

Ion Series | Phosphorylation Losses | Neutral Losses | Multiple Neutral Losses | Precursor Ions

#1	a⁺	a²⁺	b⁺	b²⁺	Seq.	y⁺	y²⁺	#2
1	136.07568	68.54148	164.07060	82.53894	Y			16
2	251.10263	126.05495	279.09755	140.05241	D	1824.70728	912.85728	15
3	418.10099	209.55413	446.09591	223.55159	S-Phospho	1709.68033	855.34380	14
4	532.14392	266.57560	560.13884	280.57306	N	1542.68197	771.84462	13
5	645.22799	323.11763	673.22291	337.11509	L	1428.63904	714.82316	12
6	759.27092	380.13910	787.26584	394.13656	N	1315.55497	658.28112	11
7	874.29787	437.65257	902.29279	451.65003	D	1201.51204	601.25966	10
8	961.32990	481.16859	989.32482	495.16605	S	1086.48509	543.74618	9
9	1032.36702	516.68715	1060.36194	530.68461	A	999.45306	500.23017	8
10	1179.43544	590.22136	1207.43036	604.21882	F	928.41594	464.71161	7
11	1308.47804	654.74266	1336.47296	668.74012	E	781.34752	391.17740	6
12	1471.54136	736.27432	1499.53628	750.27178	Y	652.30492	326.65610	5
13	1585.58429	793.29578	1613.57921	807.29324	N	489.24160	245.12444	4
14	1699.62722	850.31725	1727.62214	864.31471	N	375.19867	188.10297	3
15	1813.67015	907.33871	1841.66507	921.33617	N	261.15574	131.08151	2
16					K	147.11281	74.06004	1

Fragment Spectrum

Extracted from: C:\Users\SAH\Desktop\Data\Suraya\V2085_EBA181_RH4_tryp_chymo\V2085_EBA181_P_tryp_2pmols_43C_70m09233648H4C4.raw #5404 RT: 35.80
FTMS, HCD, z=+2, Mono m/z=994.38861 Da, MH+=1987.76995 Da, Match Tol.=20 mmu

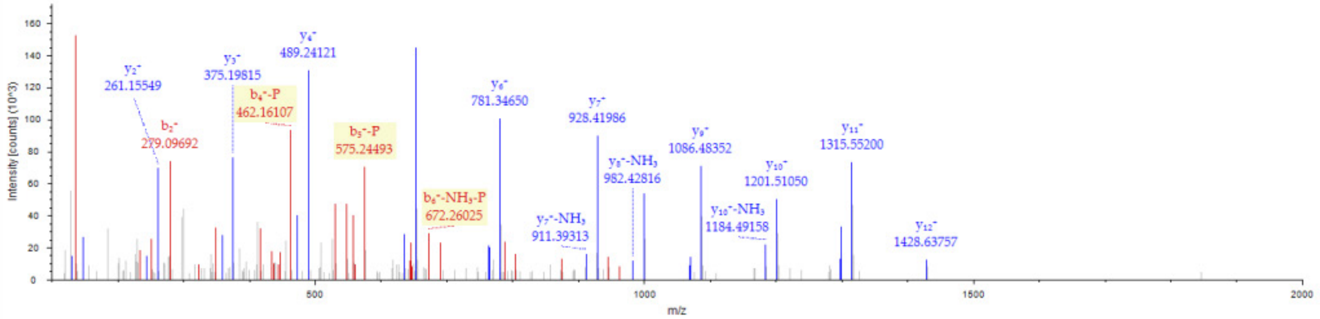

Sequence: YDSNLNDSAFEYNNNK
S3-Phospho (79.96633 Da)
Charge: +2, Monoisotopic m/z: 994.38861 Da (-0.33 mmu/-0.33 ppm), MH+: 1987.76995 Da, RT: 35.80 min,

Extracted from: C:\Users\SAH\Desktop\Data\Suraya\V2085_EBA181_RH4_tryp_chymo\V2085_EBA181_P_tryp_2pmols_43C_70m09233648H4C4.raw #5404 RT: 35.80
FTMS, HCD, z=+2, Mono m/z=994.38861 Da, MH+=1987.76995 Da, Match Tol.=20 mmu

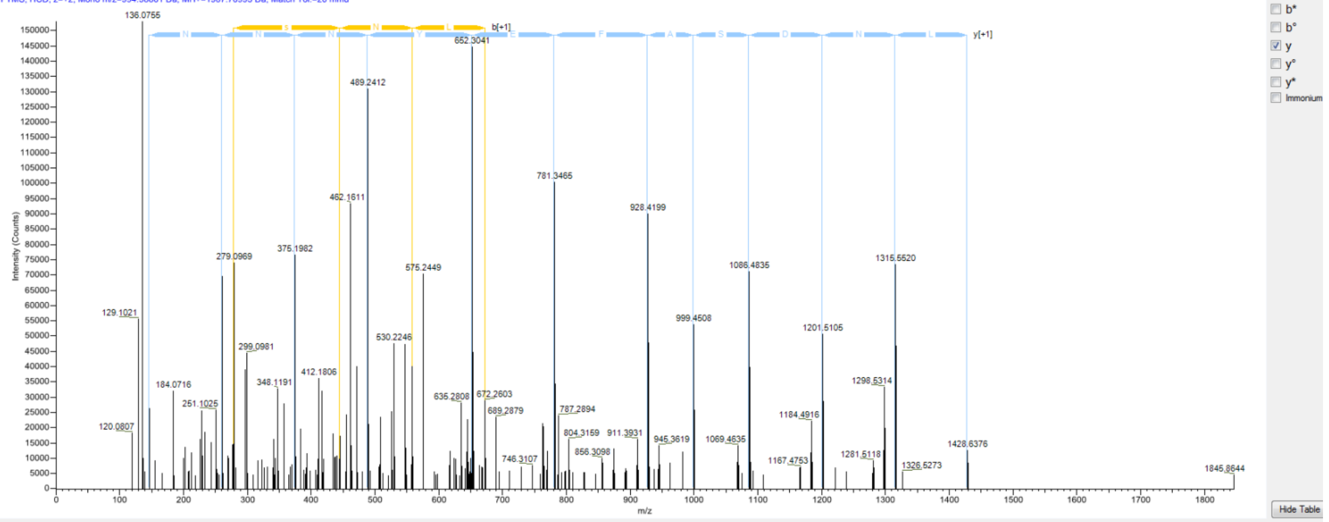

Hide Table

	(NH3+) -	D> <s	s> <N	N> <L	L> <N	N> <D	D> <S	S>	A> <F	F> <E	E> <Y	Y> <N	N> <N	N> <N	N> <K	K - (COOH)
b (+1)	164.07060	279.09755	446.09591	560.13884	673.22291	787.26584	902.29279	989.32482	1060.36194	1207.43036	1336.47296	1499.53628	1613.57921	1727.62214	1841.66507	1969.76004
b*(+1)	147.04405	262.07100	429.06936	543.11229	656.19636	770.23929	885.26624	972.29827	1043.33539	1190.40381	1319.44641	1482.50973	1596.55266	1710.59559	1824.63852	1952.73349
b°(+1)	146.06003	261.08698	428.08534	542.12827	655.21234	769.25527	884.28222	971.31425	1042.35137	1189.41979	1318.46239	1481.52571	1595.56864	1709.61157	1823.65450	1951.74947
y (+1)	1824.70728	1709.68033	1542.68197	1428.63904	1315.55497	1201.51204	1086.48509	999.45306	928.41594	781.34752	652.30492	489.24160	375.19867	261.15574	147.11281	1987.77060
y*(+1)	1807.68073	1692.65378	1525.65542	1411.61249	1298.52842	1184.48549	1069.45854	982.42651	911.38939	764.32097	635.27837	472.21505	358.17212	244.12919	130.08626	1970.74405
y°(+1)	1806.69672	1691.66977	1524.67141	1410.62848	1297.54441	1183.50148	1068.47453	981.44250	910.40538	763.33696	634.29436	471.23104	357.18811	243.14518	129.10225	1969.76004

Value Type: Theo. Mass [Da]

Y D s N L N D S A F E Y N N N K

G. EBA181_6

Peptide Summary

Sequence: SNLNDSAFEYNNNK, S1-Phospho (79.96633 Da)
Charge: +2, Monoisotopic m/z: 855.34296 Da (-0.85 mmu/-0.99 ppm), MH+: 1709.67864 Da, RT: 30.40 min,
Identified with: Mascot (v1.27); IonScore:81, Exp Value:8.4E-009, Ions matched by search engine: 8/152
Fragment match tolerance used for search: 20 mmu

Fragment Matches

Value Type: Theo. Mass [Da]

Ion Series | Phosphorylation Losses | Neutral Losses | Multiple Neutral Losses | Precursor Ions

#1	a⁺	a²⁺	b⁺	b²⁺	Seq.	y⁺	y²⁺	#2
1	140.01072	70.50900	168.00564	84.50646	S-Phospho			14
2	254.05365	127.53046	282.04857	141.52792	N	1542.68197	771.84462	13
3	367.13772	184.07250	395.13264	198.06996	L	1428.63904	714.82316	12
4	481.18065	241.09396	509.17557	255.09142	N	1315.55497	658.28112	11
5	596.20760	298.60744	624.20252	312.60490	D	1201.51204	601.25966	10
6	683.23963	342.12345	711.23455	356.12091	S	1086.48509	543.74618	9
7	754.27675	377.64201	782.27167	391.63947	A	999.45306	500.23017	8
8	901.34517	451.17622	929.34009	465.17368	F	928.41594	464.71161	7
9	1030.38777	515.69752	1058.38269	529.69498	E	781.34752	391.17740	6
10	1193.45109	597.22918	1221.44601	611.22664	Y	652.30492	326.65610	5
11	1307.49402	654.25065	1335.48894	668.24811	N	489.24160	245.12444	4
12	1421.53695	711.27211	1449.53187	725.26957	N	375.19867	188.10297	3
13	1535.57988	768.29358	1563.57480	782.29104	N	261.15574	131.08151	2
14					K	147.11281	74.06004	1

Fragment Spectrum

Extracted from: C:\Users\SAH\Desktop\Data\Suraya\V2085_EBA181_RH4_tryp_chymo\V2085_EBA181_P_tryp_2pmols_43C_70m09233648H4C4.raw #4224 RT: 30.40
FTMS, HCD, z=+2, Mono m/z=855.34296 Da, MH+=1709.67864 Da, Match Tol.=20 mmu

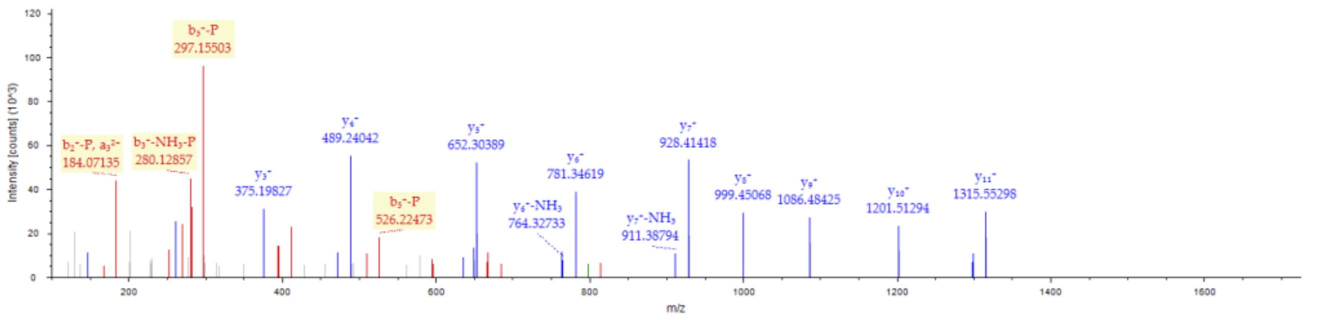

Sequence: SNLNDSAFEYNNNK
S1-Phospho (79.96633 Da)
Charge: +2, Monoisotopic m/z: 855.34296 Da (-0.85 mmu/-0.99 ppm), MH+: 1709.67864 Da, RT: 30.40 min,

Extracted from: C:\Users\SAH\Desktop\Data\Suraya\V2085_EBA181_RH4_tryp_chymo\V2085_EBA181_P_tryp_2pmols_43C_70m09233648H4C4.raw #4224 RT: 30.40
FTMS, HCD, z=+2, Mono m/z=855.34296 Da, MH+=1709.67864 Da, Match Tol.=20 mmu

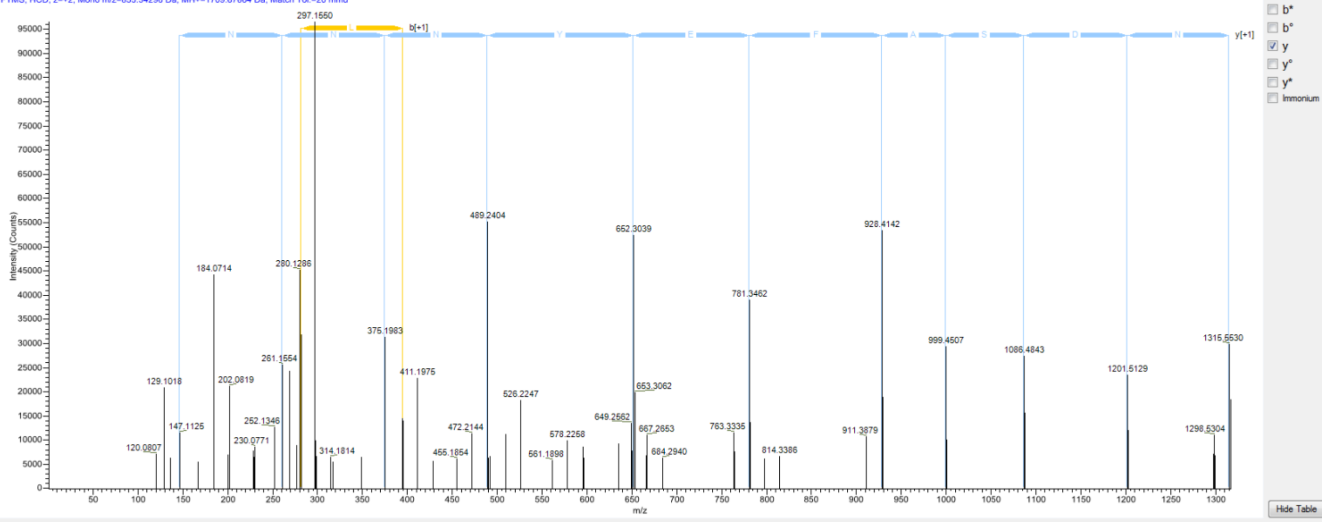

	(NH3+) - s>	N> <L	L> <N	N> <D	D> <S	S>	A> <F	F> <E	E> <Y	Y> <N	N> <N	N> <N	N> <K	K - (COOH)
b (+1)	168.00564	282.04857	395.13264	509.17557	624.20252	711.23455	782.27167	929.34009	1058.38269	1221.44601	1335.48894	1449.53187	1563.57480	1691.66977
b*(+1)	150.97909	265.02202	378.10609	492.14902	607.17597	694.20800	765.24512	912.31354	1041.35614	1204.41946	1318.46239	1432.50532	1546.54825	1674.64322
b°(+1)	149.99507	264.03800	377.12207	491.16500	606.19195	693.22398	764.26110	911.32952	1040.37212	1203.43544	1317.47837	1431.52130	1545.56423	1673.65920
y (+1)	1542.68197	1428.63904	1315.55497	1201.51204	1086.48509	999.45306	928.41594	781.34752	652.30492	489.24160	375.19867	261.15574	147.11281	1709.68033
y*(+1)	1525.65542	1411.61249	1298.52842	1184.48549	1069.45854	982.42651	911.38939	764.32097	635.27837	472.21505	358.17212	244.12919	130.08626	1692.65378
y°(+1)	1524.67141	1410.62848	1297.54441	1183.50148	1068.47453	981.44250	910.40538	763.33696	634.29436	471.23104	357.18811	243.14518	129.10225	1691.66977

Value Type: Theo. Mass [Da]

s N L N D S A F E Y N N N K

G. EBA181_7

Peptide Summary

Sequence: DSNLNDSAFEYNNNK, S2-Phospho (79.96633 Da)
Charge: +2, Monoisotopic m/z: 912.85657 Da (-0.71 mmu/-0.78 ppm), MH+: 1824.70586 Da, RT: 33.82 min,
Identified with: Mascot (v1.27); IonScore:62, Exp Value:8.9E-007, Ions matched by search engine: 9/160
Fragment match tolerance used for search: 20 mmu

Fragment Matches

Value Type: Theo. Mass [Da]

Ion Series | Phosphorylation Losses | Neutral Losses | Multiple Neutral Losses | Precursor Ions

#1	a+	a2+	b+	b2+	Seq.	y+	y2+	#2
1	88.03931	44.52329	116.03423	58.52075	D			15
2	255.03767	128.02247	283.03259	142.01993	S-Phospho	1709.68033	855.34380	14
3	369.08060	185.04394	397.07552	199.04140	N	1542.68197	771.84462	13
4	482.16467	241.58597	510.15959	255.58343	L	1428.63904	714.82316	12
5	596.20760	298.60744	624.20252	312.60490	N	1315.55497	658.28112	11
6	711.23455	356.12091	739.22947	370.11837	D	1201.51204	601.25966	10
7	798.26658	399.63693	826.26150	413.63439	S	1086.48509	543.74618	9
8	869.30370	435.15549	897.29862	449.15295	A	999.45306	500.23017	8
9	1016.37212	508.68970	1044.36704	522.68716	F	928.41594	464.71161	7
10	1145.41472	573.21100	1173.40964	587.20846	E	781.34752	391.17740	6
11	1308.47804	654.74266	1336.47296	668.74012	Y	652.30492	326.65610	5
12	1422.52097	711.76412	1450.51589	725.76158	N	489.24160	245.12444	4
13	1536.56390	768.78559	1564.55882	782.78305	N	375.19867	188.10297	3
14	1650.60683	825.80705	1678.60175	839.80451	N	261.15574	131.08151	2
15					K	147.11281	74.06004	1

Fragment Spectrum

Extracted from: C:\Users\SAH\Desktop\Data\Suraya\V2085_EBA181_RH4_tryp_chymo\V2085_EBA181_P_tryp_2pmols_43C_70m09233648H4C4.raw #4970 RT: 33.82
FTMS, HCD, z=+2, Mono m/z=912.85657 Da, MH+=1824.70586 Da, Match Tol.=20 mmu

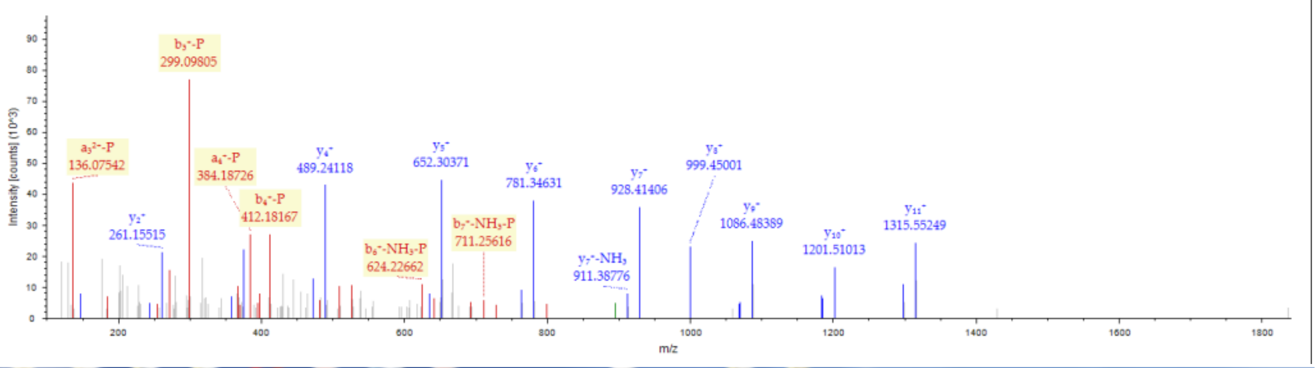

Sequence: DSNLNDSAFEYNNNK
S2-Phospho (79.96633 Da)
Charge: +2, Monoisotopic m/z: 912.85657 Da (-0.71 mmu/-0.78 ppm), MH+: 1824.70586 Da, RT: 33.82 min,

Extracted from: C:\Users\SAH\Desktop\Data\Suraya\V2085_EBA181_RH4_tryp_chymo\V2085_EBA181_P_tryp_2pmols_43C_70m09233648H4C4.raw #4970 RT: 33.82
FTMS, HCD, z=+2, Mono m/z=912.85657 Da, MH+=1824.70586 Da, Match Tol.=20 mmu

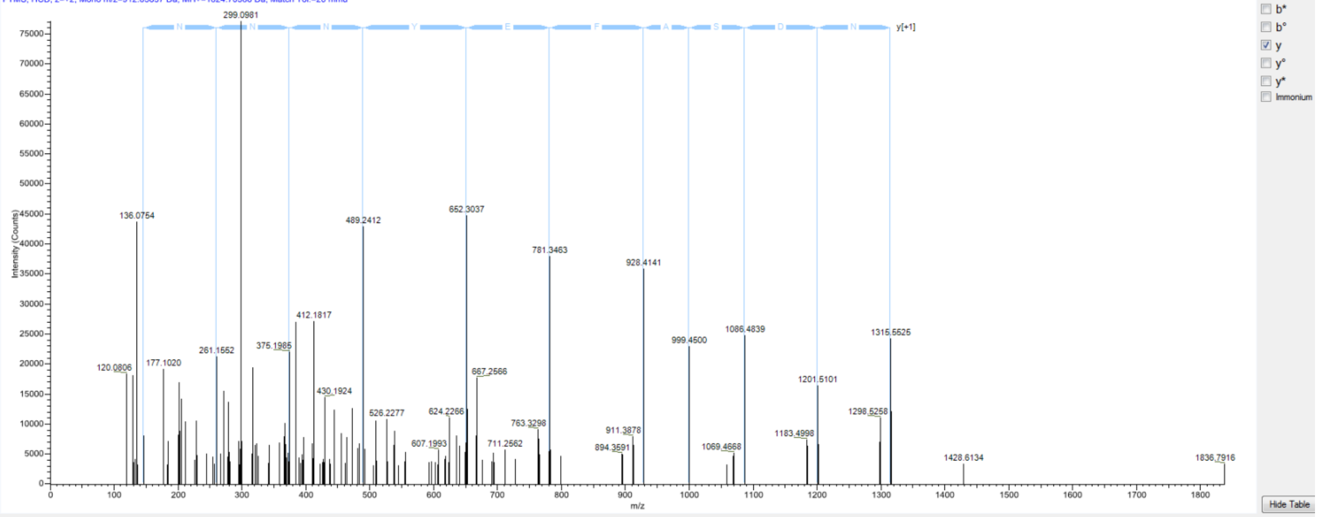

	(NH3+) -	s> <N	N> <L	L> <N	N> <D	D> <S	S>	A> <F	F> <E	E> <Y	Y> <N	N> <N	N> <N	N> <K	K - (COOH)
b (+1)	116.03423	283.03259	397.07552	510.15959	624.20252	739.22947	826.26150	897.29862	1044.36704	1173.40964	1336.47296	1450.51589	1564.55882	1678.60175	1806.69672
b*(+1)	99.00768	266.00604	380.04897	493.13304	607.17597	722.20292	809.23495	880.27207	1027.34049	1156.38309	1319.44641	1433.48934	1547.53227	1661.57520	1789.67017
b°(+1)	98.02366	265.02202	379.06495	492.14902	606.19195	721.21890	808.25093	879.28805	1026.35647	1155.39907	1318.46239	1432.50532	1546.54825	1660.59118	1788.68615
y (+1)	1709.68033	1542.68197	1428.63904	1315.55497	1201.51204	1086.48509	999.45306	928.41594	781.34752	652.30492	489.24160	375.19867	261.15574	147.11281	1824.70728
y*(+1)	1692.65378	1525.65542	1411.61249	1298.52842	1184.48549	1069.45854	982.42651	911.38939	764.32097	635.27837	472.21505	358.17212	244.12919	130.08626	1807.68073
y°(+1)	1691.66977	1524.67141	1410.62848	1297.54441	1183.50148	1068.47453	981.44250	910.40538	763.33696	634.29436	471.23104	357.18811	243.14518	129.10225	1806.69672

Value Type: Theo. Mass [Da]

D s N L N D S A F E Y N N N K

G. EBA181_8

Peptide Summary

Sequence: GFYDSNLNDSAFEYNNNKYN, S10-Phospho (79.96633 Da)
Charge: +2, Monoisotopic m/z: 1234.98474 Da (-2.27 mmu/-1.84 ppm), MH+: 2468.96221 Da, RT: 45.05 min,
Identified with: Mascot (v1.27); IonScore:60, Exp Value:1.3E-006, Ions matched by search engine: 9/208
Fragment match tolerance used for search: 20 mmu

Fragment Matches

Value Type: Theo. Mass [Da]

Ion Series | Phosphorylation Losses | Neutral Losses | Multiple Neutral Losses | Precursor Ions

#1	a⁺	a²⁺	b⁺	b²⁺	Seq.	y⁺	y²⁺	#2
1	30.03383	15.52055	58.02875	29.51801	G			20
2	177.10225	89.05476	205.09717	103.05222	F	2411.94527	1206.47627	19
3	340.16557	170.58642	368.16049	184.58388	Y	2264.87685	1132.94206	18
4	455.19252	228.09990	483.18744	242.09736	D	2101.81353	1051.41040	17
5	542.22455	271.61591	570.21947	285.61337	S	1986.78658	993.89693	16
6	656.26748	328.63738	684.26240	342.63484	N	1899.75455	950.38091	15
7	769.35155	385.17941	797.34647	399.17687	L	1785.71162	893.35945	14
8	883.39448	442.20088	911.38940	456.19834	N	1672.62755	836.81741	13
9	998.42143	499.71435	1026.41635	513.71181	D	1558.58462	779.79595	12
10	1165.41979	583.21353	1193.41471	597.21099	S-Phospho	1443.55767	722.28247	11
11	1236.45691	618.73209	1264.45183	632.72955	A	1276.55931	638.78329	10
12	1383.52533	692.26630	1411.52025	706.26376	F	1205.52219	603.26473	9
13	1512.56793	756.78760	1540.56285	770.78506	E	1058.45377	529.73052	8
14	1675.63125	838.31926	1703.62617	852.31672	Y	929.41117	465.20922	7
15	1789.67418	895.34073	1817.66910	909.33819	N	766.34785	383.67756	6
16	1903.71711	952.36219	1931.71203	966.35965	N	652.30492	326.65610	5
17	2017.76004	1009.38366	2045.75496	1023.38112	N	538.26199	269.63463	4
18	2145.85501	1073.43114	2173.84993	1087.42860	K	424.21906	212.61317	3
19	2308.91833	1154.96280	2336.91325	1168.96026	Y	296.12409	148.56568	2
20					N	133.06077	67.03402	1

Fragment Spectrum

Extracted from: C:\Users\SAH\Desktop\Data\Suraya\V2085_EBA181_RH4_tryp_chymo\V2085_EBA181_P_tryp_2pmols_43C_70m09233648H4C4.raw #7559 RT: 45.05
FTMS, HCD, z=+2, Mono m/z=1234.98474 Da, MH+=2468.96221 Da, Match Tol.=20 mmu

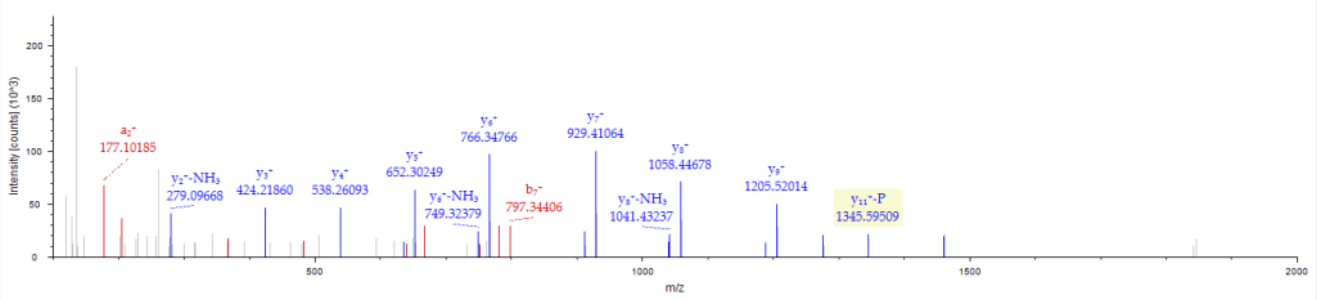

Sequence: GFYDSNLNDSAFEYNNNKYN
S10-Phospho (79.96633 Da)
Charge: +2, Monoisotopic m/z: 1234.98474 Da (-2.27 mmu/-1.84 ppm), MH+: 2468.96221 Da, RT: 45.05 min,

Extracted from: C:\Users\SAH\Desktop\Data\Suraya\V2085_EBA181_RH4_tryp_chymo\V2085_EBA181_P_tryp_2pmols_43C_70m09233648H4C4.raw #7559 RT: 45.05
FTMS, HCD, z=+2, Mono m/z=1234.98474 Da, MH+=2468.96221 Da, Match Tol.=20 mmu

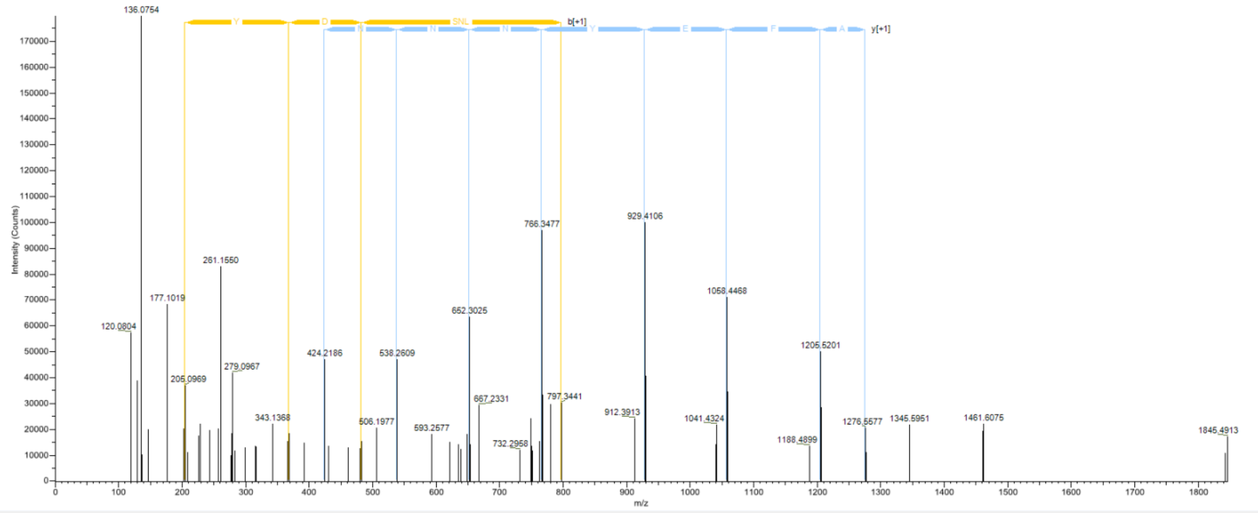

b
b*
b°
y
y°
y*
Immonium

Hide Table

	(NH3+) -	F> <Y	Y> <D	D> <S	S> <N	N> <L	L> <N	N> <D	D> <s	s> <A	A> <F	F> <E	E> <Y	Y> <N	N> <N	N> <N	N> <K	K> <Y	Y> <N	N - (COOH)
b (+1)	58.02875	205.09717	368.16049	483.18744	570.21947	684.26240	797.34647	911.38940	1026.41635	1193.41471	1264.45183	1411.52025	1540.56285	1703.62617	1817.66910	1931.71203	2045.75496	2173.84993	2336.91325	2450.95618
b*(+1)	41.00220	188.07062	351.13394	466.16089	553.19292	667.23585	780.31992	894.36285	1009.38980	1176.38816	1247.42528	1394.49370	1523.53630	1686.59962	1800.64255	1914.68548	2028.72841	2156.82338	2319.88670	2433.92963
b°(+1)	40.01818	187.08660	350.14992	465.17687	552.20890	666.25183	779.33590	893.37883	1008.40578	1175.40414	1246.44126	1393.50968	1522.55228	1685.61560	1799.65853	1913.70146	2027.74439	2155.83936	2318.90268	2432.94561
y (+1)	2411.94527	2264.87685	2101.81353	1986.78658	1899.75455	1785.71162	1672.62755	1558.58462	1443.55767	1276.55931	1205.52219	1058.45377	929.41117	766.34785	652.30492	538.26199	424.21906	296.12409	133.06077	2468.96674
y*(+1)	2394.91872	2247.85030	2084.78698	1969.76003	1882.72800	1768.68507	1655.60100	1541.55807	1426.53112	1259.53276	1188.49564	1041.42722	912.38462	749.32130	635.27837	521.23544	407.19251	279.09754	116.03422	2451.94019
y°(+1)	2393.93471	2246.86629	2083.80297	1968.77602	1881.74399	1767.70106	1654.61699	1540.57406	1425.54711	1258.54875	1187.51163	1040.44321	911.40061	748.33729	634.29436	520.25143	406.20850	278.11353	115.05021	2450.95618

Value Type:
Theo. Mass [Da]

G F⌋Y⌋D⌋S N L⌋N D **s**⌈A⌈F⌈E⌈Y⌈N⌈N⌈N⌈K Y N

G. EBA181_9

Peptide Summary

Sequence: GFYDSNLNDSAFEYNNNKY, S10-Phospho (79.96633 Da)
Charge: +2, Monoisotopic m/z: 1177.96436 Da (-1.19 mmu/-1.01 ppm), MH+: 2354.92143 Da, RT: 46.58 min,
Identified with: Mascot (v1.27); IonScore:36, Exp Value:3.3E-004, Ions matched by search engine: 6/194
Fragment match tolerance used for search: 20 mmu

Fragment Matches

Value Type: Theo. Mass [Da]

Ion Series | Phosphorylation Losses | Neutral Losses | Multiple Neutral Losses | Precursor Ions

#1	a⁺	a²⁺	b⁺	b²⁺	Seq.	y⁺	y²⁺	#2
1	30.03383	15.52055	58.02875	29.51801	G			19
2	177.10225	89.05476	205.09717	103.05222	F	2297.90234	1149.45481	18
3	340.16557	170.58642	368.16049	184.58388	Y	2150.83392	1075.92060	17
4	455.19252	228.09990	483.18744	242.09736	D	1987.77060	994.38894	16
5	542.22455	271.61591	570.21947	285.61337	S	1872.74365	936.87546	15
6	656.26748	328.63738	684.26240	342.63484	N	1785.71162	893.35945	14
7	769.35155	385.17941	797.34647	399.17687	L	1671.66869	836.33798	13
8	883.39448	442.20088	911.38940	456.19834	N	1558.58462	779.79595	12
9	998.42143	499.71435	1026.41635	513.71181	D	1444.54169	722.77448	11
10	1165.41979	583.21353	1193.41471	597.21099	S-Phospho	1329.51474	665.26101	10
11	1236.45691	618.73209	1264.45183	632.72955	A	1162.51638	581.76183	9
12	1383.52533	692.26630	1411.52025	706.26376	F	1091.47926	546.24327	8
13	1512.56793	756.78760	1540.56285	770.78506	E	944.41084	472.70906	7
14	1675.63125	838.31926	1703.62617	852.31672	Y	815.36824	408.18776	6
15	1789.67418	895.34073	1817.66910	909.33819	N	652.30492	326.65610	5
16	1903.71711	952.36219	1931.71203	966.35965	N	538.26199	269.63463	4
17	2017.76004	1009.38366	2045.75496	1023.38112	N	424.21906	212.61317	3
18	2145.85501	1073.43114	2173.84993	1087.42860	K	310.17613	155.59170	2
19					Y	182.08116	91.54422	1

Fragment Spectrum

Extracted from: C:\Users\SAH\Desktop\Data\Suraya\V2085_EBA181_RH4_tryp_chymo\V2085_EBA181_P_tryp_2pmols_43C_70m09233648H4C4.raw #7918 RT: 46.58
FTMS, HCD, z=+2, Mono m/z=1177.96436 Da, MH+=2354.92143 Da, Match Tol.=20 mmu

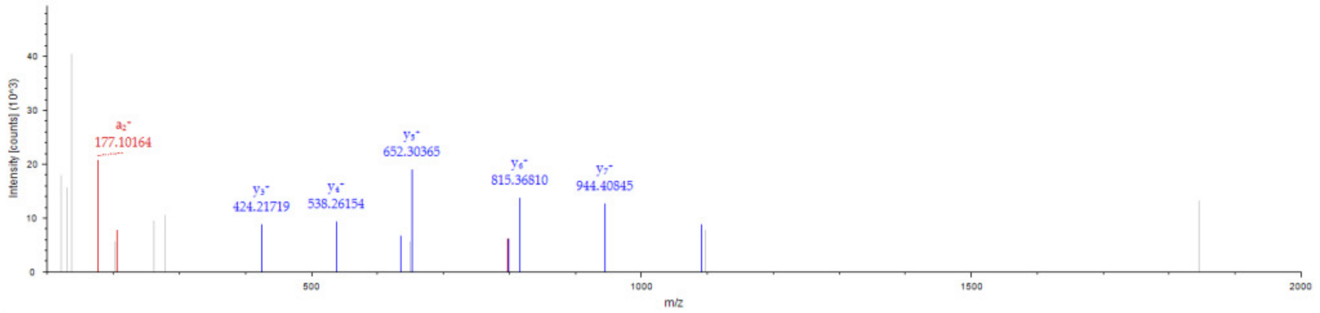

Sequence: GFYDSNLNDSAFEYNNNKY
S10-Phospho (79.96633 Da)
Charge: +2, Monoisotopic m/z: 1177.96436 Da (-1.19 mmu/-1.01 ppm), MH+: 2354.92143 Da, RT: 46.58 min,

Extracted from: C:\Users\SAH\Desktop\Data\Suraya\V2085_EBA181_RH4_tryp_chymo\V2085_EBA181_P_tryp_2pmols_43C_70m09233648H4C4.raw #7918 RT: 46.58
FTMS, HCD, z=+2, Mono m/z=1177.96436 Da, MH+=2354.92143 Da, Match Tol.=20 mmu

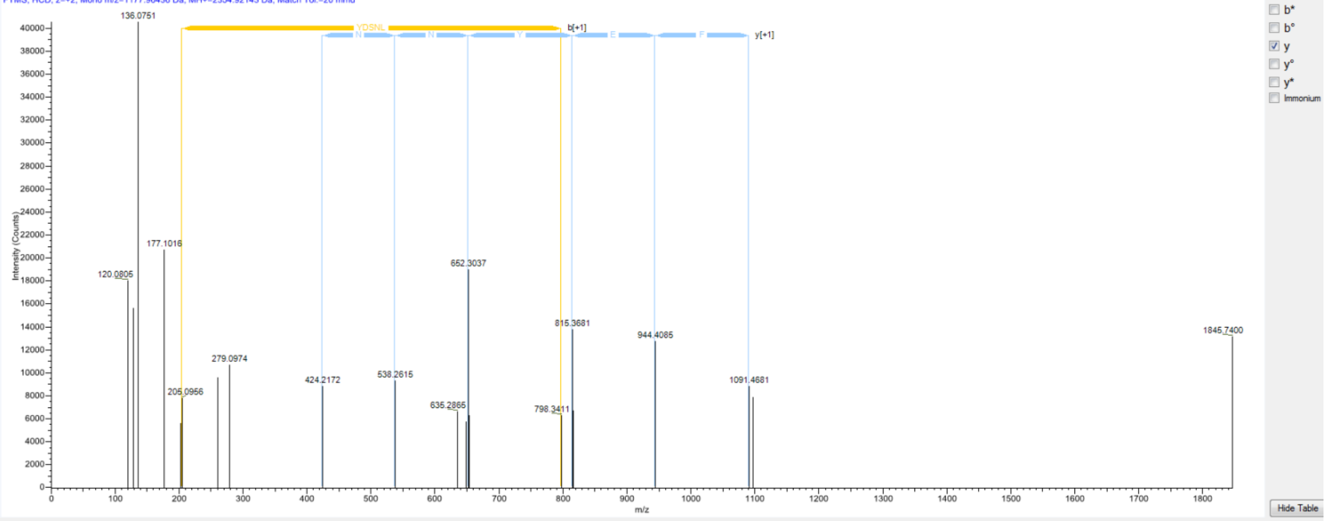

b | b* | b° | y | y* | y° | Immonium

Hide Table

	(NH3+) -	F> <Y	Y> <D	D> <S	S> <N	N> <L	L> <N	N> <D	D> <s	s> <A	A> <F	F> <E	E> <Y	Y> <N	N> <N	N> <N	N> <K	K> <Y	Y - (COOH)
b (+1)	58.02875	205.09717	368.16049	483.18744	570.21947	684.26240	797.34647	911.38940	1026.41635	1193.41471	1264.45183	1411.52025	1540.56285	1703.62617	1817.66910	1931.71203	2045.75496	2173.84993	2336.91325
b*(+1)	41.00220	188.07062	351.13394	466.16089	553.19292	667.23585	780.31992	894.36285	1009.38980	1176.38816	1247.42528	1394.49370	1523.53630	1686.59962	1800.64255	1914.68548	2028.72841	2156.82338	2319.88670
b°(+1)	40.01818	187.08660	350.14992	465.17687	552.20890	666.25183	779.33590	893.37883	1008.40578	1175.40414	1246.44126	1393.50968	1522.55228	1685.61560	1799.65853	1913.70146	2027.74439	2155.83936	2318.90268
y (+1)	2297.90234	2150.83392	1987.77060	1872.74365	1785.71162	1671.66869	1558.58462	1444.54169	1329.51474	1162.51638	1091.47926	944.41084	815.36824	652.30492	538.26199	424.21906	310.17613	182.08116	2354.92381
y*(+1)	2280.87579	2133.80737	1970.74405	1855.71710	1768.68507	1654.64214	1541.55807	1427.51514	1312.48819	1145.48983	1074.45271	927.38429	798.34169	635.27837	521.23544	407.19251	293.14958	165.05461	2337.89726
y°(+1)	2279.89178	2132.82336	1969.76004	1854.73309	1767.70106	1653.65813	1540.57406	1426.53113	1311.50418	1144.50582	1073.46870	926.40028	797.35768	634.29436	520.25143	406.20850	292.16557	164.07060	2336.91325

Value Type: Theo. Mass [Da]

G F⌋Y D S N L⌋N D *s* A⌈F⌈E⌈Y⌈N⌈N⌈N K Y

G. EBA181_10

Peptide Summary

Sequence: GFYDSNLNDSAFEYNNNK, S10-Phospho (79.96633 Da)
Charge: +2, Monoisotopic m/z: 1096.43408 Da (+0.2 mmu/+0.18 ppm), MH+: 2191.86089 Da, RT: 44.12 min,
Identified with: Mascot (v1.27); IonScore:83, Exp Value:1.2E-008, Ions matched by search engine: 10/184
Fragment match tolerance used for search: 20 mmu

Fragment Matches

Value Type: Theo. Mass [Da]

Ion Series | Phosphorylation Losses | Neutral Losses | Multiple Neutral Losses | Precursor Ions

#1	a^+	a^{2+}	b^+	b^{2+}	Seq.	y^+	y^{2+}	#2
1	30.03383	15.52055	58.02875	29.51801	G			18
2	177.10225	89.05476	205.09717	103.05222	F	2134.83902	1067.92315	17
3	340.16557	170.58642	368.16049	184.58388	Y	1987.77060	994.38894	16
4	455.19252	228.09990	483.18744	242.09736	D	1824.70728	912.85728	15
5	542.22455	271.61591	570.21947	285.61337	S	1709.68033	855.34380	14
6	656.26748	328.63738	684.26240	342.63484	N	1622.64830	811.82779	13
7	769.35155	385.17941	797.34647	399.17687	L	1508.60537	754.80632	12
8	883.39448	442.20088	911.38940	456.19834	N	1395.52130	698.26429	11
9	998.42143	499.71435	1026.41635	513.71181	D	1281.47837	641.24282	10
10	1165.41979	583.21353	1193.41471	597.21099	S-Phospho	1166.45142	583.72935	9
11	1236.45691	618.73209	1264.45183	632.72955	A	999.45306	500.23017	8
12	1383.52533	692.26630	1411.52025	706.26376	F	928.41594	464.71161	7
13	1512.56793	756.78760	1540.56285	770.78506	E	781.34752	391.17740	6
14	1675.63125	838.31926	1703.62617	852.31672	Y	652.30492	326.65610	5
15	1789.67418	895.34073	1817.66910	909.33819	N	489.24160	245.12444	4
16	1903.71711	952.36219	1931.71203	966.35965	N	375.19867	188.10297	3
17	2017.76004	1009.38366	2045.75496	1023.38112	N	261.15574	131.08151	2
18					K	147.11281	74.06004	1

Fragment Spectrum

Extracted from: C:\Users\SAH\Desktop\Data\Suraya\V2085_EBA181_RH4_tryp_chymo\V2085_EBA181_P_tryp_2pmols_43C_70m09233648H4C4.raw #7340 RT: 44.12
FTMS, HCD, z=+2, Mono m/z=1096.43408 Da, MH+=2191.86089 Da, Match Tol.=20 mmu

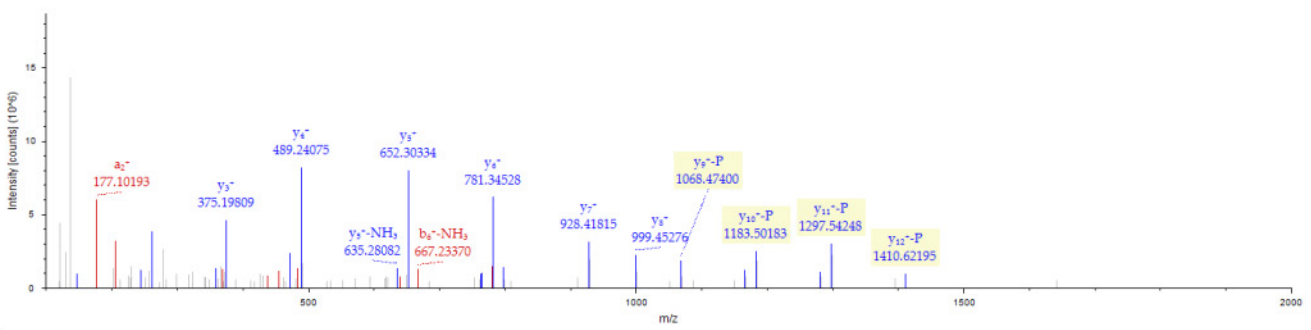

Sequence: GFYDSNLNDSAFEYNNNK
S10-Phospho (79.96633 Da)
Charge: +2, Monoisotopic m/z: 1096.43408 Da (+0.2 mmu/+0.18 ppm), MH+: 2191.86089 Da, RT: 44.12 min,

Extracted from: C:\Users\SAH\Desktop\Data\Suraya\V2085_EBA181_RH4_tryp_chymo\V2085_EBA181_P_tryp_2pmols_43C_70m09233648H4C4.raw #7340 RT: 44.12
FTMS, HCD, z=+2, Mono m/z=1096.43408 Da, MH+=2191.86089 Da, Match Tol.=20 mmu

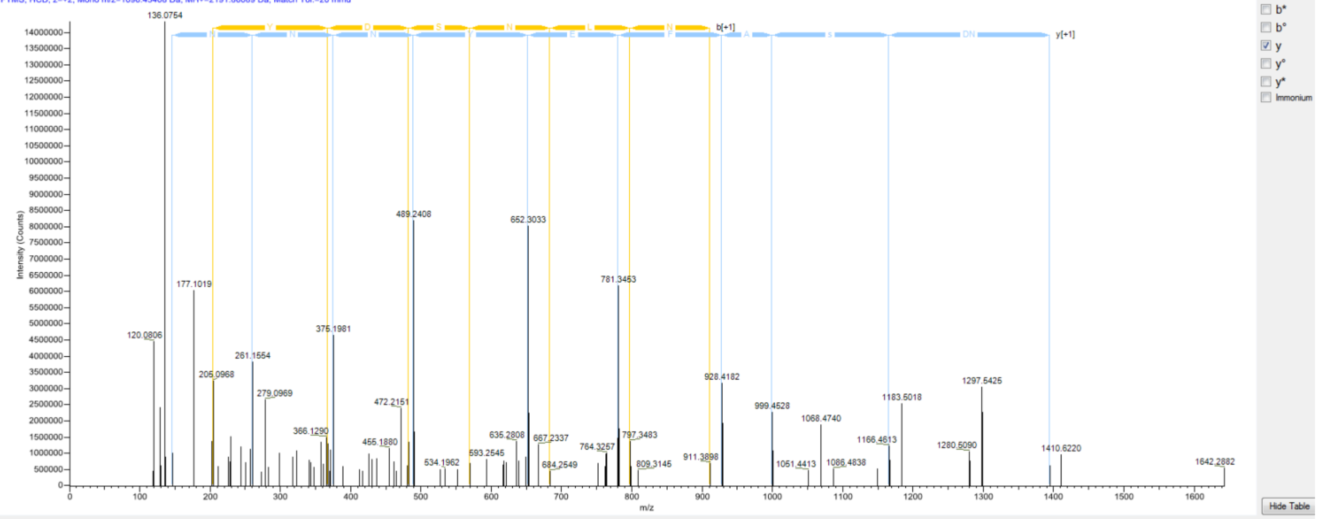

	(NH3+) -	F> <Y	Y> <D	D> <S	S> <N	N> <L	L> <N	N> <D	D> <s	s> <A	A> <F	F> <E	E> <Y	Y> <N	N> <N	N> <N	N> <K	K - (COOH)
b (+1)	58.02875	205.09717	368.16049	483.18744	570.21947	684.26240	797.34647	911.38940	1026.41635	1193.41471	1264.45183	1411.52025	1540.56285	1703.62617	1817.66910	1931.71203	2045.75496	2173.84993
b*(+1)	41.00220	188.07062	351.13394	466.16089	553.19292	667.23585	780.31992	894.36285	1009.38980	1176.38816	1247.42528	1394.49370	1523.53630	1686.59962	1800.64255	1914.68548	2028.72841	2156.82338
b°(+1)	40.01818	187.08660	350.14992	465.17687	552.20890	666.25183	779.33590	893.37883	1008.40578	1175.40414	1246.44126	1393.50968	1522.55228	1685.61560	1799.65853	1913.70146	2027.74439	2155.83936
y (+1)	2134.83902	1987.77060	1824.70728	1709.68033	1622.64830	1508.60537	1395.52130	1281.47837	1166.45142	999.45306	928.41594	781.34752	652.30492	489.24160	375.19867	261.15574	147.11281	2191.86049
y*(+1)	2117.81247	1970.74405	1807.68073	1692.65378	1605.62175	1491.57882	1378.49475	1264.45182	1149.42487	982.42651	911.38939	764.32097	635.27837	472.21505	358.17212	244.12919	130.08626	2174.83394
y°(+1)	2116.82846	1969.76004	1806.69672	1691.66977	1604.63774	1490.59481	1377.51074	1263.46781	1148.44086	981.44250	910.40538	763.33696	634.29436	471.23104	357.18811	243.14518	129.10225	2173.84993

Value Type: Theo. Mass [Da]

G F⌋ Y⌋ D⌋ S⌋ N⌋ L⌈N⌋ D⌈**s** ⌈A ⌈F ⌈E ⌈Y ⌈N ⌈N ⌈N ⌈K

G. EBA181_11

Peptide Summary

Sequence: FYDSNLNDSAFEYNNNK, S9-Phospho (79.96633 Da)
Charge: +2, Monoisotopic m/z: 1067.92114 Da (-2.01 mmu/-1.88 ppm), MH+: 2134.83501 Da, RT: 41.02 min,
Identified with: Mascot (v1.27); IonScore:80, Exp Value:2.2E-008, Ions matched by search engine: 11/176
Fragment match tolerance used for search: 20 mmu

Fragment Matches

Value Type: Theo. Mass [Da]

Ion Series | Phosphorylation Losses | Neutral Losses | Multiple Neutral Losses | Precursor Ions

#1	a+	a2+	b+	b2+	Seq.	y+	y2+	#2
1	120.08078	60.54403	148.07570	74.54149	F			17
2	283.14410	142.07569	311.13902	156.07315	Y	1987.77060	994.38894	16
3	398.17105	199.58916	426.16597	213.58662	D	1824.70728	912.85728	15
4	485.20308	243.10518	513.19800	257.10264	S	1709.68033	855.34380	14
5	599.24601	300.12664	627.24093	314.12410	N	1622.64830	811.82779	13
6	712.33008	356.66868	740.32500	370.66614	L	1508.60537	754.80632	12
7	826.37301	413.69014	854.36793	427.68760	N	1395.52130	698.26429	11
8	941.39996	471.20362	969.39488	485.20108	D	1281.47837	641.24282	10
9	1108.39832	554.70280	1136.39324	568.70026	S-Phospho	1166.45142	583.72935	9
10	1179.43544	590.22136	1207.43036	604.21882	A	999.45306	500.23017	8
11	1326.50386	663.75557	1354.49878	677.75303	F	928.41594	464.71161	7
12	1455.54646	728.27687	1483.54138	742.27433	E	781.34752	391.17740	6
13	1618.60978	809.80853	1646.60470	823.80599	Y	652.30492	326.65610	5
14	1732.65271	866.82999	1760.64763	880.82745	N	489.24160	245.12444	4
15	1846.69564	923.85146	1874.69056	937.84892	N	375.19867	188.10297	3
16	1960.73857	980.87292	1988.73349	994.87038	N	261.15574	131.08151	2
17					K	147.11281	74.06004	1

Fragment Spectrum

Extracted from: C:\Users\SAH\Desktop\Data\Suraya\V2085_EBA181_RH4_tryp_chymo\V2085_EBA181_P_tryp_2pmols_43C_70m09233648H4C4.raw #6611 RT: 41.02
FTMS, HCD, z=+2, Mono m/z=1067.92114 Da, MH+=2134.83501 Da, Match Tol.=20 mmu

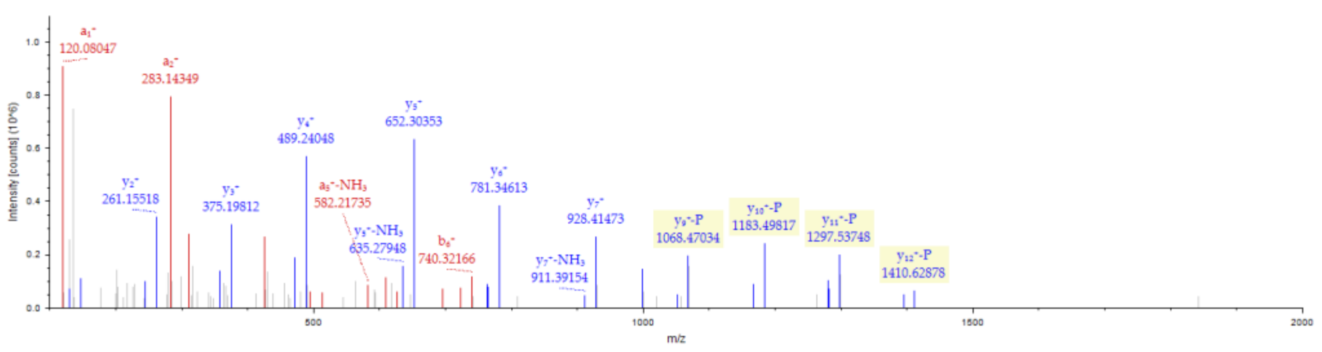

Sequence: FYDSNLNDSAFEYNNNK
S9-Phospho (79.96633 Da)
Charge: +2, Monoisotopic m/z: 1067.92114 Da (-2.01 mmu/-1.88 ppm), MH+: 2134.83501 Da, RT: 41.02 min,

Extracted from: C:\Users\SAH\Desktop\Data\Suraya\V2085_EBA181_RH4_tryp_chymo\V2085_EBA181_P_tryp_2pmols_43C_70m09233648H4C4.raw #6611 RT: 41.02
FTMS, HCD, z=+2, Mono m/z=1067.92114 Da, MH+=2134.83501 Da, Match Tol.=20 mmu

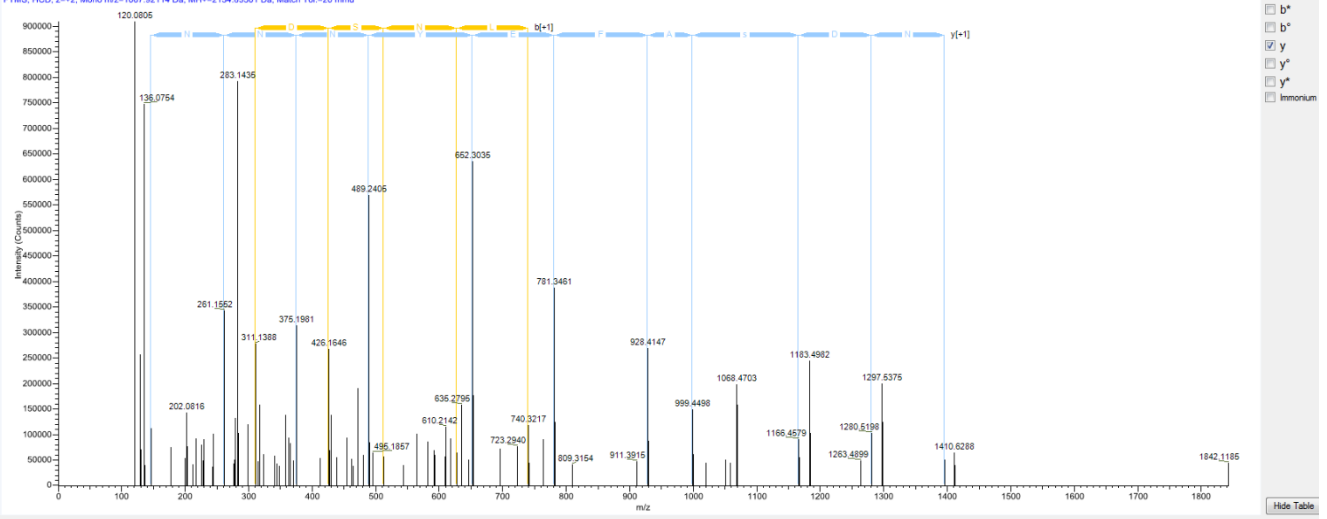

Hide Table

	(NH3+) - F>	Y> <D	D> <S	S> <N	N> <L	L> <N	N> <D	D> <s	s> <A	A> <F	F> <E	E> <Y	Y> <N	N> <N	N> <N	N> <K	K - (COOH)
b (+1)	148.07570	311.13902	426.16597	513.19800	627.24093	740.32500	854.36793	969.39488	1136.39324	1207.43036	1354.49878	1483.54138	1646.60470	1760.64763	1874.69056	1988.73349	2116.82846
b*(+1)	131.04915	294.11247	409.13942	496.17145	610.21438	723.29845	837.34138	952.36833	1119.36669	1190.40381	1337.47223	1466.51483	1629.57815	1743.62108	1857.66401	1971.70694	2099.80191
b°(+1)	130.06513	293.12845	408.15540	495.18743	609.23036	722.31443	836.35736	951.38431	1118.38267	1189.41979	1336.48821	1465.53081	1628.59413	1742.63706	1856.67999	1970.72292	2098.81789
y (+1)	1987.77060	1824.70728	1709.68033	1622.64830	1508.60537	1395.52130	1281.47837	1166.45142	999.45306	928.41594	781.34752	652.30492	489.24160	375.19867	261.15574	147.11281	2134.83902
y*(+1)	1970.74405	1807.68073	1692.65378	1605.62175	1491.57882	1378.49475	1264.45182	1149.42487	982.42651	911.38939	764.32097	635.27837	472.21505	358.17212	244.12919	130.08626	2117.81247
y°(+1)	1969.76004	1806.69672	1691.66977	1604.63774	1490.59481	1377.51074	1263.46781	1148.44086	981.44250	910.40538	763.33696	634.29436	471.23104	357.18811	243.14518	129.10225	2116.82846

Value Type: Theo. Mass [Da]

F Y⌋ D⌋ S⌋ N⌋ L⌋⌈N⌈D⌈**s**⌈A⌈F⌈E⌈Y⌈N⌈N⌈N⌈K

G. EBA181_12

Peptide Summary

Sequence: LNDSAFEYNNNK, S4-Phospho (79.96633 Da)
Charge: +2, Monoisotopic m/z: 754.80463 Da (-1.7 mmu/-2.25 ppm), MH+: 1508.60198 Da, RT: 29.08 min,
Identified with: Mascot (v1.27); IonScore:86, Exp Value:2.7E-009, Ions matched by search engine: 11/128
Fragment match tolerance used for search: 20 mmu

Fragment Matches

Value Type: Theo. Mass [Da]

Ion Series | Phosphorylation Losses | Neutral Losses | Multiple Neutral Losses | Precursor Ions

#1	a⁺	a²⁺	b⁺	b²⁺	Seq.	y⁺	y²⁺	#2
1	86.09643	43.55185	114.09135	57.54931	L			12
2	200.13936	100.57332	228.13428	114.57078	N	1395.52130	698.26429	11
3	315.16631	158.08679	343.16123	172.08425	D	1281.47837	641.24282	10
4	482.16467	241.58597	510.15959	255.58343	S-Phospho	1166.45142	583.72935	9
5	553.20179	277.10453	581.19671	291.10199	A	999.45306	500.23017	8
6	700.27021	350.63874	728.26513	364.63620	F	928.41594	464.71161	7
7	829.31281	415.16004	857.30773	429.15750	E	781.34752	391.17740	6
8	992.37613	496.69170	1020.37105	510.68916	Y	652.30492	326.65610	5
9	1106.41906	553.71317	1134.41398	567.71063	N	489.24160	245.12444	4
10	1220.46199	610.73463	1248.45691	624.73209	N	375.19867	188.10297	3
11	1334.50492	667.75610	1362.49984	681.75356	N	261.15574	131.08151	2
12					K	147.11281	74.06004	1

Fragment Spectrum

Extracted from: C:\Users\SAH\Desktop\Data\Suraya\V2085_EBA181_RH4_tryp_chymo\V2085_EBA181_P_tryp_2pmols_43C_70m09233648H4C4.raw #3926 RT: 29.08
FTMS, HCD, z=+2, Mono m/z=754.80463 Da, MH+=1508.60198 Da, Match Tol.=20 mmu

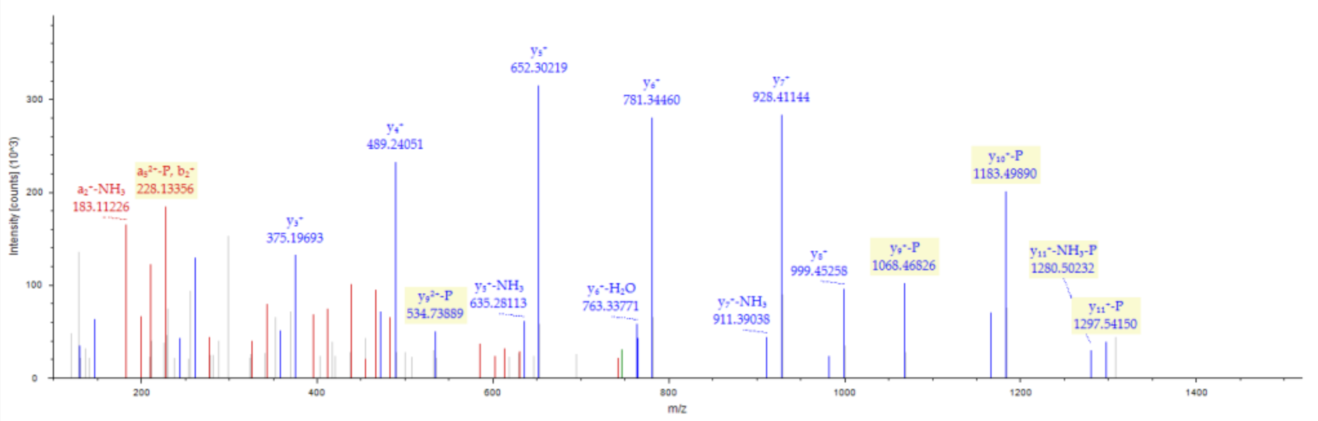

Sequence: LNDSAFEYNNNK
S4-Phospho (79.96633 Da)
Charge: +2, Monoisotopic m/z: 754.80463 Da (-1.7 mmu/-2.25 ppm), MH+: 1508.60198 Da, RT: 29.08 min,

Extracted from: C:\Users\SAH\Desktop\Data\Suraya\V2085_EBA181_RH4_tryp_chymo\V2085_EBA181_P_tryp_2pmols_43C_70m09233648H4C4.raw #3926 RT: 29.08
FTMS, HCD, z=+2, Mono m/z=754.80463 Da, MH+=1508.60198 Da, Match Tol.=20 mmu

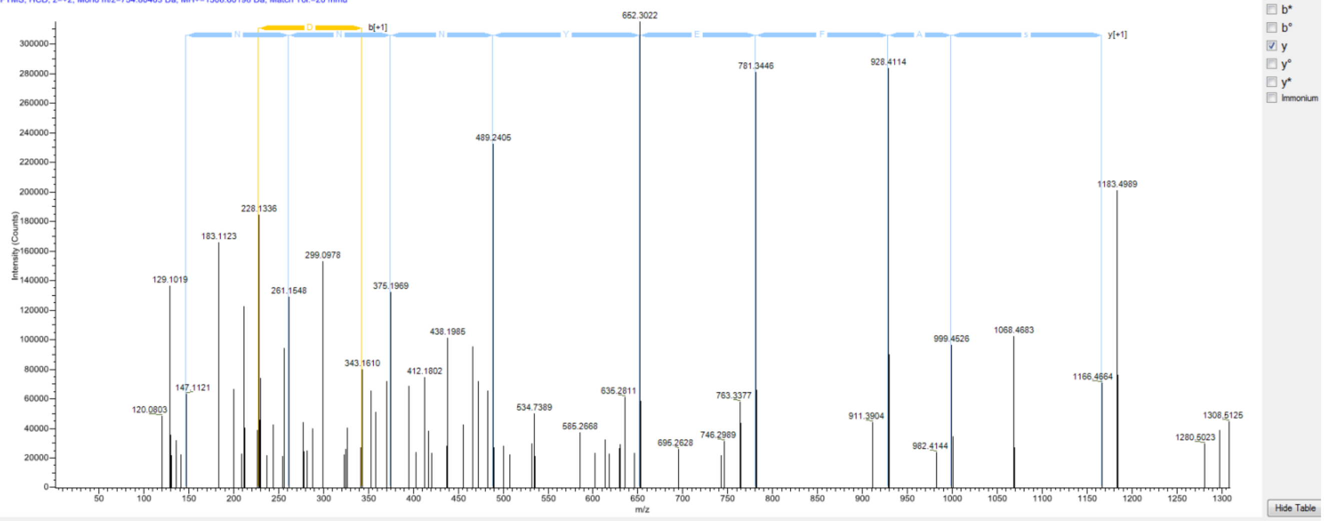

	(NH3+) - L>	N> <D	D> <s	s> <A	A> <F	F> <E	E>	Y> <N	N> <N	N> <N	N> <K	K - (COOH)
b (+1)	114.09135	228.13428	343.16123	510.15959	581.19671	728.26513	857.30773	1020.37105	1134.41398	1248.45691	1362.49984	1490.59481
b*(+1)	97.06480	211.10773	326.13468	493.13304	564.17016	711.23858	840.28118	1003.34450	1117.38743	1231.43036	1345.47329	1473.56826
b°(+1)	96.08078	210.12371	325.15066	492.14902	563.18614	710.25456	839.29716	1002.36048	1116.40341	1230.44634	1344.48927	1472.58424
y (+1)	1395.52130	1281.47837	1166.45142	999.45306	928.41594	781.34752	652.30492	489.24160	375.19867	261.15574	147.11281	1508.60537
y*(+1)	1378.49475	1264.45182	1149.42487	982.42651	911.38939	764.32097	635.27837	472.21505	358.17212	244.12919	130.08626	1491.57882
y°(+1)	1377.51074	1263.46781	1148.44086	981.44250	910.40538	763.33696	634.29436	471.23104	357.18811	243.14518	129.10225	1490.59481

Value Type: Theo. Mass [Da]

L N⌋ D⌋ s ⌈A ⌈F ⌈E ⌈Y ⌈N ⌈N ⌈N ⌈K

G. EBA181_13

Peptide Summary

Sequence: GFYDSNLNDSAFEYNNNK, S10-Phospho (79.96633 Da)
Charge: +2, Monoisotopic m/z: 1096.43347 Da (-0.41 mmu/-0.38 ppm), MH+: 2191.85967 Da, RT: 43.25 min,
Identified with: Mascot (v1.27); IonScore:58, Exp Value:1.9E-005, Ions matched by search engine: 11/184
Fragment match tolerance used for search: 20 mmu

Fragment Matches

Value Type: Theo. Mass [Da]

Ion Series | Phosphorylation Losses | Neutral Losses | Multiple Neutral Losses | Precursor Ions

#1	a+	a2+	b+	b2+	Seq.	y+	y2+	#2
1	30.03383	15.52055	58.02875	29.51801	G			18
2	177.10225	89.05476	205.09717	103.05222	F	2134.83902	1067.92315	17
3	340.16557	170.58642	368.16049	184.58388	Y	1987.77060	994.38894	16
4	455.19252	228.09990 -0.15 mmu/-0.42 ppm	[illegible]	242.09736	D	1824.70728	912.85728	15
5	542.22455	271.61591	570.21947	285.61337	S	1709.68033	855.34380	14
6	656.26748	328.63738	684.26240	342.63484	N	1622.64830	811.82779	13
7	769.35155	385.17941	797.34647	399.17687	L	1508.60537	754.80632	12
8	883.39448	442.20088	911.38940	456.19834	N	1395.52130	698.26429	11
9	998.42143	499.71435	1026.41635	513.71181	D	1281.47837	641.24282	10
10	1165.41979	583.21353	1193.41471	597.21099	S-Phospho	1166.45142	583.72935	9
11	1236.45691	618.73209	1264.45183	632.72955	A	999.45306	500.23017	8
12	1383.52533	692.26630	1411.52025	706.26376	F	928.41594	464.71161	7
13	1512.56793	756.78760	1540.56285	770.78506	E	781.34752	391.17740	6
14	1675.63125	838.31926	1703.62617	852.31672	Y	652.30492	326.65610	5
15	1789.67418	895.34073	1817.66910	909.33819	N	489.24160	245.12444	4
16	1903.71711	952.36219	1931.71203	966.35965	N	375.19867	188.10297	3
17	2017.76004	1009.38366	2045.75496	1023.38112	N	261.15574	131.08151	2
18					K	147.11281	74.06004	1

Fragment Spectrum

Extracted from: C:\Users\SAH\Desktop\Data\Suraya\V2085_EBA181_RH4_tryp_chymo\V2086_EBA181_P_chy_200fmol_2ul_1_400_43C_70m09233648H4C4.raw #5905 RT: 43.25
FTMS, HCD, z=+2, Mono m/z=1096.43347 Da, MH+=2191.85967 Da, Match Tol.=20 mmu

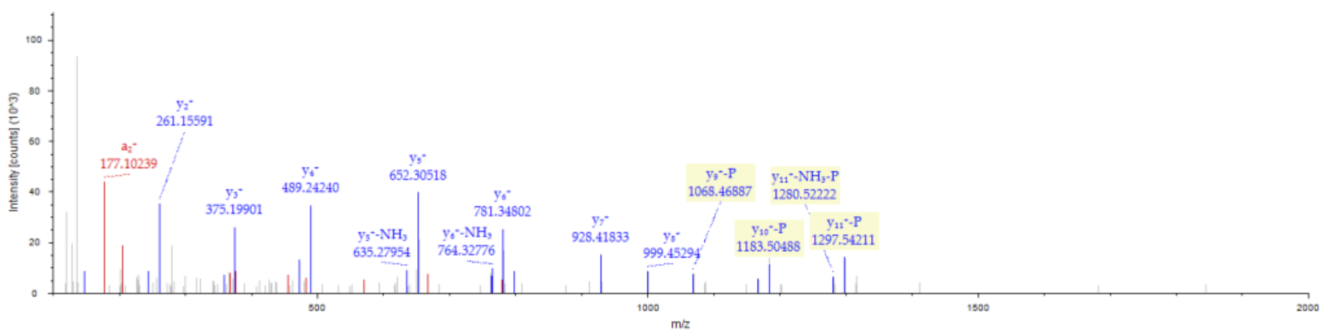

Sequence: GFYDSNLNDSAFEYNNNK
S10-Phospho (79.96633 Da)
Charge: +2, Monoisotopic m/z: 1096.43347 Da (-0.41 mmu/-0.38 ppm), MH+: 2191.85967 Da, RT: 43.25 min,

Extracted from: C:\Users\SAH\Desktop\Data\Suraya\V2085_EBA181_RH4_tryp_chymo\V2086_EBA181_P_chy_200fmol_2ul_1_400_43C_70m09233648H4C4.raw #5905 RT: 43.25
FTMS, HCD, z=+2, Mono m/z=1096.43347 Da, MH+=2191.85967 Da, Match Tol.=20 mmu

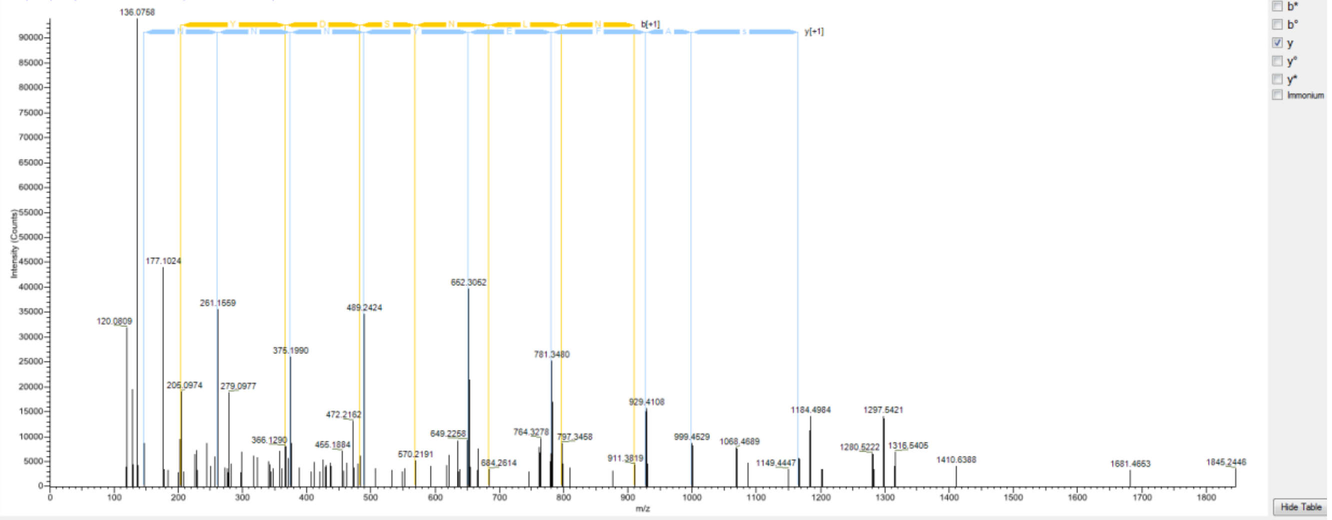

	(NH3+) -	F> <Y	Y> <D	D> <S	S> <N	N> <L	L> <N	N> <D	D> <s	s> <A	A> <F	F> <E	E> <Y	Y> <N	N> <N	N> <N	N> <K	K - (COOH)
b (+1)	58.02875	205.09717	368.16049	483.18744	570.21947	684.26240	797.34647	911.38940	1026.41635	1193.41471	1264.45183	1411.52025	1540.56285	1703.62617	1817.66910	1931.71203	2045.75496	2173.84993
b*(+1)	41.00220	188.07062	351.13394	466.16089	553.19292	667.23585	780.31992	894.36285	1009.38980	1176.38816	1247.42528	1394.49370	1523.53630	1686.59962	1800.64255	1914.68548	2028.72841	2156.82338
b°(+1)	40.01818	187.08660	350.14992	465.17687	552.20890	666.25183	779.33590	893.37883	1008.40578	1175.40414	1246.44126	1393.50968	1522.55228	1685.61560	1799.65853	1913.70146	2027.74439	2155.83936
y (+1)	2134.83902	1987.77060	1824.70728	1709.68033	1622.64830	1508.60537	1395.52130	1281.47837	1166.45142	999.45306	928.41594	781.34752	652.30492	489.24160	375.19867	261.15574	147.11281	2191.86049
y*(+1)	2117.81247	1970.74405	1807.68073	1692.65378	1605.62175	1491.57882	1378.49475	1264.45182	1149.42487	982.42651	911.38939	764.32097	635.27837	472.21505	358.17212	244.12919	130.08626	2174.83394
y°(+1)	2116.82846	1969.76004	1806.69672	1691.66977	1604.63774	1490.59481	1377.51074	1263.46781	1148.44086	981.44250	910.40538	763.33696	634.29436	471.23104	357.18811	243.14518	129.10225	2173.84993

Value Type: Theo. Mass [Da]

G F⌋Y⌋D⌋S⌋N⌋L⌋N⌋D⌈**s**⌈A⌈F⌈E⌈Y⌈N⌈N⌈N⌈K

G. EBA181_14

Peptide Summary

Sequence: GFYDSNLNDSAFEYNNNKY, S10-Phospho (79.96633 Da)
Charge: +2, Monoisotopic m/z: 1177.96436 Da (-1.19 mmu/-1.01 ppm), MH+: 2354.92143 Da, RT: 46.58 min,
Identified with: Mascot (v1.27); IonScore:36, Exp Value:3.3E-004, Ions matched by search engine: 6/194
Fragment match tolerance used for search: 20 mmu

Fragment Matches

Value Type: Theo. Mass [Da]

Ion Series | Phosphorylation Losses | Neutral Losses | Multiple Neutral Losses | Precursor Ions

#1	a+	a2+	b+	b2+	Seq.	y+	y2+	#2
1	30.03383	15.52055	58.02875	29.51801	G			19
2	177.10225	89.05476	205.09717	103.05222	F	2297.90234	1149.45481	18
3	340.16557	170.58642	368.16049	184.58388	Y	2150.83392	1075.92060	17
4	455.19252	228.09990	483.18744	242.09736	D	1987.77060	994.38894	16
5	542.22455	271.61591	570.21947	285.61337	S	1872.74365	936.87546	15
6	656.26748	328.63738	684.26240	342.63484	N	1785.71162	893.35945	14
7	769.35155	385.17941	797.34647	399.17687	L	1671.66869	836.33798	13
8	883.39448	442.20088	911.38940	456.19834	N	1558.58462	779.79595	12
9	998.42143	499.71435	1026.41635	513.71181	D	1444.54169	722.77448	11
10	1165.41979	583.21353	1193.41471	597.21099	S-Phospho	1329.51474	665.26101	10
11	1236.45691	618.73209	1264.45183	632.72955	A	1162.51638	581.76183	9
12	1383.52533	692.26630	1411.52025	706.26376	F	1091.47926	546.24327	8
13	1512.56793	756.78760	1540.56285	770.78506	E	944.41084	472.70906	7
14	1675.63125	838.31926	1703.62617	852.31672	Y	815.36824	408.18776	6
15	1789.67418	895.34073	1817.66910	909.33819	N	652.30492	326.65610	5
16	1903.71711	952.36219	1931.71203	966.35965	N	538.26199	269.63463	4
17	2017.76004	1009.38366	2045.75496	1023.38112	N	424.21906	212.61317	3
18	2145.85501	1073.43114	2173.84993	1087.42860	K	310.17613	155.59170	2
19					Y	182.08116	91.54422	1

Fragment Spectrum

Extracted from: C:\Users\SAH\Desktop\Data\Suraya\V2085_EBA181_RH4_tryp_chymo\V2085_EBA181_P_tryp_2pmols_43C_70m09233648H4C4.raw #7918 RT: 46.58
FTMS, HCD, z=+2, Mono m/z=1177.96436 Da, MH+=2354.92143 Da, Match Tol.=20 mmu

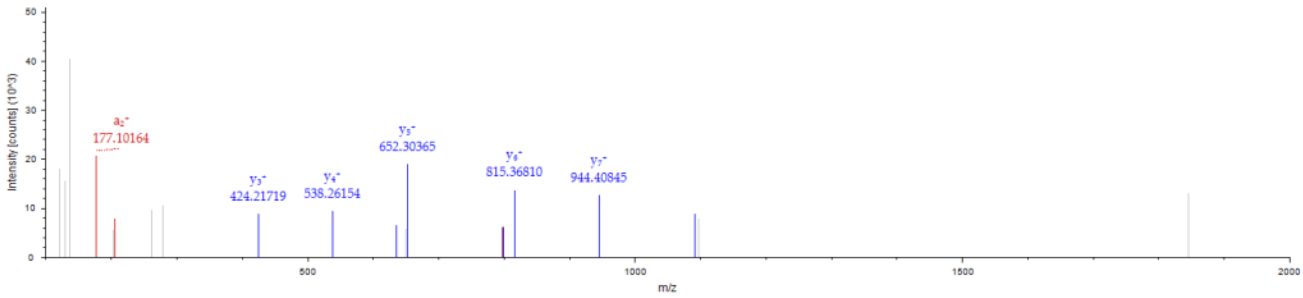

Sequence: GFYDSNLNDSAFEYNNNKY
S10-Phospho (79.96633 Da)
Charge: +2, Monoisotopic m/z: 1177.96436 Da (-1.19 mmu/-1.01 ppm), MH+: 2354.92143 Da, RT: 46.58 min,

Extracted from: C:\Users\SAH\Desktop\Data\Suraya\V2085_EBA181_RH4_tryp_chymo\V2085_EBA181_P_tryp_2pmols_43C_70m09233648H4C4.raw #7918 RT: 46.58
FTMS, HCD, z=+2, Mono m/z=1177.96436 Da, MH+=2354.92143 Da, Match Tol.=20 mmu

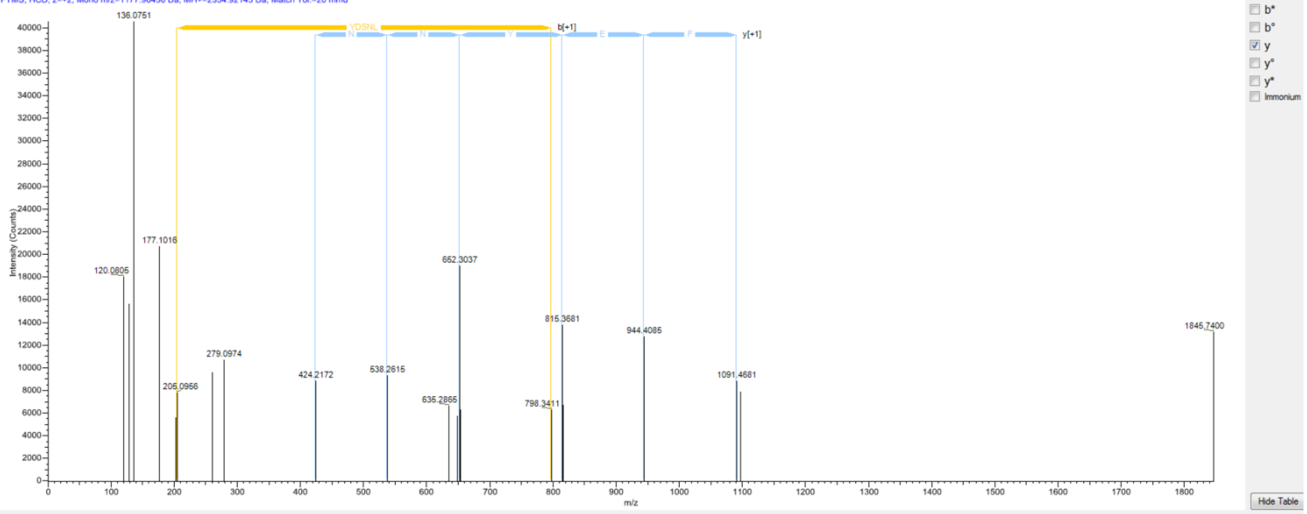

	(NH3+) -	F> <Y	Y> <D	D> <S	S> <N	N> <L	L> <N	N> <D	D> <s	s> <A	A> <F	F> <E	E> <Y	Y> <N	N> <N	N> <N	N> <K	K> <Y	Y - (COOH)
b (+1)	58.02875	205.09717	368.16049	483.18744	570.21947	684.26240	797.34647	911.38940	1026.41635	1193.41471	1264.45183	1411.52025	1540.56285	1703.62617	1817.66910	1931.71203	2045.75496	2173.84993	2336.91325
b*(+1)	41.00220	188.07062	351.13394	466.16089	553.19292	667.23585	780.31992	894.36285	1009.38980	1176.38816	1247.42528	1394.49370	1523.53630	1686.59962	1800.64255	1914.68548	2028.72841	2156.82338	2319.88670
b°(+1)	40.01818	187.08660	350.14992	465.17687	552.20890	666.25183	779.33590	893.37883	1008.40578	1175.40414	1246.44126	1393.50968	1522.55228	1685.61560	1799.65853	1913.70146	2027.74439	2155.83936	2318.90268
y (+1)	2297.90234	2150.83392	1987.77060	1872.74365	1785.71162	1671.66869	1558.58462	1444.54169	1329.51474	1162.51638	1091.47926	944.41084	815.36824	652.30492	538.26199	424.21906	310.17613	182.08116	2354.92381
y*(+1)	2280.87579	2133.80737	1970.74405	1855.71710	1768.68507	1654.64214	1541.55807	1427.51514	1312.48819	1145.48983	1074.45271	927.38429	798.34169	635.27837	521.23544	407.19251	293.14958	165.05461	2337.89726
y°(+1)	2279.89178	2132.82336	1969.76004	1854.73309	1767.70106	1653.65813	1540.57406	1426.53113	1311.50418	1144.50582	1073.46870	926.40028	797.35768	634.29436	520.25143	406.20850	292.16557	164.07060	2336.91325

Value Type: Theo. Mass [Da]

G F⌋Y D S N L⌋N D ***s*** A⌈F⌈E⌈Y⌈N⌈N⌈N K Y

G. EBA181_15

Peptide Summary

Sequence: GFYDSNLNDSAFEYNNNKYN, S10-Phospho (79.96633 Da)
Charge: +2, Monoisotopic m/z: 1234.98474 Da (-2.27 mmu/-1.84 ppm), MH+: 2468.96221 Da, RT: 45.05 min,
Identified with: Mascot (v1.27); IonScore:60, Exp Value:1.3E-006, Ions matched by search engine: 9/208
Fragment match tolerance used for search: 20 mmu

Fragment Matches

Value Type: Theo. Mass [Da]

Ion Series | Phosphorylation Losses | Neutral Losses | Multiple Neutral Losses | Precursor Ions

#1	a^+	a^{2+}	b^+	b^{2+}	Seq.	y^+	y^{2+}	#2
1	30.03383	15.52055	58.02875	29.51801	G			20
2	177.10225	89.05476	205.09717	103.05222	F	2411.94527	1206.47627	19
3	340.16557	170.58642	368.16049	184.58388	Y	2264.87685	1132.94206	18
4	455.19252	228.09990	483.18744	242.09736	D	2101.81353	1051.41040	17
5	542.22455	271.61591	570.21947	285.61337	S	1986.78658	993.89693	16
6	656.26748	328.63738	684.26240	342.63484	N	1899.75455	950.38091	15
7	769.35155	385.17941	797.34647	399.17687	L	1785.71162	893.35945	14
8	883.39448	442.20088	911.38940	456.19834	N	1672.62755	836.81741	13
9	998.42143	499.71435	1026.41635	513.71181	D	1558.58462	779.79595	12
10	1165.41979	583.21353	1193.41471	597.21099	S-Phospho	1443.55767	722.28247	11
11	1236.45691	618.73209	1264.45183	632.72955	A	1276.55931	638.78329	10
12	1383.52533	692.26630	1411.52025	706.26376	F	1205.52219	603.26473	9
13	1512.56793	756.78760	1540.56285	770.78506	E	1058.45377	529.73052	8
14	1675.63125	838.31926	1703.62617	852.31672	Y	929.41117	465.20922	7
15	1789.67418	895.34073	1817.66910	909.33819	N	766.34785	383.67756	6
16	1903.71711	952.36219	1931.71203	966.35965	N	652.30492	326.65610	5
17	2017.76004	1009.38366	2045.75496	1023.38112	N	538.26199	269.63463	4
18	2145.85501	1073.43114	2173.84993	1087.42860	K	424.21906	212.61317	3
19	2308.91833	1154.96280	2336.91325	1168.96026	Y	296.12409	148.56568	2
20					N	133.06077	67.03402	1

Fragment Spectrum

Extracted from: C:\Users\SAH\Desktop\Data\Suraya\V2085_EBA181_RH4_tryp_chymo\V2085_EBA181_P_tryp_2pmols_43C_70m09233648H4C4.raw #7559 RT: 45.05
FTMS, HCD, z=+2, Mono m/z=1234.98474 Da, MH+=2468.96221 Da, Match Tol.=20 mmu

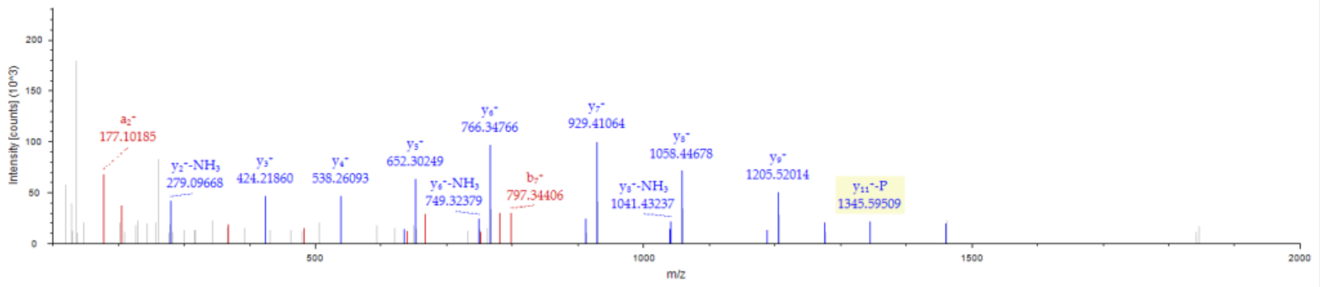

Sequence: GFYDSNLNDSAFEYNNNKYN
S10-Phospho (79.96633 Da)
Charge: +2, Monoisotopic m/z: 1234.98474 Da (-2.27 mmu/-1.84 ppm), MH+: 2468.96221 Da, RT: 45.05 min,

Extracted from: C:\Users\SAH\Desktop\Data\Suraya\V2085_EBA181_RH4_tryp_chymo\V2085_EBA181_P_tryp_2pmols_43C_70m09233648H4C4.raw #7559 RT: 45.05
FTMS, HCD, z=+2, Mono m/z=1234.98474 Da, MH+=2468.96221 Da, Match Tol.=20 mmu

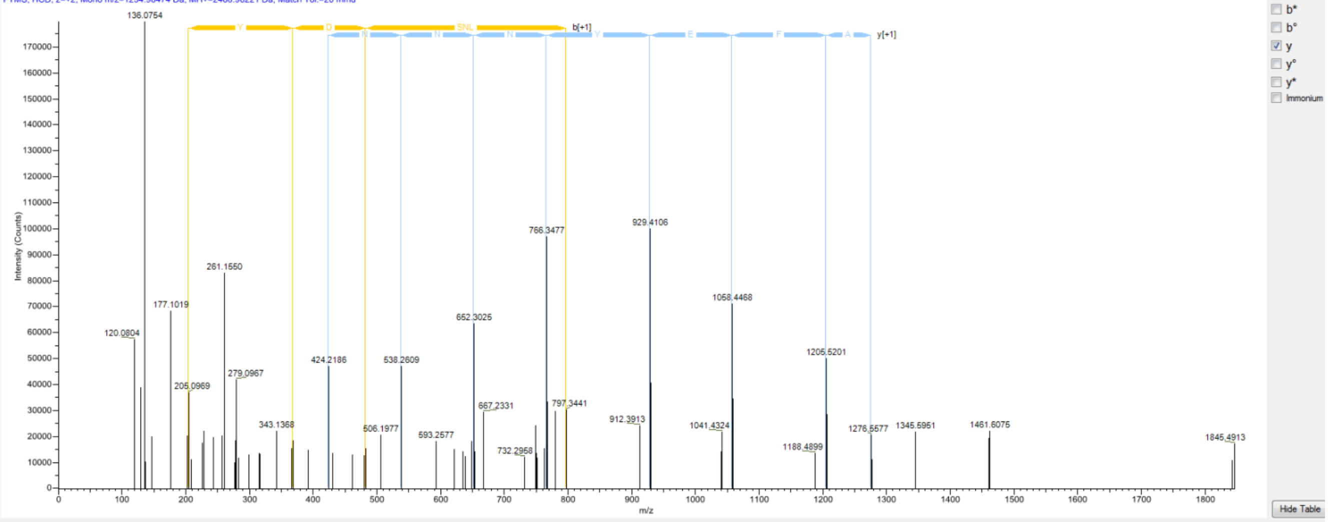

	(NH3+) -	F> <Y	Y> <D	D> <S	S> <N	N> <L	L> <N	N> <D	D> <s	s> <A	A> <F	F> <E	E> <Y	Y> <N	N> <N	N> <N	N> <K	K> <Y	Y> <N	N - (COOH)
b (+1)	58.02875	205.09717	368.16049	483.18744	570.21947	684.26240	797.34647	911.38940	1026.41635	1193.41471	1264.45183	1411.52025	1540.56285	1703.62617	1817.66910	1931.71203	2045.75496	2173.84993	2336.91325	2450.95618
b*(+1)	41.00220	188.07062	351.13394	466.16089	553.19292	667.23585	780.31992	894.36285	1009.38980	1176.38816	1247.42528	1394.49370	1523.53630	1686.59962	1800.64255	1914.68548	2028.72841	2156.82338	2319.88670	2433.92963
b°(+1)	40.01818	187.08660	350.14992	465.17687	552.20890	666.25183	779.33590	893.37883	1008.40578	1175.40414	1246.44126	1393.50968	1522.55228	1685.61560	1799.65853	1913.70146	2027.74439	2155.83936	2318.90268	2432.94561
y (+1)	2411.94527	2264.87685	2101.81353	1986.78658	1899.75455	1785.71162	1672.62755	1558.58462	1443.55767	1276.55931	1205.52219	1058.45377	929.41117	766.34785	652.30492	538.26199	424.21906	296.12409	133.06077	2468.96674
y*(+1)	2394.91872	2247.85030	2084.78698	1969.76003	1882.72800	1768.68507	1655.60100	1541.55807	1426.53112	1259.53276	1188.49564	1041.42722	912.38462	749.32130	635.27837	521.23544	407.19251	279.09754	116.03422	2451.94019
y°(+1)	2393.93471	2246.86629	2083.80297	1968.77602	1881.74399	1767.70106	1654.61699	1540.57406	1425.54711	1258.54875	1187.51163	1040.44321	911.40061	748.33729	634.29436	520.25143	406.20850	278.11353	115.05021	2450.95618

Value Type: Theo. Mass [Da]

G F⌋Y⌋D⌋S N L⌋N D **s**⌈A⌈F⌈E⌈Y⌈N⌈N⌈N⌈K Y N

G. EBA181_16

Peptide Summary

Sequence: GFYDSNLNDSAFEYNNNK, S10-Phospho (79.96633 Da)
Charge: +2, Monoisotopic m/z: 1096.43408 Da (+0.2 mmu/+0.18 ppm), MH+: 2191.86089 Da, RT: 44.12 min,
Identified with: Mascot (v1.27); IonScore:83, Exp Value:1.2E-008, Ions matched by search engine: 10/184
Fragment match tolerance used for search: 20 mmu

Fragment Matches

Value Type: Theo. Mass [Da]

Ion Series | Phosphorylation Losses | Neutral Losses | Multiple Neutral Losses | Precursor Ions

#1	a⁺	a²⁺	b⁺	b²⁺	Seq.	y⁺	y²⁺	#2
1	30.03383	15.52055	58.02875	29.51801	G			18
2	177.10225	89.05476	205.09717	103.05222	F	2134.83902	1067.92315	17
3	340.16557	170.58642	368.16049	184.58388	Y	1987.77060	994.38894	16
4	455.19252	228.09990	483.18744	242.09736	D	1824.70728	912.85728	15
5	542.22455	271.61591	570.21947	285.61337	S	1709.68033	855.34380	14
6	656.26748	328.63738	684.26240	342.63484	N	1622.64830	811.82779	13
7	769.35155	385.17941	797.34647	399.17687	L	1508.60537	754.80632	12
8	883.39448	442.20088	911.38940	456.19834	N	1395.52130	698.26429	11
9	998.42143	499.71435	1026.41635	513.71181	D	1281.47837	641.24282	10
10	1165.41979	583.21353	1193.41471	597.21099	S-Phospho	1166.45142	583.72935	9
11	1236.45691	618.73209	1264.45183	632.72955	A	999.45306	500.23017	8
12	1383.52533	692.26630	1411.52025	706.26376	F	928.41594	464.71161	7
13	1512.56793	756.78760	1540.56285	770.78506	E	781.34752	391.17740	6
14	1675.63125	838.31926	1703.62617	852.31672	Y	652.30492	326.65610	5
15	1789.67418	895.34073	1817.66910	909.33819	N	489.24160	245.12444	4
16	1903.71711	952.36219	1931.71203	966.35965	N	375.19867	188.10297	3
17	2017.76004	1009.38366	2045.75496	1023.38112	N	261.15574	131.08151	2
18					K	147.11281	74.06004	1

Fragment Spectrum

Extracted from: C:\Users\SAH\Desktop\Data\Suraya\V2085_EBA181_RH4_tryp_chymo\V2085_EBA181_P_tryp_2pmols_43C_70m09233648H4C4.raw #7340 RT: 44.12
FTMS, HCD, z=+2, Mono m/z=1096.43408 Da, MH+=2191.86089 Da, Match Tol.=20 mmu

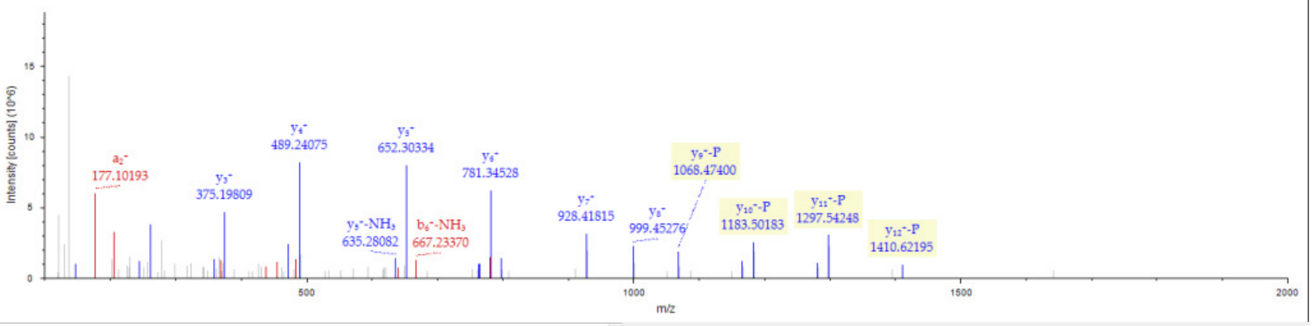

Sequence: GFYDSNLNDSAFEYNNNK
S10-Phospho (79.96633 Da)
Charge: +2, Monoisotopic m/z: 1096.43408 Da (+0.2 mmu/+0.18 ppm), MH+: 2191.86089 Da, RT: 44.12 min,

Extracted from: C:\Users\SAH\Desktop\Data\Suraya\V2085_EBA181_RH4_tryp_chymo\V2085_EBA181_P_tryp_2pmols_43C_70m09233648H4C4.raw #7340 RT: 44.12
FTMS, HCD, z=+2, Mono m/z=1096.43408 Da, MH+=2191.86089 Da, Match Tol.=20 mmu

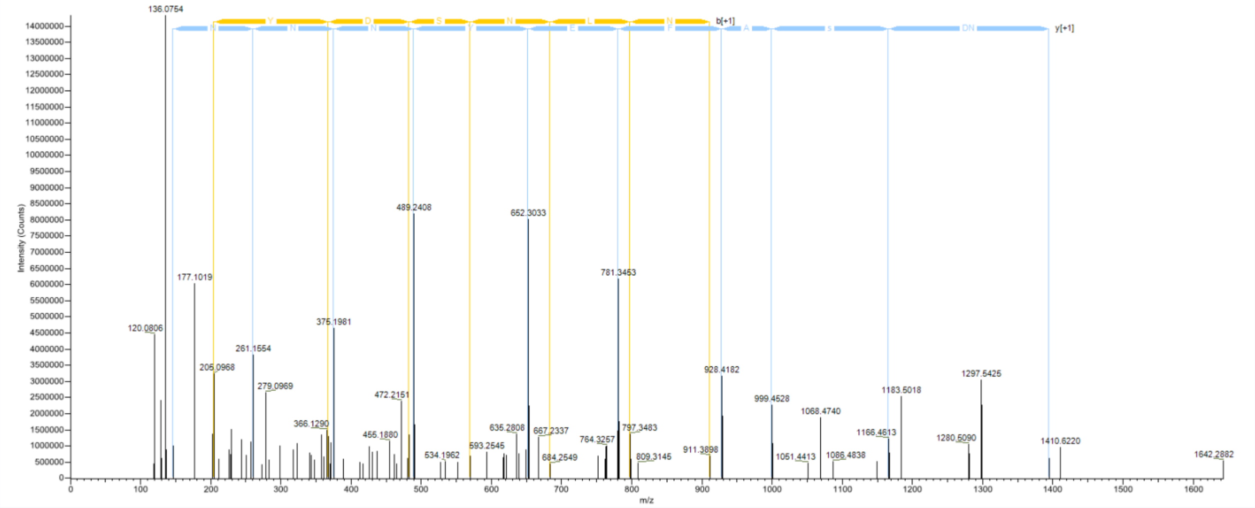

b
b*
b°
y
y*
y°
Immonium

Hide Table

	(NH3+) -	F> <Y	Y> <D	D> <S	S> <N	N> <L	L> <N	N> <D	D> <s	s> <A	A> <F	F> <E	E> <Y	Y> <N	N> <N	N> <N	N> <K	K - (COOH)
b (+1)	58.02875	205.09717	368.16049	483.18744	570.21947	684.26240	797.34647	911.38940	1026.41635	1193.41471	1264.45183	1411.52025	1540.56285	1703.62617	1817.66910	1931.71203	2045.75496	2173.84993
b*(+1)	41.00220	188.07062	351.13394	466.16089	553.19292	667.23585	780.31992	894.36285	1009.38980	1176.38816	1247.42528	1394.49370	1523.53630	1686.59962	1800.64255	1914.68548	2028.72841	2156.82338
b°(+1)	40.01818	187.08660	350.14992	465.17687	552.20890	666.25183	779.33590	893.37883	1008.40578	1175.40414	1246.44126	1393.50968	1522.55228	1685.61560	1799.65853	1913.70146	2027.74439	2155.83936
y (+1)	2134.83902	1987.77060	1824.70728	1709.68033	1622.64830	1508.60537	1395.52130	1281.47837	1166.45142	999.45306	928.41594	781.34752	652.30492	489.24160	375.19867	261.15574	147.11281	2191.86049
y*(+1)	2117.81247	1970.74405	1807.68073	1692.65378	1605.62175	1491.57882	1378.49475	1264.45182	1149.42487	982.42651	911.38939	764.32097	635.27837	472.21505	358.17212	244.12919	130.08626	2174.83394
y°(+1)	2116.82846	1969.76004	1806.69672	1691.66977	1604.63774	1490.59481	1377.51074	1263.46781	1148.44086	981.44250	910.40538	763.33696	634.29436	471.23104	357.18811	243.14518	129.10225	2173.84993

Value Type: Theo. Mass [Da]

G F⌋Y⌋D⌋S⌋N⌋L⌈N⌋D⌈**s**⌈A⌈F⌈E⌈Y⌈N⌈N⌈N⌈K

G. EBA181_17

Peptide Summary

Sequence: FYDSNLNDSAFEYNNNK, S9-Phospho (79.96633 Da)
Charge: +2, Monoisotopic m/z: 1067.92114 Da (-2.01 mmu/-1.88 ppm), MH+: 2134.83501 Da, RT: 41.02 min,
Identified with: Mascot (v1.27); IonScore:80, Exp Value:2.2E-008, Ions matched by search engine: 11/176
Fragment match tolerance used for search: 20 mmu

Fragment Matches

Value Type: Theo. Mass [Da]

Ion Series | Phosphorylation Losses | Neutral Losses | Multiple Neutral Losses | Precursor Ions

#1	a⁺	a²⁺	b⁺	b²⁺	Seq.	y⁺	y²⁺	#2
1	120.08078	60.54403	148.07570	74.54149	F			17
2	283.14410	142.07569	311.13902	156.07315	Y	1987.77060	994.38894	16
3	398.17105	199.58916	426.16597	213.58662	D	1824.70728	912.85728	15
4	485.20308	243.10518	513.19800	257.10264	S	1709.68033	855.34380	14
5	599.24601	300.12664	627.24093	314.12410	N	1622.64830	811.82779	13
6	712.33008	356.66868	740.32500	370.66614	L	1508.60537	754.80632	12
7	826.37301	413.69014	854.36793	427.68760	N	1395.52130	698.26429	11
8	941.39996	471.20362	969.39488	485.20108	D	1281.47837	641.24282	10
9	1108.39832	554.70280	1136.39324	568.70026	S-Phospho	1166.45142	583.72935	9
10	1179.43544	590.22136	1207.43036	604.21882	A	999.45306	500.23017	8
11	1326.50386	663.75557	1354.49878	677.75303	F	928.41594	464.71161	7
12	1455.54646	728.27687	1483.54138	742.27433	E	781.34752	391.17740	6
13	1618.60978	809.80853	1646.60470	823.80599	Y	652.30492	326.65610	5
14	1732.65271	866.82999	1760.64763	880.82745	N	489.24160	245.12444	4
15	1846.69564	923.85146	1874.69056	937.84892	N	375.19867	188.10297	3
16	1960.73857	980.87292	1988.73349	994.87038	N	261.15574	131.08151	2
17					K	147.11281	74.06004	1

Fragment Spectrum

Extracted from: C:\Users\SAH\Desktop\Data\Suraya\V2085_EBA181_RH4_tryp_chymo\V2085_EBA181_P_tryp_2pmols_43C_70m09233648H4C4.raw #6611 RT: 41.02
FTMS, HCD, z=+2, Mono m/z=1067.92114 Da, MH+=2134.83501 Da, Match Tol.=20 mmu

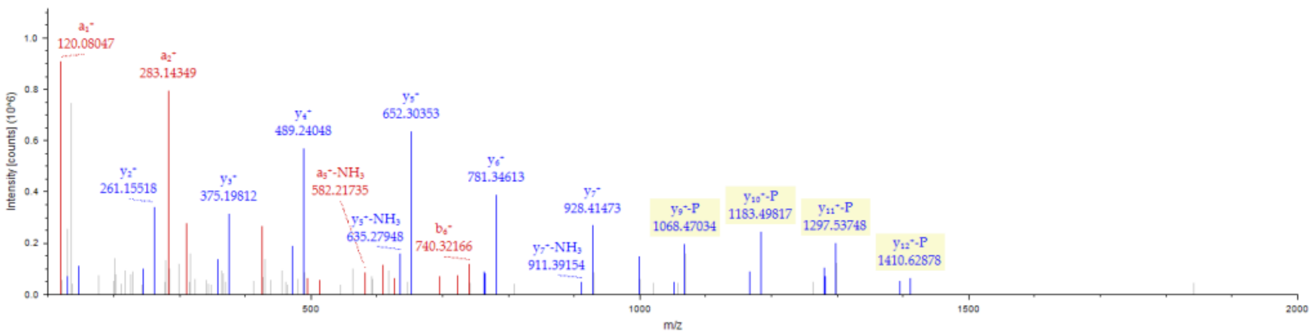

Sequence: FYDSNLNDSAFEYNNNK
S9-Phospho (79.96633 Da)
Charge: +2, Monoisotopic m/z: 1067.92114 Da (-2.01 mmu/-1.88 ppm), MH+: 2134.83501 Da, RT: 41.02 min,

Extracted from: C:\Users\SAH\Desktop\Data\Suraya\V2085_EBA181_RH4_tryp_chymo\V2085_EBA181_P_tryp_2pmols_43C_70m09233648H4C4.raw #6611 RT: 41.02
FTMS, HCD, z=+2, Mono m/z=1067.92114 Da, MH+=2134.83501 Da, Match Tol.=20 mmu

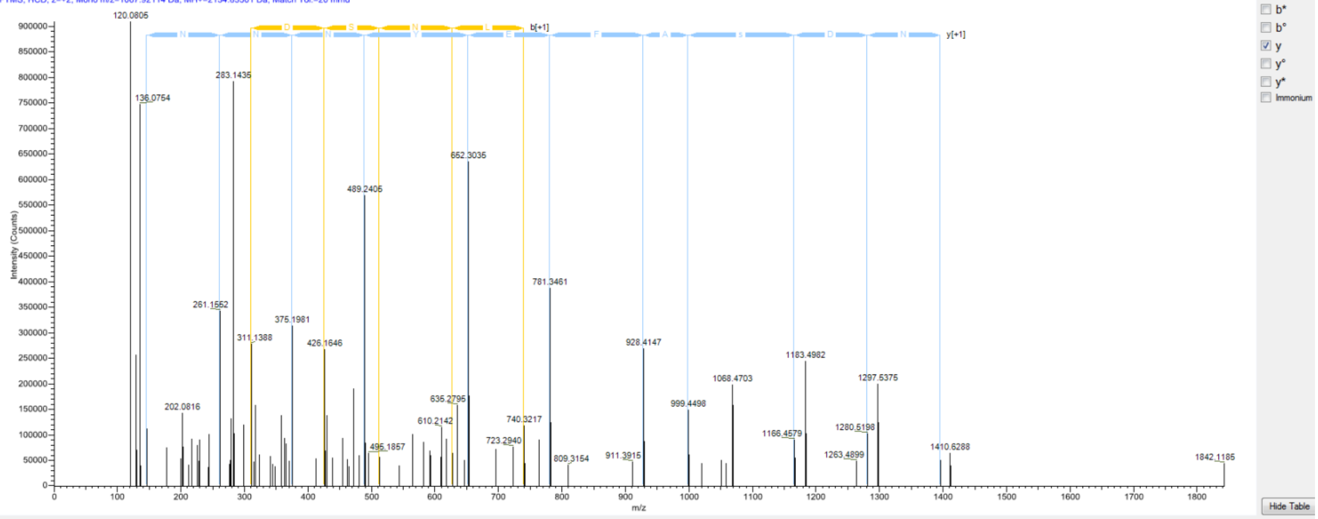

	(NH3+) - F>	Y> <D	D> <S	S> <N	N> <L	L> <N	N> <D	D> <s	s> <A	A> <F	F> <E	E> <Y	Y> <N	N> <N	N> <N	N> <K	K - (COOH)
b (+1)	148.07570	311.13902	426.16597	513.19800	627.24093	740.32500	854.36793	969.39488	1136.39324	1207.43036	1354.49878	1483.54138	1646.60470	1760.64763	1874.69056	1988.73349	2116.82846
b*(+1)	131.04915	294.11247	409.13942	496.17145	610.21438	723.29845	837.34138	952.36833	1119.36669	1190.40381	1337.47223	1466.51483	1629.57815	1743.62108	1857.66401	1971.70694	2099.80191
b°(+1)	130.06513	293.12845	408.15540	495.18743	609.23036	722.31443	836.35736	951.38431	1118.38267	1189.41979	1336.48821	1465.53081	1628.59413	1742.63706	1856.67999	1970.72292	2098.81789
y (+1)	1987.77060	1824.70728	1709.68033	1622.64830	1508.60537	1395.52130	1281.47837	1166.45142	999.45306	928.41594	781.34752	652.30492	489.24160	375.19867	261.15574	147.11281	2134.83902
y*(+1)	1970.74405	1807.68073	1692.65378	1605.62175	1491.57882	1378.49475	1264.45182	1149.42487	982.42651	911.38939	764.32097	635.27837	472.21505	358.17212	244.12919	130.08626	2117.81247
y°(+1)	1969.76004	1806.69672	1691.66977	1604.63774	1490.59481	1377.51074	1263.46781	1148.44086	981.44250	910.40538	763.33696	634.29436	471.23104	357.18811	243.14518	129.10225	2116.82846

Value Type: Theo. Mass [Da]

F Y D S N L N D s A F E Y N N N K

G. EBA181_18

Peptide Summary

Sequence: LNDSAFEYNNNK, S4-Phospho (79.96633 Da)
Charge: +2, Monoisotopic m/z: 754.80463 Da (-1.7 mmu/-2.25 ppm), MH+: 1508.60198 Da, RT: 29.08 min,
Identified with: Mascot (v1.27); IonScore:86, Exp Value:2.7E-009, Ions matched by search engine: 11/128
Fragment match tolerance used for search: 20 mmu

Fragment Matches

Value Type: Theo. Mass [Da]

Ion Series | Phosphorylation Losses | Neutral Losses | Multiple Neutral Losses | Precursor Ions

#1	a⁺	a²⁺	b⁺	b²⁺	Seq.	y⁺	y²⁺	#2
1	86.09643	43.55185	114.09135	57.54931	L			12
2	200.13936	100.57332	228.13428	114.57078	N	1395.52130	698.26429	11
3	315.16631	158.08679	343.16123	172.08425	D	1281.47837	641.24282	10
4	482.16467	241.58597	510.15959	255.58343	S-Phospho	1166.45142	583.72935	9
5	553.20179	277.10453	581.19671	291.10199	A	999.45306	500.23017	8
6	700.27021	350.63874	728.26513	364.63620	F	928.41594	464.71161	7
7	829.31281	415.16004	857.30773	429.15750	E	781.34752	391.17740	6
8	992.37613	496.69170	1020.37105	510.68916	Y	652.30492	326.65610	5
9	1106.41906	553.71317	1134.41398	567.71063	N	489.24160	245.12444	4
10	1220.46199	610.73463	1248.45691	624.73209	N	375.19867	188.10297	3
11	1334.50492	667.75610	1362.49984	681.75356	N	261.15574	131.08151	2
12					K	147.11281	74.06004	1

Fragment Spectrum

Extracted from: C:\Users\SAH\Desktop\Data\Suraya\V2085_EBA181_RH4_tryp_chymo\V2085_EBA181_P_tryp_2pmols_43C_70m09233648H4C4.raw #3926 RT: 29.08
FTMS, HCD, z=+2, Mono m/z=754.80463 Da, MH+=1508.60198 Da, Match Tol.=20 mmu

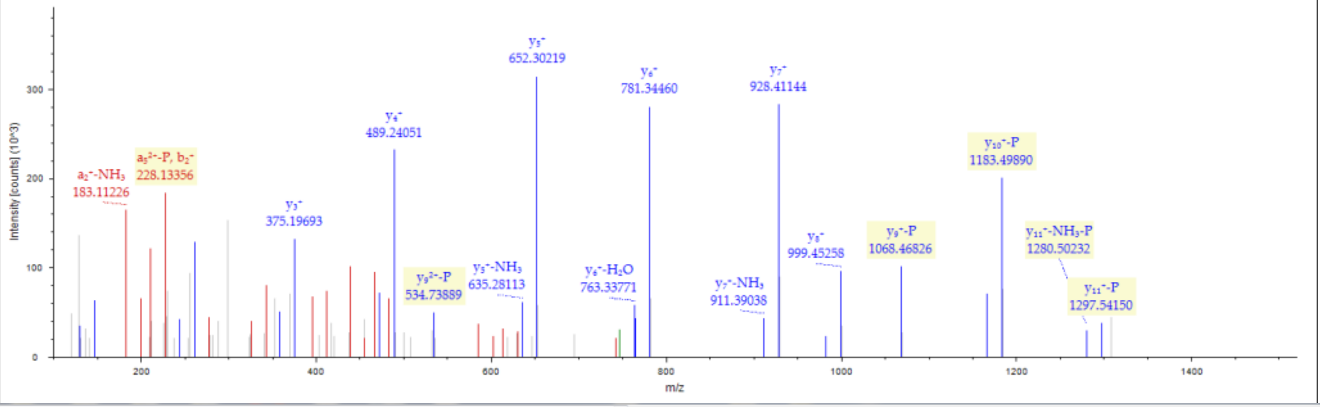

Sequence: LNDSAFEYNNNK
S4-Phospho (79.96633 Da)
Charge: +2, Monoisotopic m/z: 754.80463 Da (-1.7 mmu/-2.25 ppm), MH+: 1508.60198 Da, RT: 29.08 min,

Extracted from: C:\Users\SAH\Desktop\Data\Suraya\V2085_EBA181_RH4_tryp_chymo\V2085_EBA181_P_tryp_2pmols_43C_70m09233648H4C4.raw #3926 RT: 29.08
FTMS, HCD, z=+2, Mono m/z=754.80463 Da, MH+=1508.60198 Da, Match Tol.=20 mmu

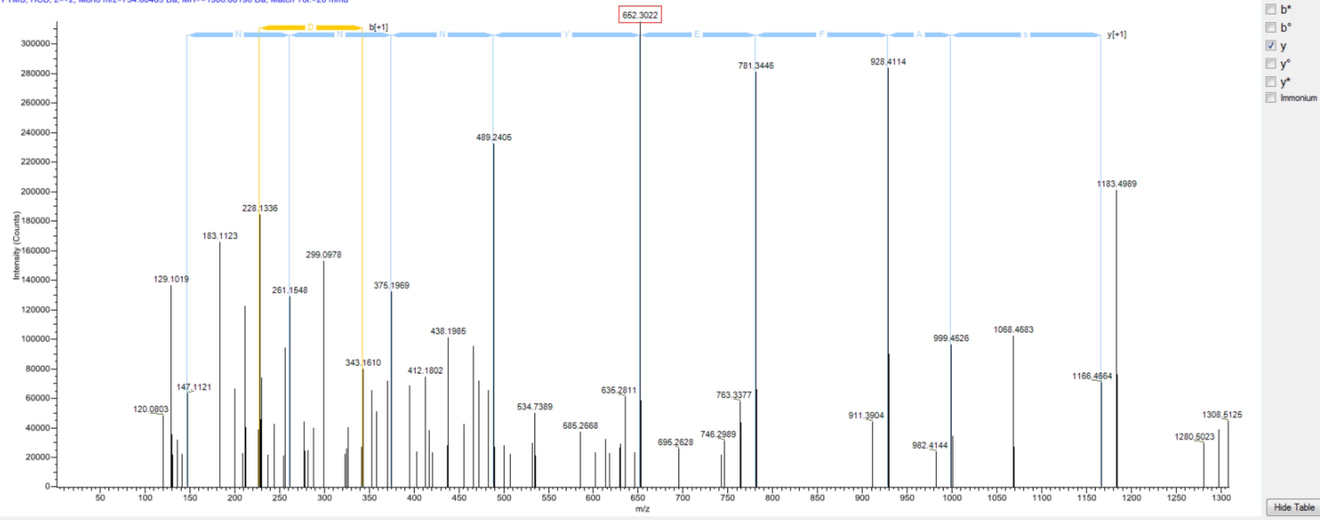

	(NH3+) - L>	N> <D	D> <s	s> <A	A> <F	F> <E	E>	Y> <N	N> <N	N> <N	N> <K	K - (COOH)
b (+1)	114.09135	228.13428	343.16123	510.15959	581.19671	728.26513	857.30773	1020.37105	1134.41398	1248.45691	1362.49984	1490.59481
b*(+1)	97.06480	211.10773	326.13468	493.13304	564.17016	711.23858	840.28118	1003.34450	1117.38743	1231.43036	1345.47329	1473.56826
b°(+1)	96.08078	210.12371	325.15066	492.14902	563.18614	710.25456	839.29716	1002.36048	1116.40341	1230.44634	1344.48927	1472.58424
y (+1)	1395.52130	1281.47837	1166.45142	999.45306	928.41594	781.34752	652.30492	489.24160	375.19867	261.15574	147.11281	1508.60537
y*(+1)	1378.49475	1264.45182	1149.42487	982.42651	911.38939	764.32097	635.27837	472.21505	358.17212	244.12919	130.08626	1491.57882
y°(+1)	1377.51074	1263.46781	1148.44086	981.44250	910.40538	763.33696	634.29436	471.23104	357.18811	243.14518	129.10225	1490.59481

Value Type: Theo. Mass [Da]

L N⌋ D⌋ s ⌈A ⌈F ⌈E ⌈Y ⌈N ⌈N ⌈N ⌈K

G. EBA181_19

Peptide Summary

Sequence: NDSAFEYNNNK, S3-Phospho (79.96633 Da)
Charge: +2, Monoisotopic m/z: 698.26398 Da (-0.31 mmu/-0.45 ppm), MH+: 1395.52068 Da, RT: 21.15 min,
Identified with: Mascot (v1.27); IonScore:51, Exp Value:8.1E-006, Ions matched by search engine: 9/120
Fragment match tolerance used for search: 20 mmu

Fragment Matches

Value Type: Theo. Mass [Da]

Ion Series | Phosphorylation Losses | Neutral Losses | Multiple Neutral Losses | Precursor Ions

#1	a⁺	a²⁺	b⁺	b²⁺	Seq.	y⁺	y²⁺	#2
1	87.05529	44.03128	115.05021	58.02874	N			11
2	202.08224	101.54476	230.07716	115.54222	D	1281.47837	641.24282	10
3	369.08060	185.04394	397.07552	199.04140	S-Phospho	1166.45142	583.72935	9
4	440.11772	220.56250	468.11264	234.55996	A	999.45306	500.23017	8
5	587.18614	294.09671	615.18106	308.09417	F	928.41594	464.71161	7
6	716.22874	358.61801	744.22366	372.61547	E	781.34752	391.17740	6
7	879.29206	440.14967	907.28698	454.14713	Y	652.30492	326.65610	5
8	993.33499	497.17113	1021.32991	511.16859	N	489.24160	245.12444	4
9	1107.37792	554.19260	1135.37284	568.19006	N	375.19867	188.10297	3
10	1221.42085	611.21406	1249.41577	625.21152	N	261.15574	131.08151	2
11					K	147.11281	74.06004	1

Fragment Spectrum

Extracted from: C:\Users\SAH\Desktop\Data\Suraya\V2085_EBA181_RH4_tryp_chymo\V2085_EBA181_P_tryp_2pmols_43C_70m09233648H4C4.raw #2246 RT: 21.15
FTMS, HCD, z=+2, Mono m/z=698.26398 Da, MH+=1395.52068 Da, Match Tol.=20 mmu

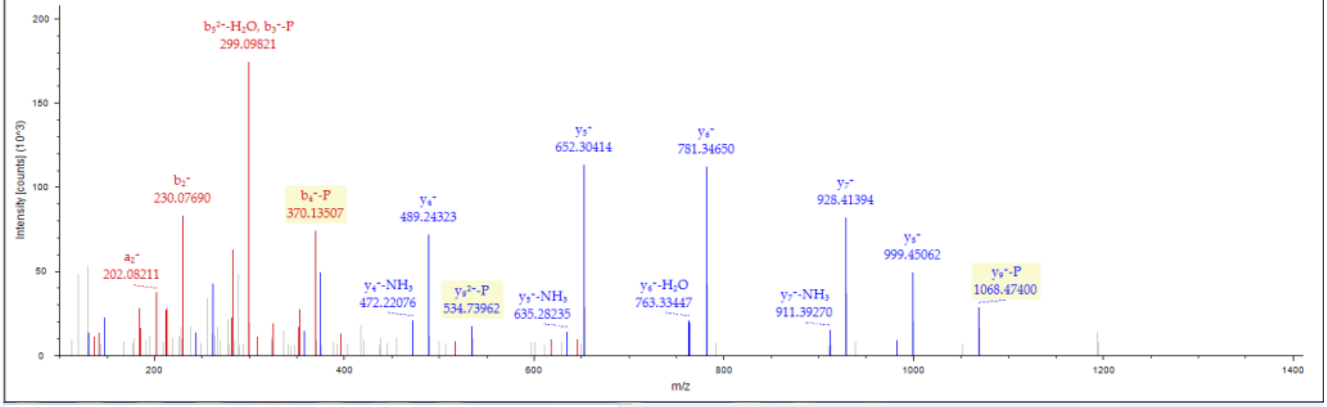

Sequence: NDSAFEYNNNK
S3-Phospho (79.96633 Da)
Charge: +2, Monoisotopic m/z: 698.26398 Da (-0.31 mmu/-0.45 ppm), MH+: 1395.52068 Da, RT: 21.15 min,

Extracted from: C:\Users\SAH\Desktop\Data\Suraya\V2085_EBA181_RH4_tryp_chymo\V2085_EBA181_P_tryp_2pmols_43C_70m09233648H4C4.raw #2246 RT: 21.15
FTMS, HCD, z=+2, Mono m/z=698.26398 Da, MH+=1395.52068 Da, Match Tol.=20 mmu

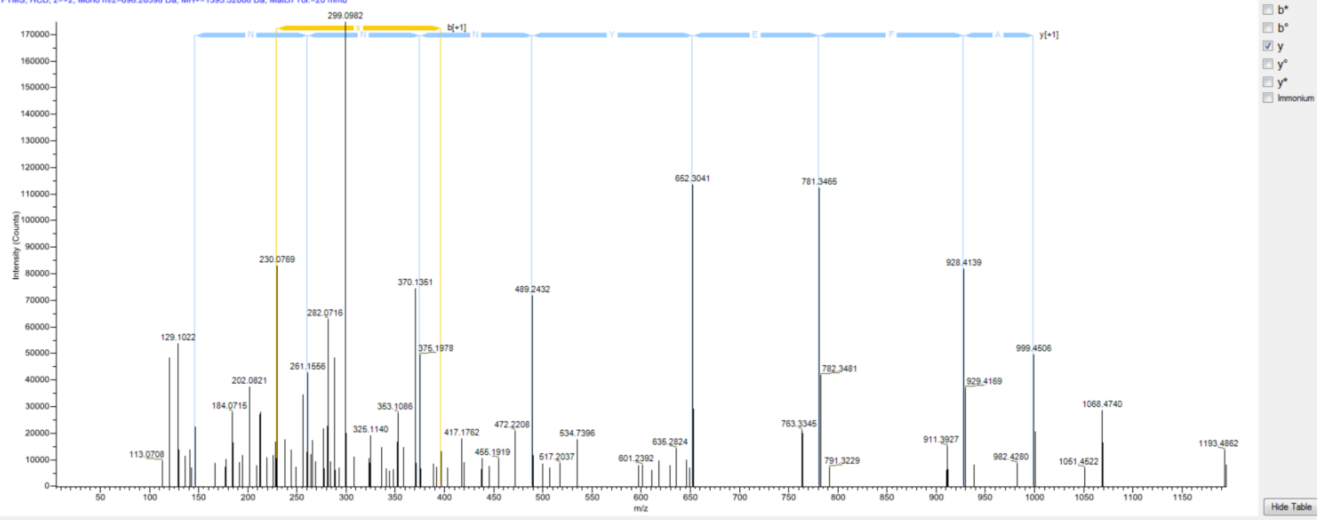

	(NH3+) -	D> <s	s> <A	A> <F	F> <E	E>	Y>	N> <N	N> <N	N> <K	K - (COOH)
b (+1)	115.05021	230.07716	397.07552	468.11264	615.18106	744.22366	907.28698	1021.32991	1135.37284	1249.41577	1377.51074
b*(+1)	98.02366	213.05061	380.04897	451.08609	598.15451	727.19711	890.26043	1004.30336	1118.34629	1232.38922	1360.48419
b°(+1)	97.03964	212.06659	379.06495	450.10207	597.17049	726.21309	889.27641	1003.31934	1117.36227	1231.40520	1359.50017
y (+1)	1281.47837	1166.45142	999.45306	928.41594	781.34752	652.30492	489.24160	375.19867	261.15574	147.11281	1395.52130
y*(+1)	1264.45182	1149.42487	982.42651	911.38939	764.32097	635.27837	472.21505	358.17212	244.12919	130.08626	1378.49475
y°(+1)	1263.46781	1148.44086	981.44250	910.40538	763.33696	634.29436	471.23104	357.18811	243.14518	129.10225	1377.51074

Value Type: Theo. Mass [Da]

N D s A F E Y N N N K

G. EBA181_20

Peptide Summary

Sequence: GFYDSNLNDSAFEYNNNK, S5-Phospho (79.96633 Da), S10-Phospho (79.96633 Da)
Charge: +3, Monoisotopic m/z: 757.94763 Da (+0.51 mmu/+0.67 ppm), MH+: 2271.82834 Da, RT: 49.67 min,
Identified with: Mascot (v1.27); IonScore:61, Exp Value:9.9E-007, Ions matched by search engine: 8/184
Fragment match tolerance used for search: 20 mmu

Fragment Matches

Value Type: Theo. Mass [Da]

Ion Series | Phosphorylation Losses | Neutral Losses | Multiple Neutral Losses | Precursor Ions

#1	a^+	a^{2+}	a^{3+}	b^+	b^{2+}	b^{3+}	Seq.	y^+	y^{2+}	y^{3+}	#2
1	30.03383	15.52055	10.68280	58.02875	29.51801	20.01443	G				18
2	177.10225	89.05476	59.70560	205.09717	103.05222	69.03724	F	2214.80535	1107.90631	738.93997	17
3	340.16557	170.58642	114.06004	368.16049	184.58388	123.39168	Y	2067.73693	1034.37210	689.91716	16
4	455.19252	228.09990	152.40236	483.18744	242.09736	161.73400	D	1904.67361	952.84044	635.56272	15
5	622.19088	311.59908	208.06848	650.18580	325.59654	217.40012	S-Phospho	1789.64666	895.32697	597.22041	14
6	736.23381	368.62054	246.08279	764.22873	382.61800	255.41443	N	1622.64830	811.82779	541.55429	13
7	849.31788	425.16258	283.77748	877.31280	439.16004	293.10912	L	1508.60537	754.80632	503.53998	12
8	963.36081	482.18404	321.79179	991.35573	496.18150	331.12343	N	1395.52130	698.26429	465.84529	11
9	1078.38776	539.69752	360.13411	1106.38268	553.69498	369.46574	D	1281.47837	641.24282	427.83098	10
10	1245.38612	623.19670	415.80023	1273.38104	637.19416	425.13186	S-Phospho	1166.45142	583.72935	389.48866	9
11	1316.42324	658.71526	439.47927	1344.41816	672.71272	448.81090	A	999.45306	500.23017	333.82254	8
12	1463.49166	732.24947	488.50207	1491.48658	746.24693	497.83371	F	928.41594	464.71161	310.14350	7
13	1592.53426	796.77077	531.51627	1620.52918	810.76823	540.84791	E	781.34752	391.17740	261.12069	6
14	1755.59758	878.30243	585.87071	1783.59250	892.29989	595.20235	Y	652.30492	326.65610	218.10649	5
15	1869.64051	935.32390	623.88502	1897.63543	949.32135	633.21666	N	489.24160	245.12444	163.75205	4
16	1983.68344	992.34536	661.89933	2011.67836	1006.34282	671.23097	N	375.19867	188.10297	125.73774	3
17	2097.72637	1049.36683	699.91364	2125.72129	1063.36428	709.24528	N	261.15574	131.08151	87.72343	2
18							K	147.11281	74.06004	49.70912	1

Fragment Spectrum

Extracted from: C:\Users\SAH\Desktop\Data\Suraya\V2085_EBA181_RH4_tryp_chymo\V2085_EBA181_P_tryp_2pmols_43C_70m09233648H4C4.raw #8636 RT: 49.67
FTMS, HCD, z=+3, Mono m/z=757.94763 Da, MH+=2271.82834 Da, Match Tol.=20 mmu

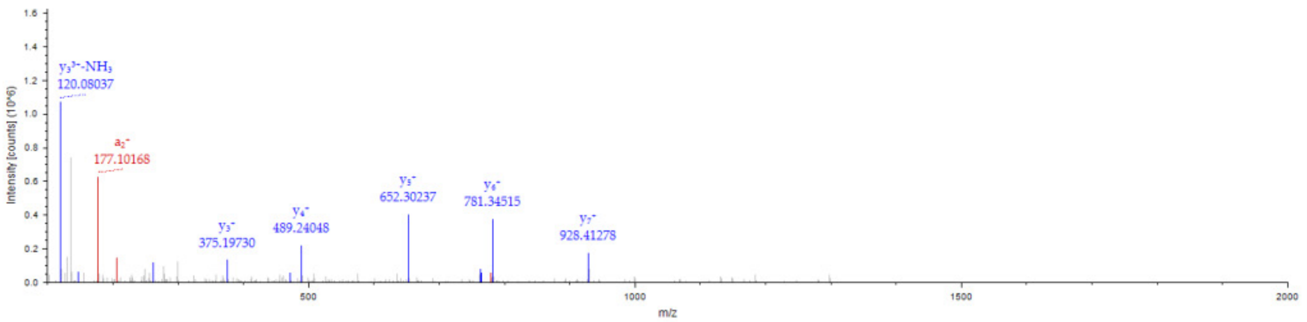

Sequence: GFYDSNLNDSAFEYNNNK
S5-Phospho (79.96633 Da), S10-Phospho (79.96633 Da)
Charge: +3, Monoisotopic m/z: 757.94763 Da (+0.51 mmu/+0.67 ppm), MH+: 2271.82834 Da, RT: 49.67 min,

Extracted from: C:\Users\SAH\Desktop\Data\Suraya\V2085_EBA181_RH4_tryp_chymo\V2085_EBA181_P_tryp_2pmols_43C_70m09233648H4C4.raw #8636 RT: 49.67
FTMS, HCD, z=+3, Mono m/z=757.94763 Da, MH+=2271.82834 Da, Match Tol.=20 mmu

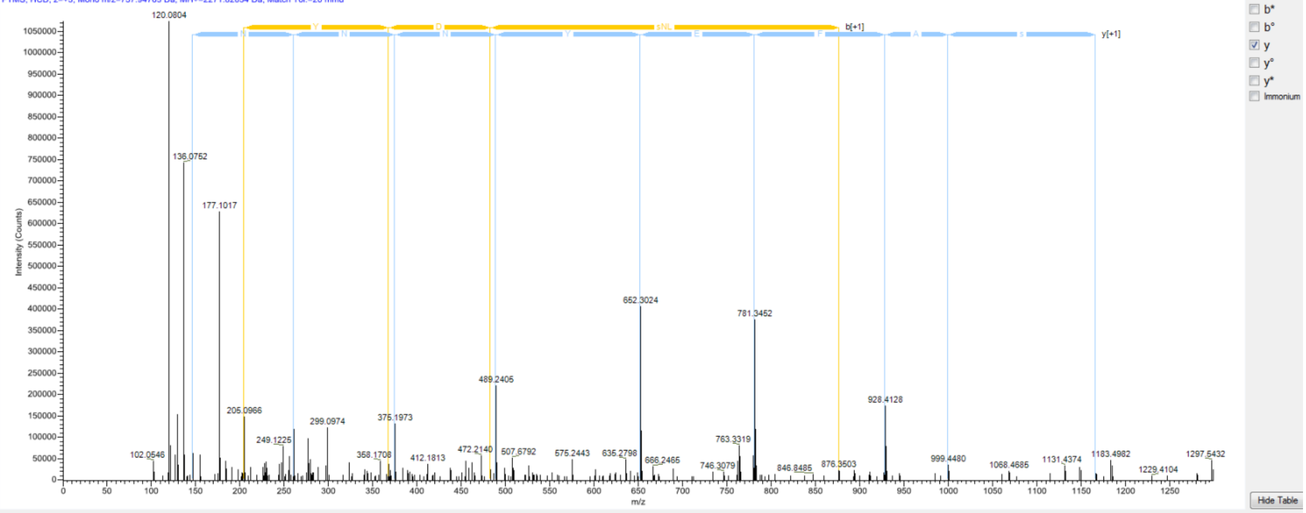

	(NH3+) -	F> <Y	Y> <D	D> <s	s> <N	N> <L	L> <N	N> <D	D> <s	s> <A	A> <F	F> <E	E> <Y	Y> <N	N> <N	N> <N	N> <K	K - (COOH)
b (+1)	58.02875	205.09717	368.16049	483.18744	650.18580	764.22873	877.31280	991.35573	1106.38268	1273.38104	1344.41816	1491.48658	1620.52918	1783.59250	1897.63543	2011.67836	2125.72129	2253.81626
b*(+1)	41.00220	188.07062	351.13394	466.16089	633.15925	747.20218	860.28625	974.32918	1089.35613	1256.35449	1327.39161	1474.46003	1603.50263	1766.56595	1880.60888	1994.65181	2108.69474	2236.78971
b°(+1)	40.01818	187.08660	350.14992	465.17687	632.17523	746.21816	859.30223	973.34516	1088.37211	1255.37047	1326.40759	1473.47601	1602.51861	1765.58193	1879.62486	1993.66779	2107.71072	2235.80569
y (+1)	2214.80535	2067.73693	1904.67361	1789.64666	1622.64830	1508.60537	1395.52130	1281.47837	1166.45142	999.45306	928.41594	781.34752	652.30492	489.24160	375.19867	261.15574	147.11281	2271.82682
y*(+1)	2197.77880	2050.71038	1887.64706	1772.62011	1605.62175	1491.57882	1378.49475	1264.45182	1149.42487	982.42651	911.38939	764.32097	635.27837	472.21505	358.17212	244.12919	130.08626	2254.80027
y°(+1)	2196.79479	2049.72637	1886.66305	1771.63610	1604.63774	1490.59481	1377.51074	1263.46781	1148.44086	981.44250	910.40538	763.33696	634.29436	471.23104	357.18811	243.14518	129.10225	2253.81626

Value Type: Theo. Mass [Da]

G F⌋Y⌋D⌋**s** N L⌋N D⌈**s**⌈A⌈F⌈E⌈Y⌈N⌈N⌈N⌈K

G. EBA181_21

Peptide Summary

Sequence: NLNDSAFEYNNNK, S5-Phospho (79.96633 Da)
Charge: +2, Monoisotopic m/z: 811.82678 Da (-1.01 mmu/-1.24 ppm), MH+: 1622.64629 Da, RT: 30.72 min,
Identified with: Mascot (v1.27); IonScore:76, Exp Value:2.5E-008, Ions matched by search engine: 8/144
Fragment match tolerance used for search: 20 mmu

Fragment Matches

Value Type: Theo. Mass [Da]

Ion Series | Phosphorylation Losses | Neutral Losses | Multiple Neutral Losses | Precursor Ions

#1	a⁺	a²⁺	b⁺	b²⁺	Seq.	y⁺	y²⁺	#2
1	87.05529	44.03128	115.05021	58.02874	N			13
2	200.13936	100.57332	228.13428	114.57078	L	1508.60537	754.80632	12
3	314.18229	157.59478	342.17721	171.59224	N	1395.52130	698.26429	11
4	429.20924	215.10826	457.20416	229.10572	D	1281.47837	641.24282	10
5	596.20760	298.60744	624.20252	312.60490	S-Phospho	1166.45142	583.72935	9
6	667.24472	334.12600	695.23964	348.12346	A	999.45306	500.23017	8
7	814.31314	407.66021	842.30806	421.65767	F	928.41594	464.71161	7
8	943.35574	472.18151	971.35066	486.17897	E	781.34752	391.17740	6
9	1106.41906	553.71317	1134.41398	567.71063	Y	652.30492	326.65610	5
10	1220.46199	610.73463	1248.45691	624.73209	N	489.24160	245.12444	4
11	1334.50492	667.75610	1362.49984	681.75356	N	375.19867	188.10297	3
12	1448.54785	724.77756	1476.54277	738.77502	N	261.15574	131.08151	2
13					K	147.11281	74.06004	1

Fragment Spectrum

Extracted from: C:\Users\SAH\Desktop\Data\Suraya\V2085_EBA181_RH4_tryp_chymo\V2085_EBA181_P_tryp_2pmols_43C_70m09233648H4C4.raw #4294 RT: 30.72
FTMS, HCD, z=+2, Mono m/z=811.82678 Da, MH+=1622.64629 Da, Match Tol.=20 mmu

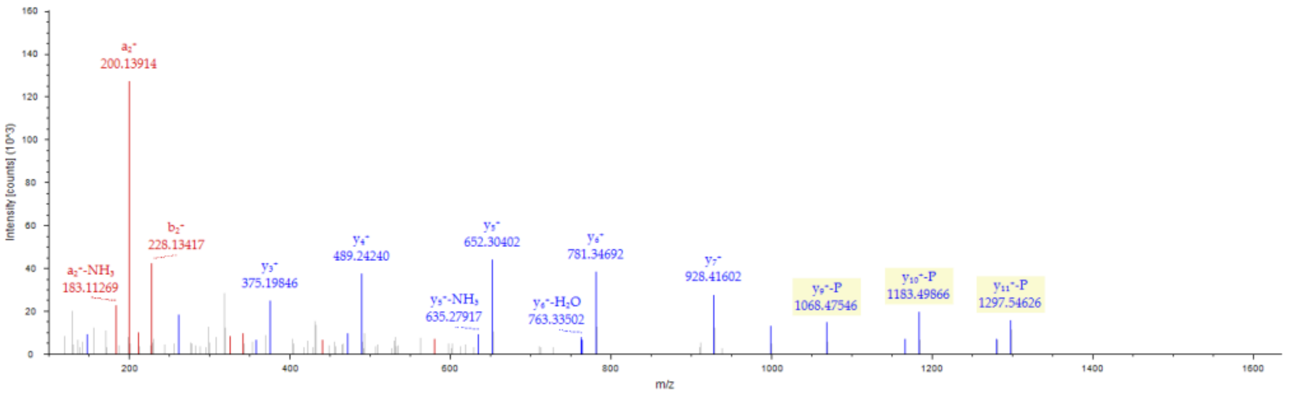

Sequence: NLNDSAFEYNNNK
S5-Phospho (79.96633 Da)
Charge: +2, Monoisotopic m/z: 811.82678 Da (-1.01 mmu/-1.24 ppm), MH+: 1622.64629 Da, RT: 30.72 min,

Extracted from: C:\Users\SAH\Desktop\Data\Suraya\V2085_EBA181_RH4_tryp_chymo\V2085_EBA181_P_tryp_2pmols_43C_70m09233648H4C4.raw #4294 RT: 30.72
FTMS, HCD, z=+2, Mono m/z=811.82678 Da, MH+=1622.64629 Da, Match Tol.=20 mmu

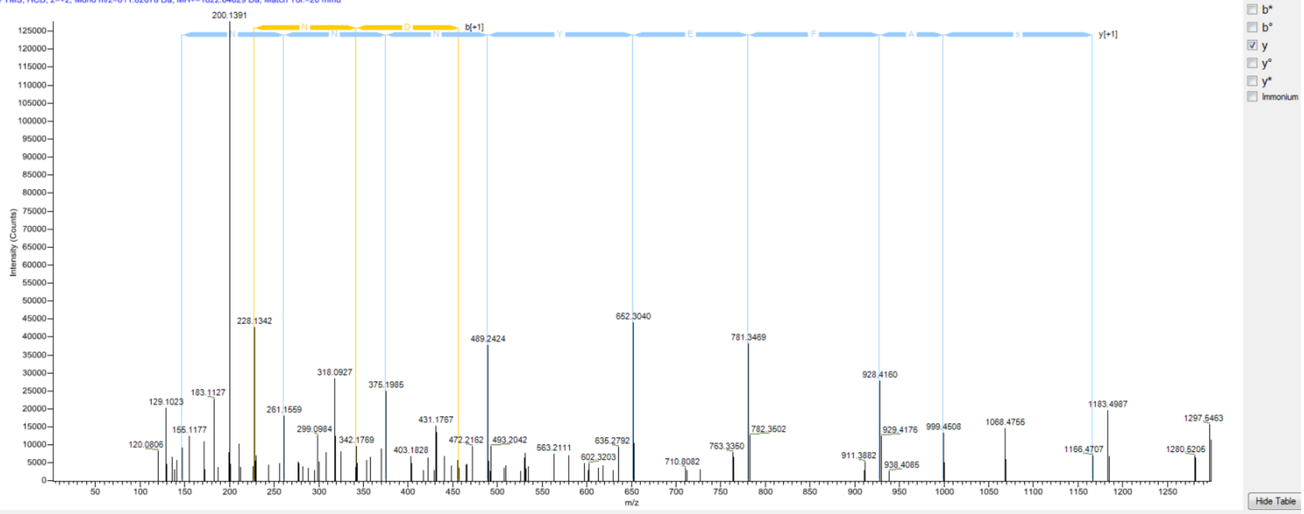

	(NH3+) -	L> <N	N> <D	D> <s	s> <A	A> <F	F> <E	E>	Y> <N	N> <N	N> <N	N> <K	K - (COOH)
b (+1)	115.05021	228.13428	342.17721	457.20416	624.20252	695.23964	842.30806	971.35066	1134.41398	1248.45691	1362.49984	1476.54277	1604.63774
b*(+1)	98.02366	211.10773	325.15066	440.17761	607.17597	678.21309	825.28151	954.32411	1117.38743	1231.43036	1345.47329	1459.51622	1587.61119
b°(+1)	97.03964	210.12371	324.16664	439.19359	606.19195	677.22907	824.29749	953.34009	1116.40341	1230.44634	1344.48927	1458.53220	1586.62717
y (+1)	1508.60537	1395.52130	1281.47837	1166.45142	999.45306	928.41594	781.34752	652.30492	489.24160	375.19867	261.15574	147.11281	1622.64830
y*(+1)	1491.57882	1378.49475	1264.45182	1149.42487	982.42651	911.38939	764.32097	635.27837	472.21505	358.17212	244.12919	130.08626	1605.62175
y°(+1)	1490.59481	1377.51074	1263.46781	1148.44086	981.44250	910.40538	763.33696	634.29436	471.23104	357.18811	243.14518	129.10225	1604.63774

Value Type: Theo. Mass [Da]

N L⌋N⌋D⌋s⌈A⌈F⌈E⌈Y⌈N⌈N⌈N⌈K
