# Supplementary material for: The Binding of Plasmodium falciparum Adhesins and Erythrocyte Invasion Proteins to Aldolase Is Enhanced by Phosphorylation
Source: PLoS One. 2016 Sep 8;11(9):e0161850. doi: 10.1371/journal.pone.0161850 (PMC5015959; doi:10.1371/journal.pone.0161850)
Supplement: S2 Fig — A. Phosphorylation of recombinant MTRAP peptide by protein kinase A. The MTRAP peptide (GPLGSMSGLNDIFEAQKIEWHEYFLRKEKTEKVVQEETKEENFEVMFNDDALKGKDNKAMDEEEFWALE; 8171.8) was biotinylated (8558.81; upper panel) and then phosphorylated using PKA (lower panel). The peaks corresponding to the protein and its phosphorylated forms are indicated. B. Phosphorylation of synthetic biotinylated AMA1 peptide by CDPK1 (upper panel) and protein kinase A (lower panel). The non-phosphorylated peptide (Biotin-YKRKGNAEKYDKMDEPQDYGKSNSRNDEMLDPEASFWGEEKRASHTTPVLMEKPYY, 6888.2) and a single phosphorylated form (6968.4) were detected. C. Phosphorylation of synthetic biotinylated RH1 peptide by CDPK1 (upper panel) and protein kinase A (lower panel). Only the non-phosphorylated peptide (Biotin-GKNNKQEYDKEQEKQQQNDFVCDNNKMDDKSTQKYGRNQEEVMEISFDNDYI, 6539.9) was detected after incubation with PKA and an additional single (6619.9) phosphorylated form was detected following incubation with CDPK1. D. Phosphorylation of synthetic biotinylated RH4 peptide by CDPK1 (upper panel), protein kinase A (middle panel) and casein kinase 2 (lower panel). Only the non-phosphorylated peptide (Biotin-KNSNEPHHIFNIFQKEFSEADNAHSEEKEEYLPVYFDEVEDEVEDEVEDEDENENEVENENEDFNDI, 8305.5) was detected after incubation with CDPK1 or PKA and a single (8386.2) phosphorylated form was detected following incubation with CK2. E. Phosphorylation of synthetic biotinylated EBA140 peptide by CDPK1 (upper panel) and protein kinase A (lower panel). Only the non-phosphorylated peptide (Biotin-RMGKSNEEYDIGESNIEATFEENNYLNKLSRIFNQEVQETNISDYSEYNYNEKNMY, 6995.8) was detected after incubation with PKA. F. Phosphorylation of synthetic biotinylated EBA175 peptide by CDPK1 (upper panel) and protein kinase A (lower panel). Only the non-phosphorylated peptide (Biotin-KYQSSEGVMNENNENNFLFEVTDNLDKLSNMFNQQVQETNINDFSEYHEDINDINFKK, 7163.2) was detected after incubation with PKA and a single (7243.2) phosphorylated form was detected following incubation wit [file pone.0161850.s002.pdf]

**Supporting Information Figure 2.**

**Phosphorylation in vitro of the cytoplasmic domain of proteins by CDPK1, protein kinase A and casein kinase 2, analysed by electrospray mass spectrometry.**

A. Phosphorylation of recombinant MTRAP peptide by protein kinase A. The MTRAP peptide (GPLGSMSGLNDIFEAQKIEWHEYFLRKEKTEKVVQEETKEENFEVMFNDDALKGKDNKAMDEEEFWALE; 8171.8)was biotinylated (8558.81; upper panel) and then phosphorylated using PKA (lower panel). The peaks corresponding to the protein and its phosphorylated forms are indicated.

B. Phosphorylation of synthetic biotinylated AMA1 peptide by CDPK1 (upper panel) and protein kinase A (lower panel). The non-phosphorylated peptide (Biotin-YKRKGNAEKYDKMDEPQDYGKSNSRNDEMLDPEASFWGEEKRASHTTPVLMEKPYY, 6888.2) and a single phosphorylated form (6968.4) were detected.

C. Phosphorylation of synthetic biotinylated RH1 peptide by CDPK1 (upper panel) and protein kinase A (lower panel). Only the non-phosphorylated peptide (Biotin-GKNNKQEYDKEQEKQQQNDFVCDNNKMDDKSTQKYGRNQEEVMEISFDNDYI, 6539.9) was detected after incubation with PKA and an additional single (6619.9)phosphorylated form was detected following incubation with CDPK1.

D. Phosphorylation of synthetic biotinylated RH4 peptide by CDPK1 (upper panel), protein kinase A (middle panel) and casein kinase 2 (lower panel). Only the non-phosphorylated peptide (Biotin-KNSNEPHHIFNIFQKEFSEADNAHSEEKEEYLPVYFDEVEDEVEDEVEDEDENENEVENENEDFNDI, 8305.5) was detected after incubation with CDPK1 or PKA and a single (8386.2) phosphorylated form was detected following incubation with CK2.

E. Phosphorylation of synthetic biotinylated EBA140 peptide by CDPK1 (upper panel) and protein kinase A (lower panel). Only the non-phosphorylated peptide (Biotin-RMGKSNEEYDIGESNIEATFEENNYLNKLSRIFNQEVQETNISDYSEYNYNEKNMY, 6995.8) was detected after incubation with PKA

F. Phosphorylation of synthetic biotinylated EBA175 peptide by CDPK1 (upper panel) and protein kinase A (lower panel). Only the non-phosphorylated peptide (Biotin-KYQSSEGVMNENNENNFLFEVTDNLDKLSNMFNQQVQETNINDFSEYHEDINDINFKK, 7163.2)was detected after incubation with PKA and a single (7243.2)phosphorylated form was detected following incubation with CDPK1.

G. Phosphorylation of synthetic biotinylated EBA181 peptide by CDPK1 (upper panel) and protein kinase A (lower panel). Only the non-phosphorylated peptide (Biotin-RKNLDDEKGFYDSNLNDSAFEYNNNKYNKLPYMFDQQINVVNSDLYSEGIYDDTTTF, 7014.0) was detected after incubation with PKA and single (7094.9) and dual (7173.9) phosphorylated forms were detected following incubation with CDPK1.

A. MTRAP

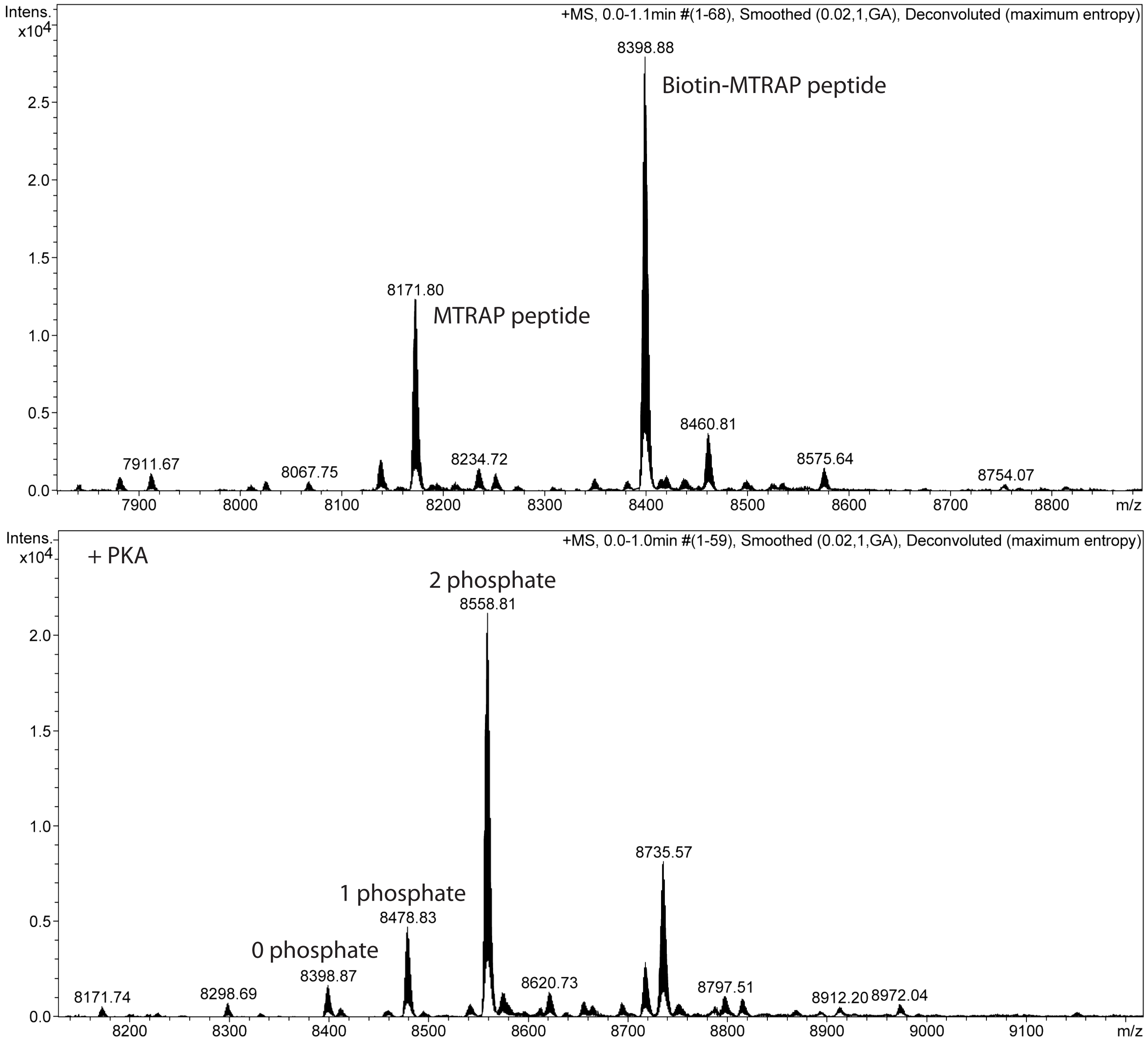
Intens.
x10^4
+MS, 0.0-1.1min #(1-68), Smoothed (0.02,1,GA), Deconvoluted (maximum entropy)
8398.88
Biotin-MTRAP peptide
8171.80
MTRAP peptide
8460.81
7911.67
8067.75
8234.72
8575.64
8754.07
0.0
0.5
1.0
1.5
2.0
2.5
7900
8000
8100
8200
8300
8400
8500
8600
8700
8800
m/z
Intens.
x10^4
+ PKA
+MS, 0.0-1.0min #(1-59), Smoothed (0.02,1,GA), Deconvoluted (maximum entropy)
2 phosphate
8558.81
8735.57
1 phosphate
8478.83
0 phosphate
8398.87
8171.74
8298.69
8620.73
8797.51
8912.20
8972.04
0.0
0.5
1.0
1.5
2.0
8200
8300
8400
8500
8600
8700
8800
8900
9000
9100
m/z

B. AMA1

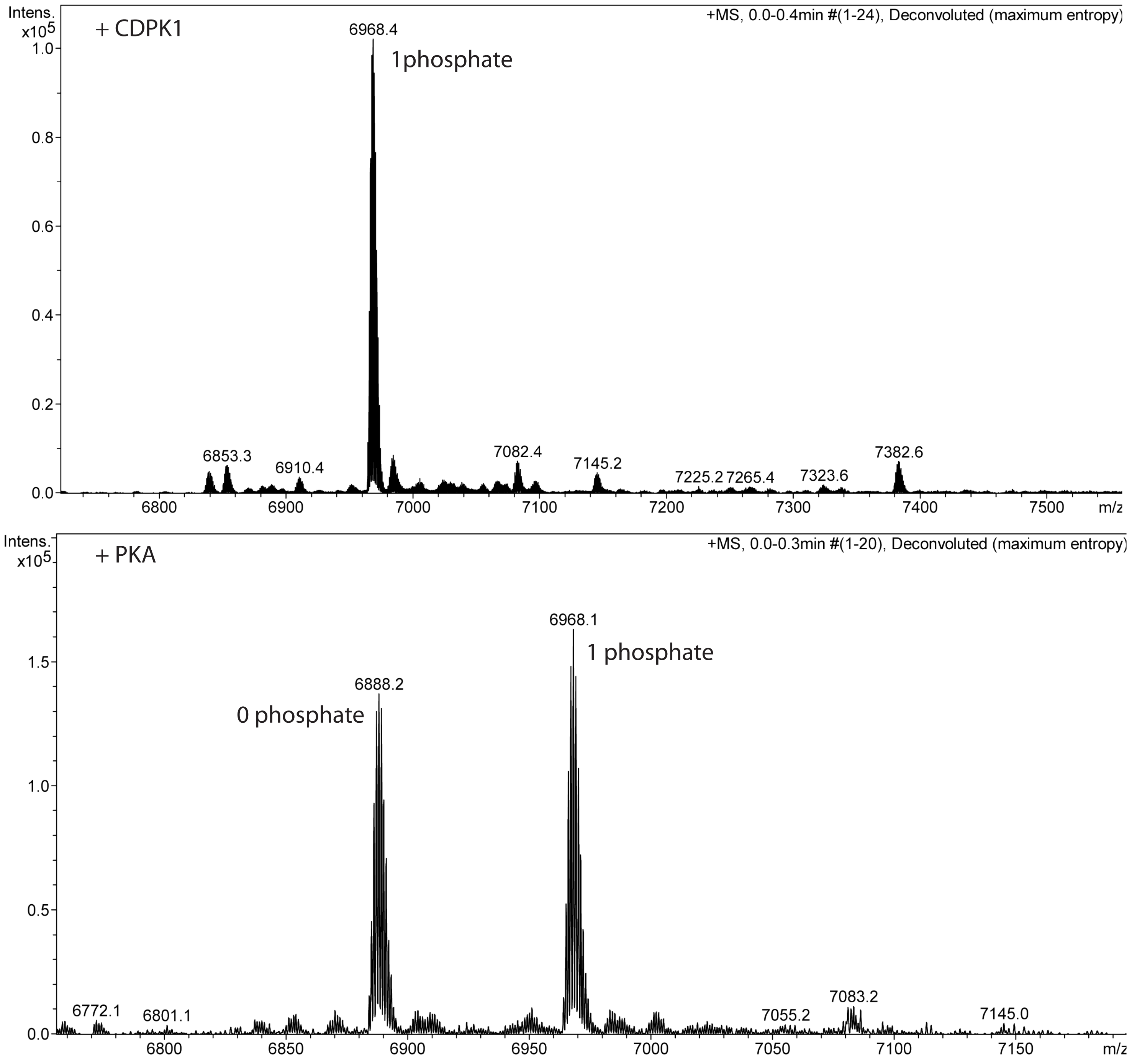

Intens.
x10^5
+ CDPK1
+MS, 0.0-0.4min #(1-24), Deconvoluted (maximum entropy)
6968.4
1phosphate
6853.3
6910.4
7082.4
7145.2
7225.2
7265.4
7323.6
7382.6
1.0
0.8
0.6
0.4
0.2
0.0
6800
6900
7000
7100
7200
7300
7400
7500
m/z
Intens.
x10^5
+ PKA
+MS, 0.0-0.3min #(1-20), Deconvoluted (maximum entropy)
6968.1
1 phosphate
6888.2
0 phosphate
6772.1
6801.1
7055.2
7083.2
7145.0
1.5
1.0
0.5
0.0
6800
6850
6900
6950
7000
7050
7100
7150
m/z

C. RH1

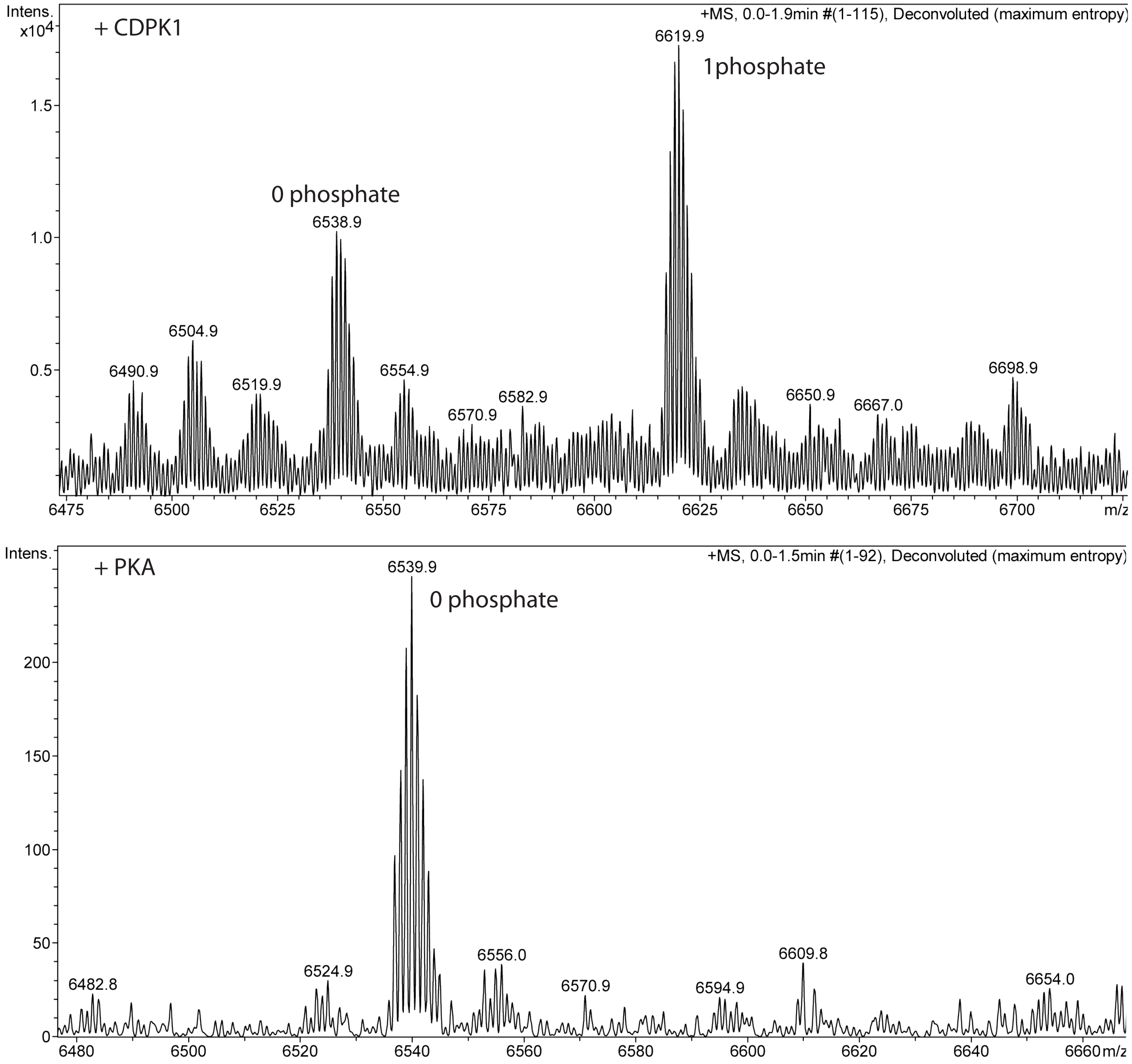

Intens.
x10^4
+ CDPK1
+MS, 0.0-1.9min #(1-115), Deconvoluted (maximum entropy)
6619.9
1phosphate
0 phosphate
6538.9
6504.9
6490.9
6519.9
6554.9
6570.9
6582.9
6650.9
6667.0
6698.9
1.5
1.0
0.5
6475
6500
6525
6550
6575
6600
6625
6650
6675
6700
m/z
Intens.
+ PKA
+MS, 0.0-1.5min #(1-92), Deconvoluted (maximum entropy)
6539.9
0 phosphate
200
150
100
50
0
6482.8
6524.9
6556.0
6570.9
6594.9
6609.8
6654.0
6480
6500
6520
6540
6560
6580
6600
6620
6640
6660
m/z

D. RH4

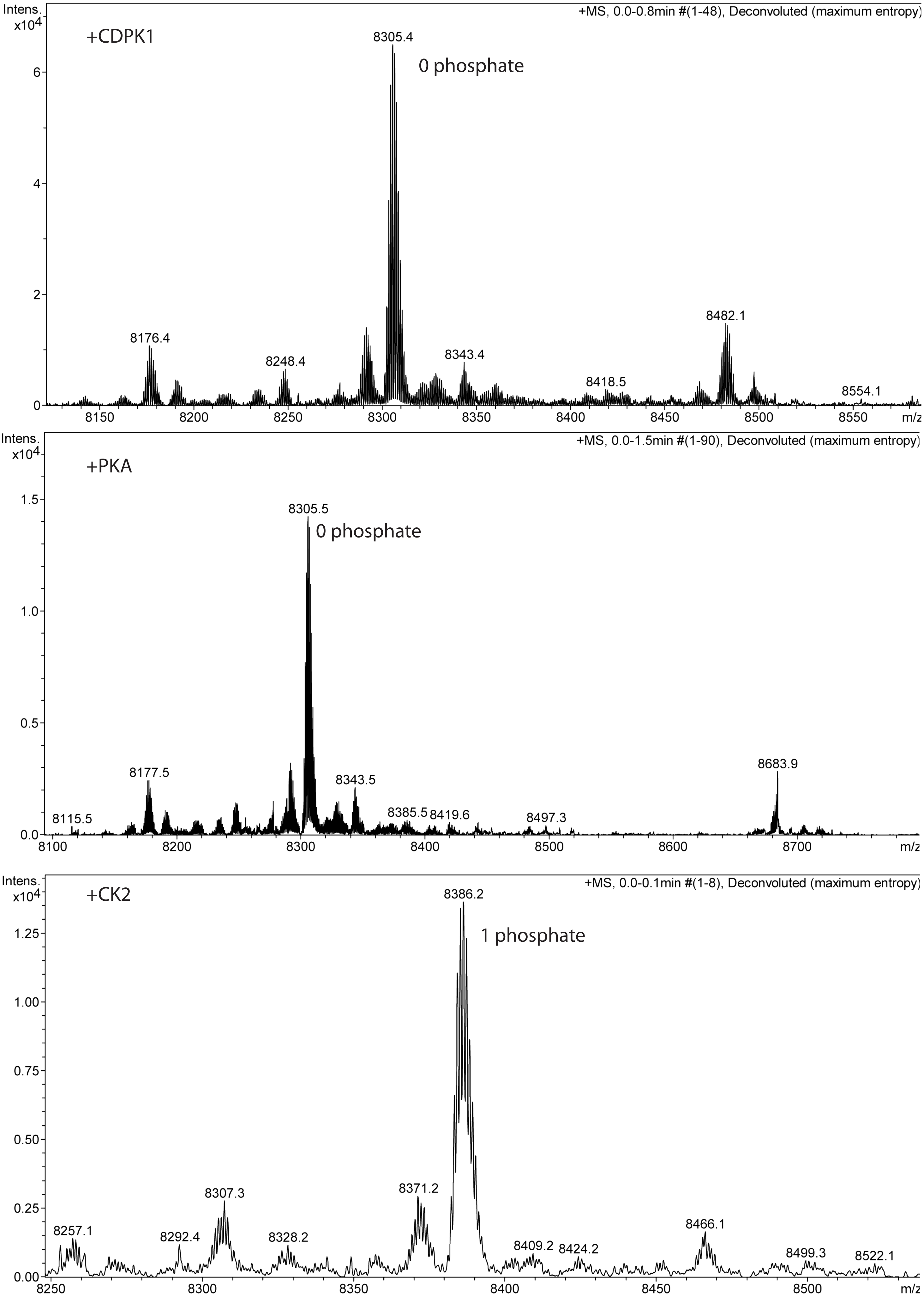

Intens. x10^4
+MS, 0.0-0.8min #(1-48), Deconvoluted (maximum entropy)
+CDPK1
8305.4
0 phosphate
8176.4
8248.4
8343.4
8418.5
8482.1
8554.1
m/z
Intens. x10^4
+MS, 0.0-1.5min #(1-90), Deconvoluted (maximum entropy)
+PKA
8305.5
0 phosphate
8115.5
8177.5
8343.5
8385.5
8419.6
8497.3
8683.9
m/z
Intens. x10^4
+MS, 0.0-0.1min #(1-8), Deconvoluted (maximum entropy)
+CK2
8386.2
1 phosphate
8257.1
8292.4
8307.3
8328.2
8371.2
8409.2
8424.2
8466.1
8499.3
8522.1
m/z

E. EBA140

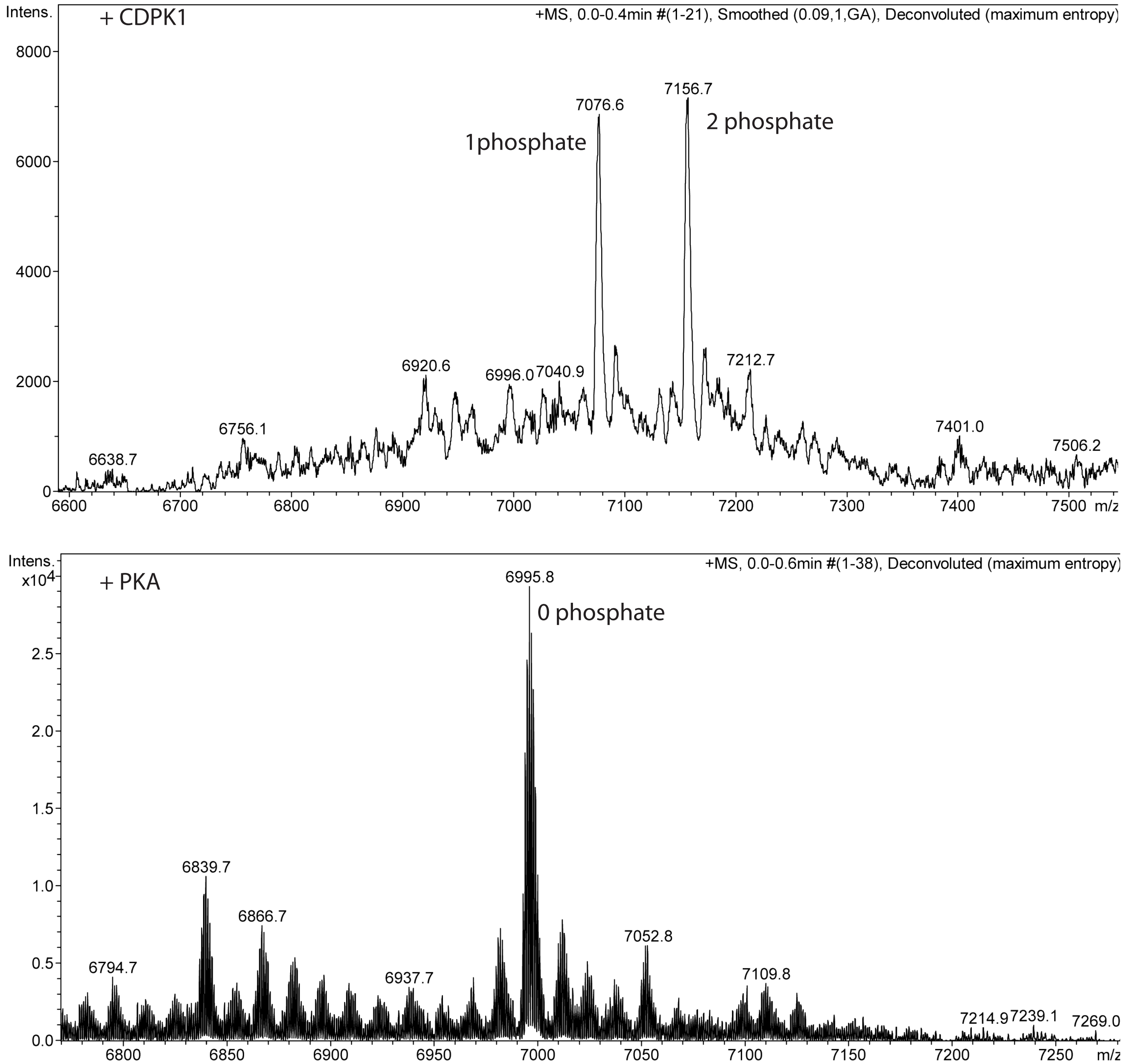
Intens.
+ CDPK1
+MS, 0.0-0.4min #(1-21), Smoothed (0.09,1,GA), Deconvoluted (maximum entropy)
8000
6000
4000
2000
0
7076.6
1phosphate
7156.7
2 phosphate
6638.7
6756.1
6920.6
6996.0
7040.9
7212.7
7401.0
7506.2
6600
6700
6800
6900
7000
7100
7200
7300
7400
7500
m/z
Intens.
x10⁴
+ PKA
+MS, 0.0-0.6min #(1-38), Deconvoluted (maximum entropy)
6995.8
0 phosphate
2.5
2.0
1.5
1.0
0.5
0.0
6839.7
6866.7
6794.7
6937.7
7052.8
7109.8
7214.9
7239.1
7269.0
6800
6850
6900
6950
7000
7050
7100
7150
7200
7250
m/z

F. EBA175

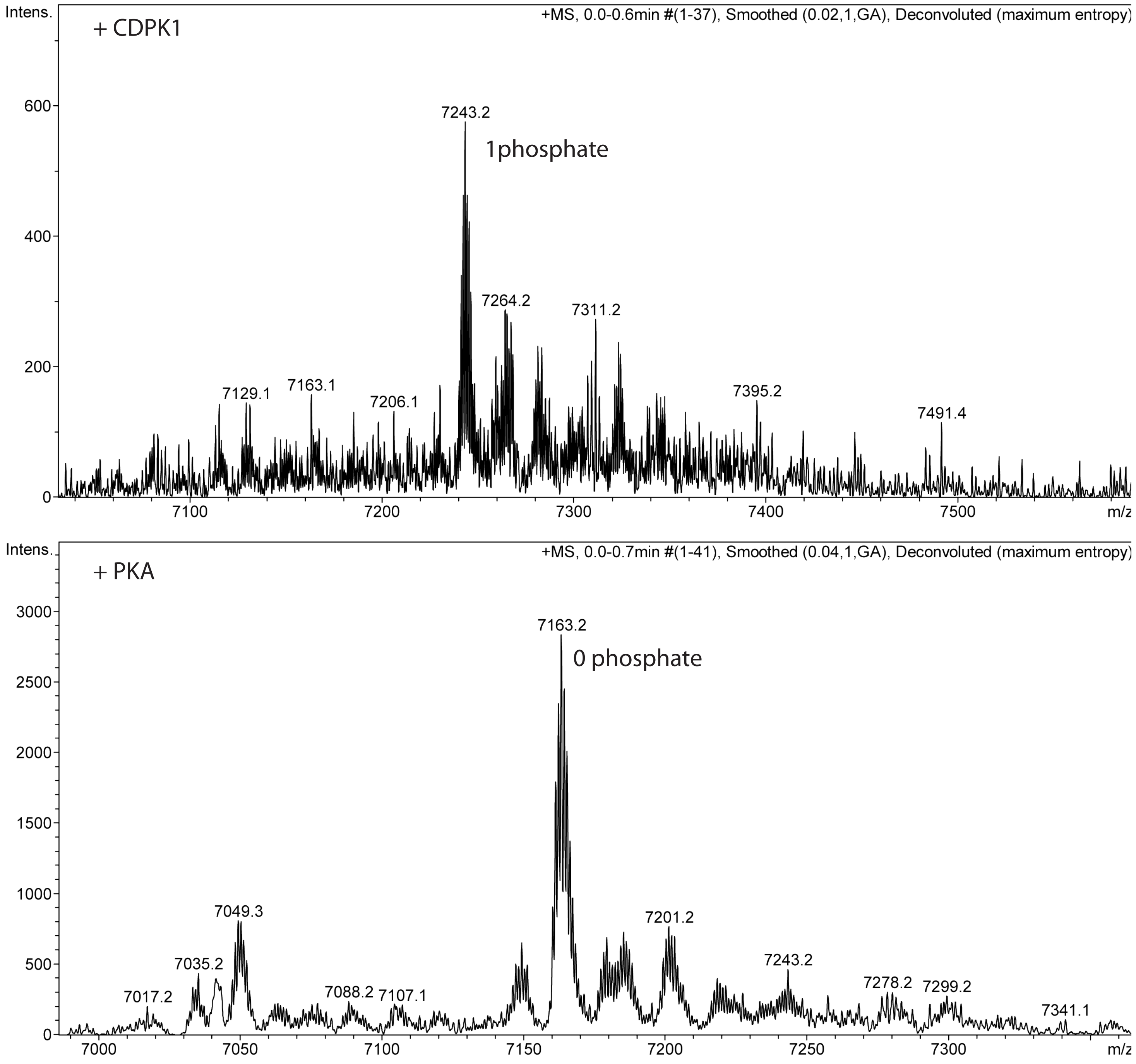

Intens.
+MS, 0.0-0.6min #(1-37), Smoothed (0.02,1,GA), Deconvoluted (maximum entropy)
+ CDPK1
7243.2
1phosphate
7264.2
7311.2
7129.1
7163.1
7206.1
7395.2
7491.4
m/z
Intens.
+MS, 0.0-0.7min #(1-41), Smoothed (0.04,1,GA), Deconvoluted (maximum entropy)
+ PKA
7163.2
0 phosphate
7049.3
7201.2
7035.2
7017.2
7088.2
7107.1
7243.2
7278.2
7299.2
7341.1
m/z

G. EBA181

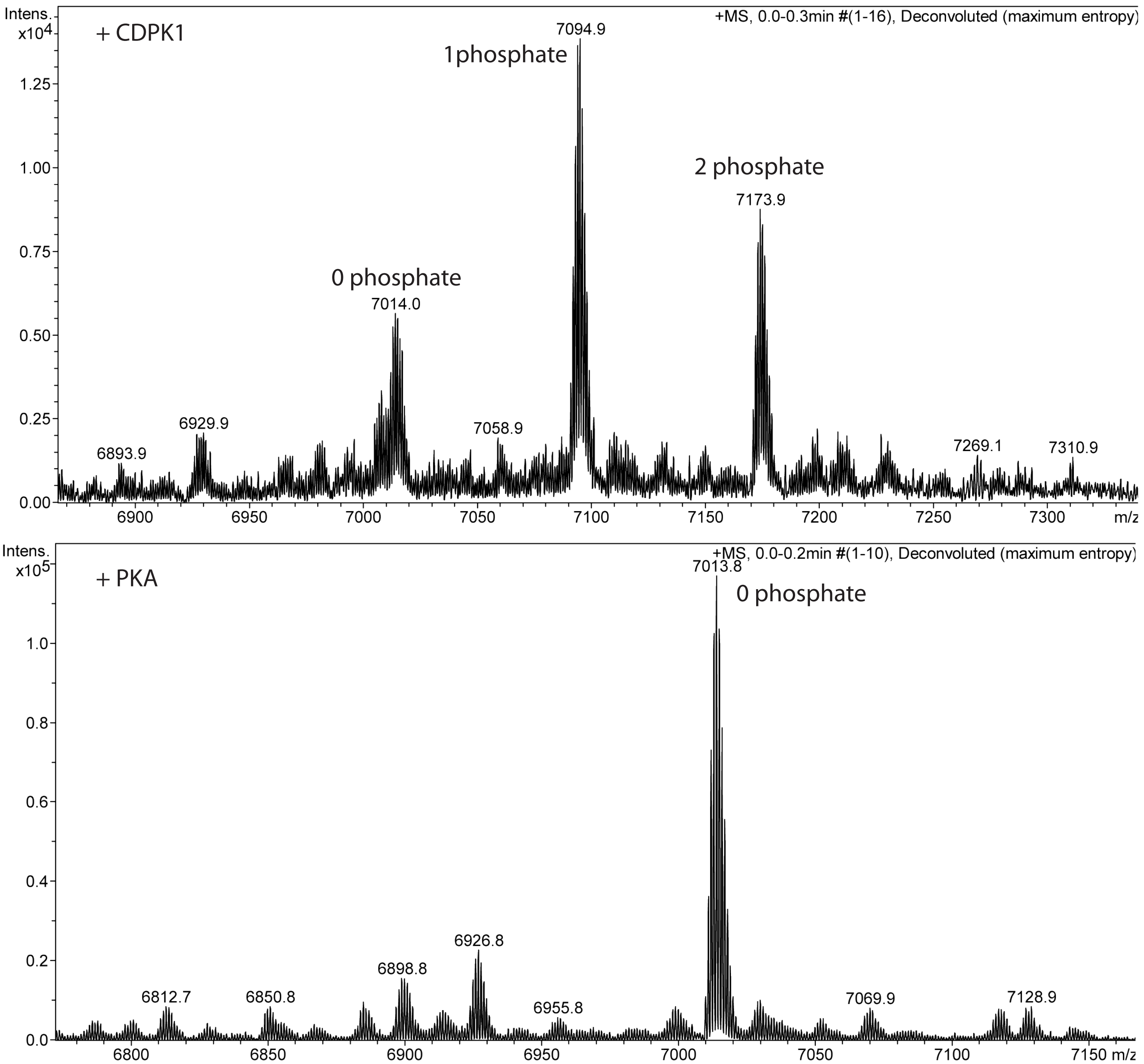

Intens.
x10^4
+ CDPK1
+MS, 0.0-0.3min #(1-16), Deconvoluted (maximum entropy)
7094.9
1phosphate
2 phosphate
7173.9
0 phosphate
7014.0
6929.9
6893.9
7058.9
7269.1
7310.9
1.25
1.00
0.75
0.50
0.25
0.00
6900
6950
7000
7050
7100
7150
7200
7250
7300
m/z
Intens.
x10^5
+ PKA
+MS, 0.0-0.2min #(1-10), Deconvoluted (maximum entropy)
7013.8
0 phosphate
6926.8
6898.8
6812.7
6850.8
6955.8
7069.9
7128.9
1.0
0.8
0.6
0.4
0.2
0.0
6800
6850
6900
6950
7000
7050
7100
7150 m/z
